# Simultaneously targeting extracellular vesicle trafficking and TGF-β receptor kinase activity blocks signaling hyperactivation and metastasis

**DOI:** 10.1038/s41392-023-01711-1

**Published:** 2023-12-18

**Authors:** Adilson Fonseca Teixeira, Yanhong Wang, Josephine Iaria, Peter ten Dijke, Hong-Jian Zhu

**Affiliations:** 1 Department of Surgery (The Royal Melbourne Hospital), The University of Melbourne, Parkville, VIC Australia; 2 Huagene Institute, Kecheng Science and Technology Park, Pukou District, Nanjing, Jiangsu China; 3 Department of Cell and Chemical Biology, Oncode Institute, Leiden University Medical Center, Leiden, The Netherlands

**Keywords:** Breast cancer, Cancer therapy, Metastasis, Preclinical research, Translational research

## Abstract

Metastasis is the leading cause of cancer-related deaths. Transforming growth factor beta (TGF-β) signaling drives metastasis and is strongly enhanced during cancer progression. Yet, the use of on-target TGF-β signaling inhibitors in the treatment of cancer patients remains unsuccessful, highlighting a gap in the understanding of TGF-β biology that limits the establishment of efficient anti-metastatic therapies. Here, we show that TGF-β signaling hyperactivation in breast cancer cells is required for metastasis and relies on increased small extracellular vesicle (sEV) secretion. Demonstrating sEV’s unique role, TGF-β signaling levels induced by sEVs exceed the activity of matching concentrations of soluble ligand TGF-β. Further, genetic disruption of sEV secretion in highly-metastatic breast cancer cells impairs cancer cell aggressiveness by reducing TGF-β signaling to nearly-normal levels. Otherwise, TGF-β signaling activity in non-invasive breast cancer cells is inherently low, but can be amplified by sEVs, enabling invasion and metastasis of poorly-metastatic breast cancer cells. Underscoring the translational potential of inhibiting sEV trafficking in advanced breast cancers, treatment with dimethyl amiloride (DMA) decreases sEV secretion, TGF-β signaling activity, and breast cancer progression in vivo. Targeting both the sEV trafficking and TGF-β signaling by combining DMA and SB431542 at suboptimal doses potentiated this effect, normalizing the TGF-β signaling in primary tumors to potently reduce circulating tumor cells, metastasis, and tumor self-seeding. Collectively, this study establishes sEVs as critical elements in TGF-β biology, demonstrating the feasibility of inhibiting sEV trafficking as a new therapeutic approach to impair metastasis by normalizing TGF-β signaling levels in breast cancer cells.

## Abstract (web summary)

Breast cancer metastasis requires TGF-β signaling hyperactivation that relies on extracellular vesicle secretion and is impaired by inhibitors targeting extracellular vesicle trafficking.

## Introduction

Metastasis is the main cause of cancer-related deaths for breast cancer patients.^1–3^ Transforming Growth Factor-beta (TGF-β) and its signaling pathway are major drivers of metastasis^3–5^ and increased TGF-β signaling activity is associated with reduced survival for breast cancer patients.^6,7^ Accordingly, therapeutics, such as the TGF-β receptor Type I (TβRI) kinase inhibitor SB431542, have been developed to target the TGF-β signaling for treatment of human diseases, including cancer.^3,8,9^ However, despite successful on-targeting in pre-clinical models, there has not been such success in cancer clinical trials.^10^ Further understanding the mechanisms that drive TGF-β signaling increase, may underscore the clinical failure of TGF-β inhibitors.

TGF-β signaling is activated upon binding of the soluble ligand TGF-β to TGF-β receptors Type I and II (TβRI and TβRII), leading to TβRII-mediated TβRI phosphorylation.^11,12^ If not inhibited by SMAD7, activated TβRI phosphorylates SMAD2/3, inducing its association with SMAD4.^13,14^ The hetero-oligomers formed by SMAD2/3 and SMAD4 accumulate in the nucleus, regulating the expression of TGF-β target genes.^15,16^ TGF-β has also been found in small extracellular vesicles (sEV), also termed as exosomes,^17–20^ and sEVs containing TGF-β activity promotes cancer cell epithelial-mesenchymal transition (EMT) in vitro.^17^ Yet, the physiological relevance of TGF-β-containing sEVs in cancer progression remains largely unknown.

sEVs mediate cell-cell communication by transporting different types of cargos.^21^ EV subpopulations are distinguished according to biogenesis (e.g., exosomes and microvesicles), physical properties (e.g., low and high density EVs), or size (e.g., small and medium/large EVs).^22^ Additionally, molecular markers (e.g., Alix, TSG101 and CD63) are useful to characterize EVs.^22^ The biogenesis of exosomes (50–150 nm) is a multistep process. Cargo sorting occurs via endosomal sorting complexes required for transport (ESCRT)-dependent or -independent mechanisms, and it is followed by generation of intraluminal vesicles (ILVs) into late endosomes (multivesicular bodies; MVBs). Trafficking, docking, and fusion of MVBs with the plasma membrane enables the secretion of ILVs as exosomes.^23^ Yet, the activity mediated by exosomes on target cells requires additional steps, including the interaction and the internalization of exosomes by recipient cells. Genetic manipulation of sEV trafficking has established a strong association between exosome secretion/uptake and cancer cell aggressiveness. For example, Rab27a regulates the exocytosis of exosomes and Rab27a knockdown impairs sEV secretion in cancer models.^24^ Drugs such as dimethyl amiloride (DMA), heparin and 4-nitrophenyl β-D-xylopyranoside (PNP-Xyl) have also been used for a similar purpose.^25–27^ Still, few studies have investigated the use of these drugs in vivo and little is known about their activity on pro-metastatic signaling pathways.

Here, we hypothesize that increased sEV trafficking underlies the amplification of TGF-β signaling levels in highly invasive breast cancer cells. Consequently, disrupting sEV secretion/uptake can be an effective therapeutic strategy to impair TGF-β-induced metastasis. Our results demonstrate that sEVs are crucial to cancer progression by driving the hyperactivation of the TGF-β signaling in breast cancer cells, which helps to explain the failure of TGF-β signaling inhibitors in cancer clinical trials. EV-induced TGF-β signaling activity induces cancer cell aggressiveness, increasing the progression of highly-invasive breast cancers, and enabling metastasis of weakly-invasive breast cancer cells. Inhibiting sEV trafficking decreased TGF-β signaling activity in cancer cells both in vitro and in vivo and impaired cancer progression. Moreover, combining DMA with SB431542 at suboptimal doses further reduced circulating tumor cells (CTCs), metastasis, and tumor self-seeding in vivo. These results identify sEVs as major players in TGF-β biology that may be pursued as therapeutic targets for the treatment of cancer patients.

## Results

### Elevated expression of exosome-related genes correlates with breast cancer progression and poor prognosis for breast cancer patients

**Fig. 1:**
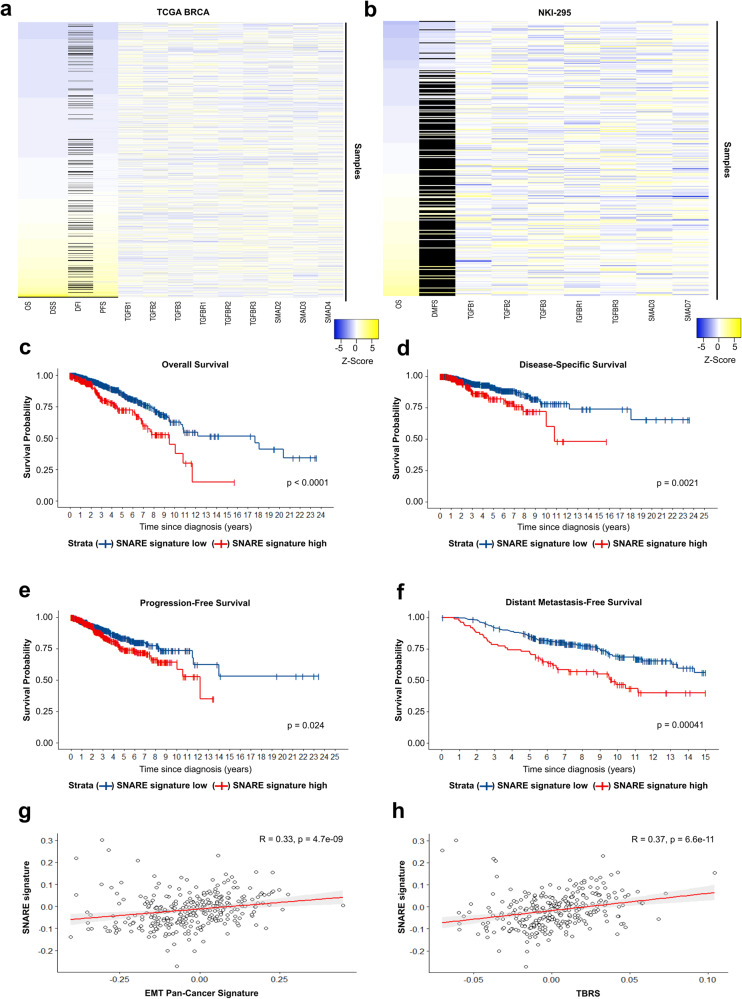
Elevated expression of exosome-related genes correlates with breast cancer progression and poor prognosis for breast cancer patients. Heatmaps correlating breast cancer patient outcome [overall survival (OS), disease-specific survival (DSS), disease-free interval (DFI), progression-free survival (PFS), and distant metastasis-free survival (DMFS)] with the expression of TGF-β signaling components in the (**a**) TCGA cohort and (**b**) NKI-295 cohort. Black lines show missing data. Kaplan Meier curves showing (**c**) OS, (**d**) DSS, (**e**) PFS, and (**f**) DMFS for breast cancer patients stratified by the SNARE-related gene signature. TCGA cohort analyzed in (**c**–**e**), NKI-295 cohort analyzed in (**f**). Log-rank test was used to analyze survival probability in (**c–f**). Correlation between SNARE-related gene signature and (**g**) EMT pan-cancer gene expression signature or (**h**) the TGF-β-responsive gene signature (TBRS) in breast cancers calculated according to the Spearman’s rank correlation coefficient (NKI-295 cohort)

Alterations in TGF-β signaling activity have often been attributed to the deregulated expression of TGF-β signaling components.^4,28,29^ Interestingly, however, analysis of publicly available databases revealed only minor correlations between breast cancer patient survival and the expression of such molecules (i.e., ligands, receptors and SMADs) in breast cancers (Fig. 1a, b). We argued that alterations in the expression of these components cannot satisfactorily explain the elevation in TGF-β signaling levels seen in invasive breast cancer cells. Since EVs contain TGF-β activity and increased EV secretion correlates with cancer progression,^17–20,30–35^ we hypothesized that exosomes could impact the TGF-β signaling by increasing its activity levels in cancer cells. To evaluate this hypothesis, we analyzed the expression of 137 exosome-related genes (Supplementary table 1–4) in the TCGA BRCA dataset.^36^ Importantly, mutations in the analyzed genes were rarely observed in breast cancers (Supplementary table 5). Next, to minimize misinterpretations caused by the evaluation of individual genes, we integrated the expression of selected genes and established signature scores for related biological processes (Supplementary table 1, Supplementary Fig. 1a). Signature I included genes playing roles in endosome maturation (e.g., Rab GTPases). Signature II was enriched in members of the retromer complex (e.g., vacuolar protein sorting-associated protein (VSP) family members). Signature III was associated with cargo sorting (e.g., ESCRT members). Signature IV contained genes controlling the fusion of intracellular vesicles with the plasma membrane (e.g., SNARE members). Noteworthy, all exosome-related gene signatures were positively correlated to each other (Supplementary Fig. 1b).

Next, patients were stratified regarding high or low exosome-related signature scores. Survival analysis showed that signatures I (Supplementary Fig. 1d), II (Supplementary Fig. 1e), and III (Supplementary Fig. 1f) were not associated or only weakly associated with the overall survival (OS) of breast cancer patients in the TCGA BRCA cohort. In contrast, increased signature IV (SNARE complex) score was correlated with poor OS for these patients (Fig. 1c and Supplementary Fig. 1g). Additionally, elevated signature IV score was also associated with worse disease-specific survival (DSS) (Fig. 1d) and reduced progression-free survival (PFS) (Fig. 1e). This pattern was also confirmed in the NKI-295 cohort^37^ (Supplementary Fig. 1h–k, Supplementary Fig. 2a–d, Supplementary table 6, 7). Dataset analysis reinforced the positive correlation between all exosome-related gene signatures (Supplementary Fig. 1c), while a negative association was observed between signature IV score and patient OS (Supplementary Fig. 1k). Moreover, whereas the TCGA cohort shows few late-stage cancer patients, about 40% of NKI-295 patients developed metastasis. Remarkably, a strong association was seen between increased signature IV score and worse distant metastasis-free survival (DMFS) in this cohort (Fig. 1f). Further, advanced tumors showed increased scores compared to early-stage counterparts (Supplementary Fig. 2e–g). Multivariate Cox analysis confirmed an independent association between higher signature IV score and reduced OS (Supplementary Table 8, 9) or DMFS (Supplementary Table 10) of breast cancer patients.

We previously showed that a signature score composed of TGF-β responsive genes (TBRS) (Supplementary Table 11) is inversely correlated with breast cancer patient OS and DMFS.^6^ Using a previously established EMT signature (Supplementary Table 12),^38^ we observed its positive correlation with the signature IV (Fig. 1g). In agreement with these results, signature IV scores were also increased in breast cancers with higher TBRS (Fig. 1h).

Since signature IV was the best correlated with breast cancer patient outcome, this may imply that cancer progression is mainly impacted by alterations in exosome secretion regulated by SNARE activity. Moreover, the correlation between signature IV, EMT and TGF-β signaling scores suggests a cooperation between these biological processes to drive breast cancer metastasis.

### sEVs hyperactivate the TGF-β signaling activity in breast cancer cells in vitro

**Fig. 2:**
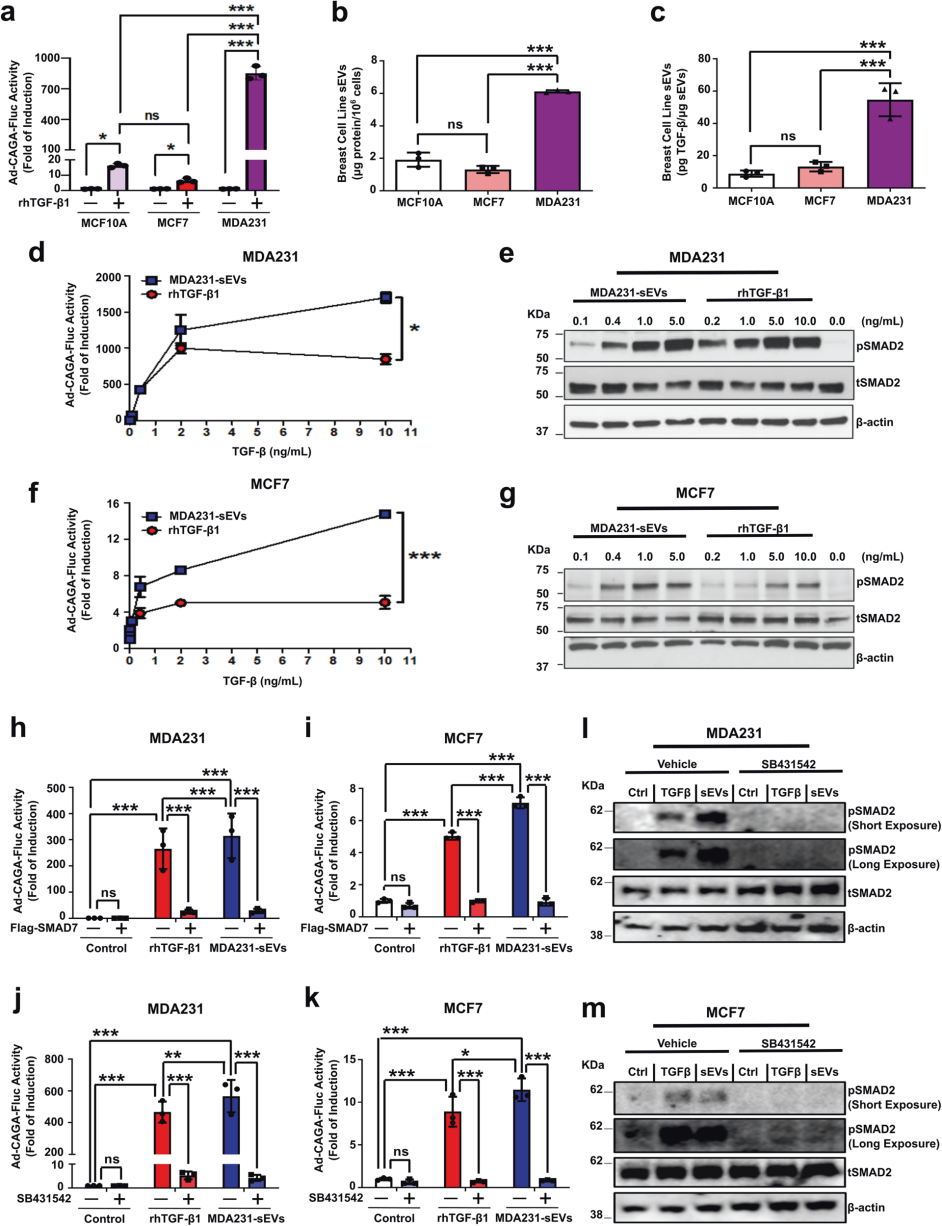
sEVs hyperactivate the TGF-β signaling activity in breast cancer cells in vitro. **a** TGF-β/SMAD3 signaling reporter (Ad-CAGA-Fluc) activity quantified in breast cell lines treated ±5 ng/mL rhTGF-β1 for 24 h. **b** sEV secretion was quantified by BCA assay. **c** TGF-β activity in sEVs secreted by breast cell lines was quantified and normalized per µg of sEV total protein. TGF-β/SMAD3 signaling reporter (Ad-CAGA-Fluc) activity quantified in (**d**) MDA231 and (**f**) MCF7 cells treated with rhTGF-β1 or MDA231-sEVs (increasing concentrations). Results were normalized by Gaussia luciferase (Ad-CMV-Gluc) activity. Phosphorylated (p)SMAD2 levels analyzed in (**e**) MDA231 and (**g**) MCF7 cells treated with rhTGF-β1 or MDA231-sEVs (1 h; increasing concentrations). Total (t)SMAD2 and β-actin were used as loading controls. **h**–**k** TGF-β/SMAD3 signaling activity was quantified in cancer cells as in (**a**). **h** MDA231 and (**i**) MCF7 cells infected with Ad-CMV-Flag-SMAD7. Ad-CMV-GFP: control adenovirus. **j** MDA231 and (**k**) MCF7 cells treated with SB431542 ± rhTGF-β1 or MDA231-sEVs. DMSO: vehicle for SB431452. pSMAD2 levels were evaluated in (**l**) MDA231 and (**m**) MCF7 cells treated with rhTGF-β1 or MDA231-sEVs ± SB431542 as in (**e**, **g**). DMSO: vehicle for SB431542. Results represent mean ± SD (*n* ≥ 3). One-way ANOVA test followed by Dunn’s Multiple Comparison test were used to analyze data in **(a**–**c**, **h**–**k)**. Unpaired Student’s *t*-test was used to analyze data in (**d**, **f**). ns: statistically non-significant, *p < 0.05, **p < 0.01, ***p < 0.001

To investigate if the correlation seen in human breast cancers could be recapitulated in vitro, we analyzed TGF-β signaling levels in breast cell lines. As quantified by luciferase assay, non-malignant MCF10A breast cells and poorly-invasive MCF7 breast cancer cells treated with recombinant human TGF-β1 (rhTGF-β1) showed low TGF-β/SMAD3 signaling activity. However, this was dramatically increased in highly-invasive MDA-MB-231 (hence forward MDA231) breast cancer cells. More specifically, MDA231 cells treated with rhTGF-β1 exhibited more than 50-fold increase in TGF-β signaling activity when compared with MCF10A cells and nearly 100-fold increase compared with MCF7 cells (Fig. 2a). Interestingly, as determined in breast cancer datasets (Fig. 1h), enhanced TGF-β signaling activity was also positively correlated with increased sEV secretion in vitro as quantified by total sEV protein through BCA assay (Fig. 2b and Supplementary Fig. 3a). Importantly, if normalized by total protein levels, sEVs secreted by MDA231 cells also contained higher TGF-β activity levels than the nanovesicles secreted by MCF10A and MCF7 cells (Fig. 2b, c and Supplementary Fig. 3a). This demonstrates that the levels of sEVs containing TGF-β activity that are secreted by highly-invasive breast cancer cells are greatly superior than those secreted by normal and poorly-invasive counterparts.

Next, we validated MDA231-sEV’s morphology (Supplementary Fig. 3b) and the expression of the EV markers Alix, TSG101, and CD63 (Supplementary Fig. 3c). Strikingly, whereas sEVs potently activated the TGF-β/SMAD3 signaling in recipient cells, this was not observed for large EVs and EV-depleted conditioned medium (CM) (Supplementary Fig. 3d). To evaluate whether TGF-β treatment impact sEV secretion, MDA231 cells were incubated with (TGF-β^+^) or without (TGF-β^−^) rhTGF-β1 (5 ng/mL) for 2 h prior to CM harvesting, and isolated sEVs were quantified. No significant changes were shown for sEV secretion regarding total protein content or size distribution, although TGF-β treatment was shown to reduce the number of particles secreted/cell (Supplementary Fig. 3e–g). Whereas TGF-β activity was detected in sEVs released by MDA231 cells in both conditions, rhTGF-β1 treatment potentiated this effect (Supplementary Fig. 3h, i). Moreover, while TGF-β^−^ sEVs induced cell migration, this effect was increased by treatment with TGF-β^+^ sEVs (Supplementary Fig. 3j). Noteworthy, while sEVs strongly activated the TGF-β signaling, we could not detect the activation of other related molecular pathways in recipient cells (Supplementary Fig. 4a, b).

We next sought to compare the response triggered in breast cancer cells by treatment with TGF-β in its soluble form (rhTGF-β1) or vesicular form (sEVs). TGF-β signaling activity was quantified in breast cancer cells treated with rhTGF-β1 or MDA231-sEVs. The concentration of TGF-β in MDA231-sEVs was normalized by TGF-β activity, while increasing concentrations of rhTGF-β1 were used as matching controls here and in following experiments. Based on our previous results (Supplementary Fig. 3e–j), we considered a range of 0.2–20.0 µg of total MDA231-sEV protein (approximately 0.1–10.0 ng of TGF-β activity) to be used as a parameter in this and subsequent analyses. The precise TGF-β activity contained in MDA231-sEVs used in each experiment is indicated in correspondent figures and figure legends. As expected, treatment with rhTGF-β1 induced a dose-dependent increase in TGF-β/SMAD3 signaling activity in MDA231 cells, reaching a maximum induction at 2 ng/mL rhTGF-β1 (Fig. 2d). Surprisingly, however, MDA231-sEV treatment exceeded the maximum induction obtained with the rhTGF-β1 at 10 ng/mL by about 1.8-fold (rhTGF-β: ~900-fold; sEVs: ~1600-fold) (Fig. 2d). Although to a lower extent, a similar pattern was also observed by comparing phosphorylated (p)SMAD2 levels induced by rhTGF-β1 or MDA231-sEVs in MDA231 cells (Fig. 2e). Strikingly, these results were even more pronounced in the less aggressive MCF7 cells. Compared with rhTGF-β1-treated cells, MDA231-sEV treatment induced an approximately 3-fold increase in TGF-β/SMAD3 signaling activity (rhTGF-β: ~5-fold; sEVs: ~15-fold) (Fig. 2f). Similarly, pSMAD2 levels in MCF7 cells treated with MDA231-sEVs were higher than in cells treated with rhTGF-β1 (Fig. 2g).

To confirm the specificity of observed effects, we challenged cancer cells with TGF-β signaling inhibitors. Whereas cancer cells infected with control adenovirus showed increased TGF-β/SMAD3 signaling activity in response to both rhTGF-β1 and MDA231-sEVs, this effect was dramatically impaired in SMAD7-overexpressing cancer cells (Fig. 2h-i and Supplementary Fig. 4c). Treatment with SB431542 also reduced rhTGF-β1- and MDA231-sEV-induced TGF-β/SMAD3 signaling activity in cancer cells (Fig. 2j, k). Similarly, the challenge of MDA231 (Fig. 2l) and MCF7 cells (Fig. 2m) with SB431542 impaired SMAD2 phosphorylation in response to rhTGF-β1 or MDA231-sEVs. These results demonstrate that sEVs not only activate but are able to amplify the TGF-β signaling activity in recipient cells.

### Rab27a knockdown reduces sEV secretion and decreases the TGF-β signaling activity in breast cancer cells in vitro

**Fig. 3:**
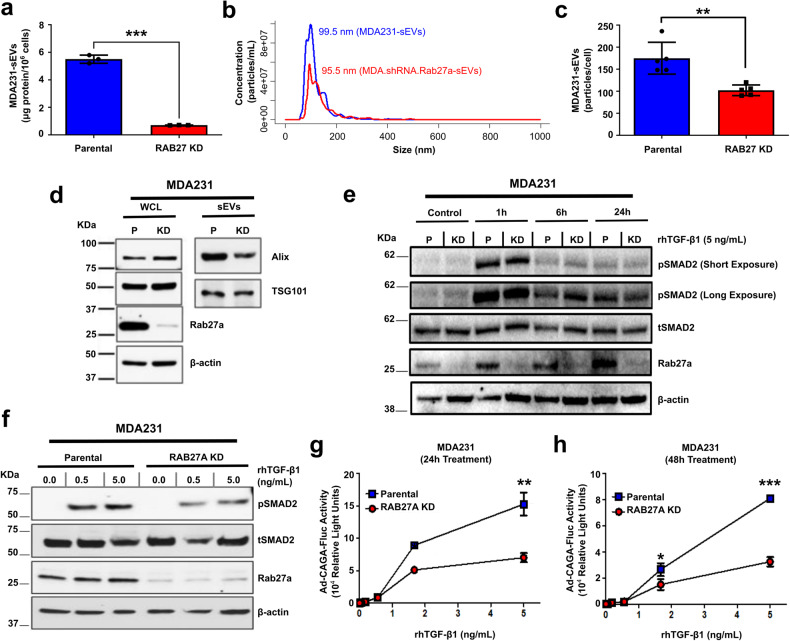
Rab27a knockdown decreases sEV secretion and inhibits the TGF-β signaling activity in breast cancer cells in vitro. sEVs were isolated from parental (P) MDA231 and MDA.Rab27a.shRNA (RAB27A knockdown (KD)) cell culture conditioned medium. **a** sEV secretion was quantified by BCA assay. **b**, **c** Particle size distribution and concentration evaluated by NTA. **d** Alix and TSG101 expression assessed by western blot in (P) and (KD) whole cell lysates (WCL) and sEVs. **e**, **f** pSMAD2 levels evaluated in (P) and (KD) cells treated (**e**) with 2 ng/mL rhTGF-β1 (increasing time intervals) or (**f**) with indicated rhTGF-β1 concentrations (8 h). Total (t)SMAD2 and β-Actin were used as loading controls. **g**, **h** The TGF-β/SMAD3 signaling reporter (Ad-CAGA-Fluc) activity was quantified in parental and RAB27A KD cells treated ± rhTGF-β1 for (**g**) 24 h or (**h**) 48 h. Results normalized by Gaussia luciferase (Ad-CMV-Gluc) activity. Results represent mean ± SD (*n* ≥ 3). Unpaired Student’s t-test used for comparison. **p < 0.01, ***p < 0.001

To evaluate the cellular relevance of sEVs regarding TGF-β signaling, we established a stable Rab27a knockdown (KD) in MDA231 cells (MDA.shRNA.Rab27a) as Rab27a KD impairs sEV secretion.^24^ Compared with parental cells, MDA.shRNA.Rab27a cells exhibited reduced Rab27a (Supplementary Fig. 5a) and secreted less sEVs as evaluated by total sEV protein, nanoparticle tracking analysis (NTA), and expression of sEV molecular markers (Fig. 3a–d).

Next, we analyzed the impact on TGF-β signaling activity associated with reduced sEV secretion in MDA.shRNA.Rab27a cells. Parental and Rab27a KD cells were treated with rhTGF-β1 and pSMAD2 levels were evaluated. Rab27a KD did not significantly impact the initial activation of the TGF-β signaling since pSMAD2 levels were similarly elevated in both parental and Rab27a KD cells treated with rhTGF-β1 for 1 h (Fig. 3e). However, Rab27a KD cells failed to sustain equivalent pSMAD2 levels 24 h after rhTGF-β1 treatment in comparison with parental cells (Fig. 3e). In agreement with these results, MDA.shRNA.Rab27a cells treated with rhTGF-β1 at increasing concentrations also showed decreased pSMAD2 levels in comparison with parental cells (Fig. 3f). Whereas parental MDA231 cells sustained high TGF-β-induced SMAD3 transcriptional activity for up to 48 h after rhTGF-β1 treatment, a significant reduction was observed in Rab27a KD cells (Fig. 3g, h). Rab27a siRNA transfection further validated these results (Supplementary Fig. 5b, c). These observations indicate that high sEV secretion is required to sustain elevated levels of TGF-β signaling activity in highly-metastatic MDA231 cells.

### TGF-β signaling is inhibited by DMA, Heparin and PNP-Xyl in vitro

**Fig. 4:**
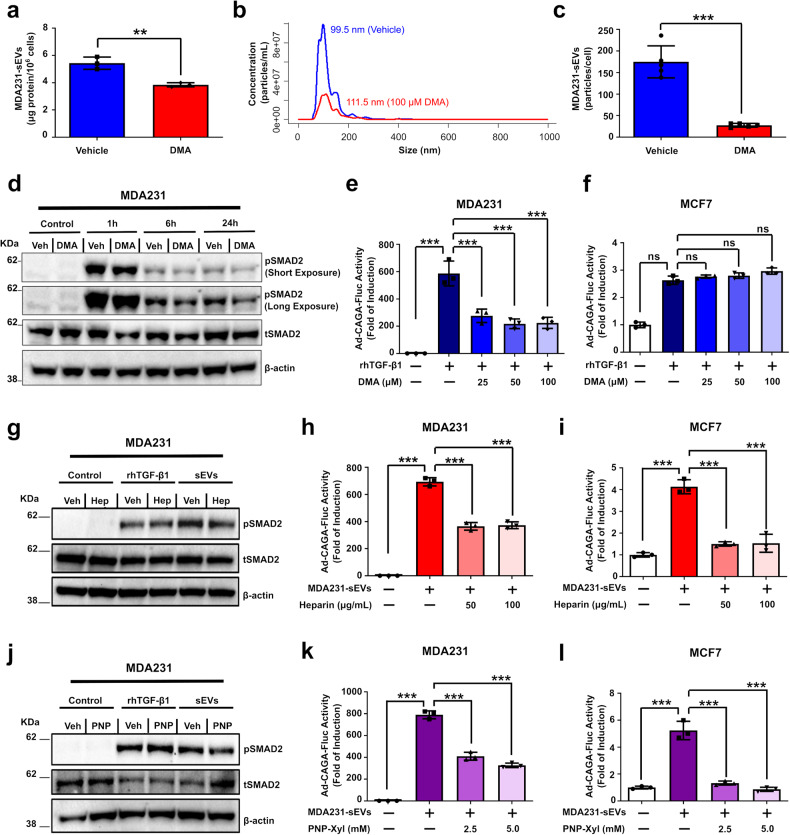
TGF-β signaling is inhibited by DMA, Heparin and PNP-Xyl in vitro. **a**–**d** sEVs were isolated from MDA231 cell culture conditioned medium after treatment ± 100 µM DMA (2 h). **a** sEV secretion quantified by BCA assay. **b**, **c** Particle size distribution and concentration evaluated by NTA. **d** pSMAD2 levels were assessed by western blot in MDA231 cells treated ± DMA (100 µM) and rhTGF-β1 (5 ng/mL). Total (t)SMAD2 and β-actin were used as loading controls. **e**, **f** TGF-β/SMAD3 signaling reporter (Ad-CAGA-Fluc) activity quantified in (**e**) MDA231 and (**g**) MCF7 cells treated ± rhTGF-β1 (5 ng/mL) ± DMA (24 h). Luciferase assay results normalized by Gaussia luciferase (Ad-CMV-Gluc) activity. **g** pSMAD2 levels assessed in MDA231 cells treated with rhTGF-β1 or MDA231-sEVs after treatment ± heparin. **h**, **i** TGF-β/SMAD3 signaling activity quantified in (**h**) MDA231 and (**i**) MCF7 cells treated with MDA231-sEVs ± heparin as in (**e**, **f**). **j** pSMAD2 levels assessed in MDA231 cells treated with rhTGF-β1 or MDA231-sEVs after treatment ± PNP-Xyl as in (**d**, **g**). **k**, **l** TGF-β/SMAD3 signaling activity quantified in (**k**) MDA231 and (**l**) MCF7 cells treated with MDA231-sEVs after treatment ± PNP-Xyl (24 h). Results represent mean ± SD (*n* ≥ 3). Unpaired Student’s *t*-test was used to analyze data in (**a**, **c**). One-way ANOVA test followed by Dunn’s Multiple Comparison test was used to analyze data in (**e**–**f**, **h**–**i**, **k**–**l)**. ns: statistically non-significant, **p < 0.01, ***p < 0.001

Since the TGF-β signaling activity was inhibited in cells with impaired sEV secretion by Rab27a KD, we reasoned that this could be recapitulated by pharmacologically targeting sEV secretion. Indeed, MDA231 cells treated with DMA (100 µM) showed reduced sEV secretion (Fig. 4a–c and Supplementary Fig. 6). Interestingly, whereas DMA treatment did not alter pSMAD2 levels in MDA231 cells treated with rhTGF-β1 for 1 h, decreased pSMAD2 levels were seen in cells treated for 6–24h (Fig. 4d). In agreement with the critical role of sEVs in the TGF-β signaling pathway, DMA treatment efficiently inhibited the TGF-β/SMAD3 signaling activity in MDA231 cells in a concentration-dependent manner (Fig. 4e). Of note, a similar effect was not observed in MCF7 cells (Fig. 4f). These results reinforce the hypothesis that sEVs are critical for the TGF-β signaling hyperactivation in highly-metastatic cancer cells but have minor relevance for near-normal TGF-β signaling levels in poorly-metastatic cells.

We next treated breast cancer cells with heparin to inhibit sEV uptake before exogenously adding sEVs to the cell culture. Heparin treatment did not impact cells treated with rhTGF-β1 for 1 h, but reduced pSMAD2 levels were observed in cell cultures challenged with heparin and treated with MDA231-sEVs for the same period (Fig. 4g). Moreover, heparin decreased the TGF-β/SMAD3 signaling activity otherwise induced by exogenous MDA231-sEVs in MDA231 and MCF7 cells 24 h after treatment (Fig. 4h, i). Similarly, when we used PNP-Xyl to impair sEV uptake, drug treatment decreased the pSMAD2 expression in cell cultures treated with MDA231-sEVs for 1 h but did not affect cells treated with rhTGF-β1 for the same period (Fig. 4j). Further, PNP-Xyl impaired TGF-β/SMAD3 signaling activity induced by MDA231-sEVs in MDA231 and MCF7 cells (Fig. 4k, l). These results validate the major role played by sEVs in TGF-β’s biology. In highly-metastatic MDA231 cells, inherently elevated TGF-β signaling activity is decreased by genetically or pharmacologically targeting sEV secretion. In poorly-metastatic MCF7 cells, however, inhibiting sEV secretion does not impact the TGF-β signaling activity.

### sEVs induce cancer cell EMT, migration and invasion in vitro

**Fig. 5:**
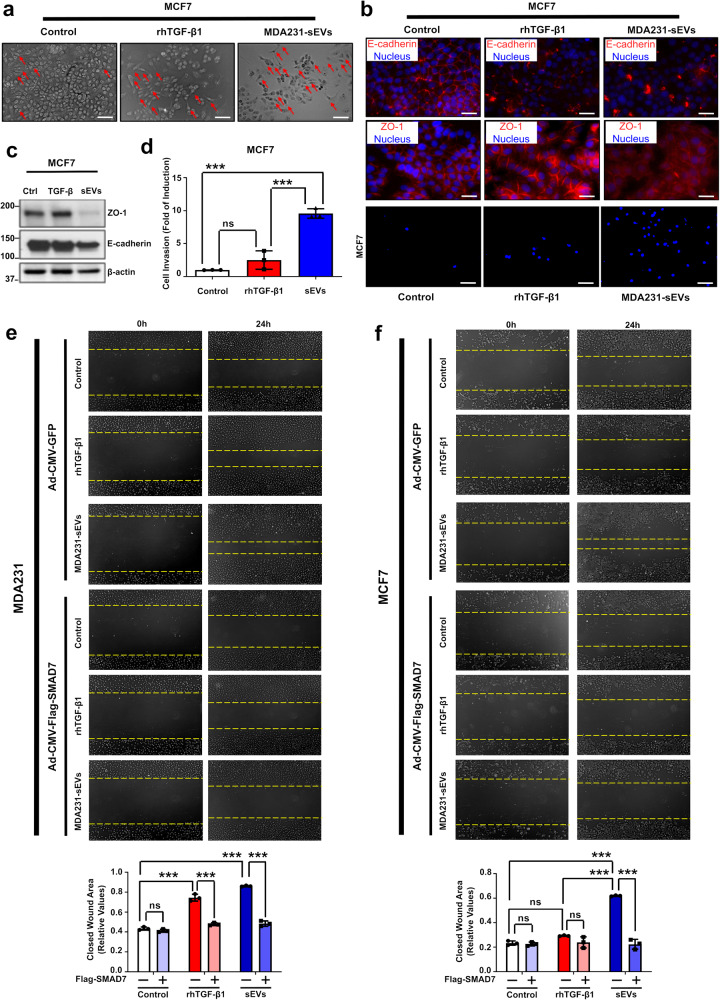
sEVs induce cancer cell EMT, migration, and invasion in vitro. **a** MCF7 cell morphology evaluated by phase contrast after treatment ± rhTGF-β1 or MDA231-sEVs (5 days). Red arrows indicate elongated cells. Scale bar is equal to 100 µm. **b** ZO-1 and E-cadherin localization analyzed by immunofluorescence staining after single treatment ± rhTGF-β1 or MDA231-sEVs (5 days). Scale bar is equal to 50 µm (**c**) ZO-1 and E-cadherin expression assessed by western blot in MCF7 cells treated once ± rhTGF-β1 or MDA231-sEVs (5 days). β-actin used as loading control. **d** MCF7 cell invasion quantified in cells seeded in Matrigel-coated transwell inserts and treated ± rhTGF-β1 or MDA231-sEVs (48 h). Scale bar is equal to 100 µm. **e**, **f** Cell migration quantified by wound healing assay in (**e**) MDA231 and (**f**) MCF7 cell cultures infected ± Ad-CMV-Flag-SMAD7 and treated as indicated. Ad-CMV-GFP: control adenovirus. Results represent mean ± SD (*n* ≥ 3). One-Way ANOVA followed by Dunn’s Multiple Comparison Test. *p < 0.05, **p < 0.01, ***p < 0.001, ns: statistically non-significant

TGF-β is a master EMT inducer.^10^ To investigate if sEVs could also amplify this effect in breast cancer cells, we treated MCF7 cells with sEVs containing TGF-β activity and used matching rhTGF-β1 concentration for comparison. Whereas untreated MCF7 cell cultures typically show cobblestone-like morphology, an elongated morphology was triggered by rhTGF-β1 or MDA231-sEV treatment (Fig. 5a). In addition, while untreated controls showed strong ZO-1 and E-cadherin staining localized at the plasma membrane, these epithelial markers were re-localized towards the cytoplasm in MCF7 cells treated with rhTGF-β1 or MDA231-sEVs (Fig. 5b). Moreover, MDA231-sEVs further downregulated ZO-1 and E-cadherin protein expression (Fig. 5c).

Highly-metastatic MDA231 cells treated with rhTGF-β1 or MDA231-sEVs exhibited increased migration (Supplementary Fig. 7a). Moreover, while MCF7 cells showed insignificant migratory response to rhTGF-β1, MDA231-sEVs potently enhanced the migration of these weakly metastatic cells (Supplementary Fig. 7b). Similarly, MDA231-sEV treatment increased the invasion of MCF7 and MDA231 cells in comparison with rhTGF-β1 treatment (Fig. 5d and Supplementary Fig. 7c).

Yet, sEVs are expected to transport multiple types of cargo. To ascertain the proportional contribution of the TGF-β signaling to sEV-mediated effects, we used genetic and pharmacological inhibitors. As expected, while MDA231 cells infected with Ad-CMV-GFP (control) adenovirus showed strong migration in response to rhTGF-β1 treatment, SMAD7 overexpression by infection with Ad-CMV-Flag-SMAD7 adenovirus reduced this effect (Fig. 5e). Similarly, SMAD7 overexpression virtually abolished MDA231 cell migration induced by MDA231-sEVs (Fig. 5e). Likewise, SMAD7 overexpression dramatically impaired the migration of MCF7 cells otherwise triggered by MDA231-sEVs in cells infected with control adenovirus (Fig. 5f). Reinforcing the relevance of TGF-β signaling, treatment with SB431542 also efficiently decreased cancer cell migration (Supplementary Fig. 8a, b) in response to either rhTGF-β1 or MDA231-sEVs. These results were further validated by quantifying cell migration in transwell inserts since SB431542 treatment significantly impaired cancer cell migration (Supplementary Fig. 8c–f).

To further understand the cellular role of sEV-induced TGF-β signaling in cancer cell aggressiveness, the migration of Rab27a KD breast cancer cells was analyzed in transwell inserts. Compared with parental cells, MDA.shRNA.Rab27a cells showed reduced migration when treated with rhTGF-β1 (Supplementary Fig. 9a). Similar results were also obtained with MDA231 cells transfected with Rab27a siRNA (Supplementary Fig. 9b). Further validating these results, DMA treatment significantly impaired MDA231 cell migration in transwell inserts (Supplementary Fig. 9c). Additionally, treatment with PNP-Xyl or heparin also impaired the migration of MDA231-sEV-treated breast cancer cells (Supplementary Fig. 9d–f). Taken together, these results demonstrate an important role for sEVs in inducing cancer cell pro-metastatic phenotype in vitro, reinforcing the activity of sEVs in mediating the transference of an aggressive phenotype to poorly-invasive breast cancer cells. Moreover, TGF-β signaling activation significantly contributes to these biological effects as TGF-β signaling inhibitors potently impaired sEV-driven cancer cell aggressiveness.

### sEVs hyperactivate TGF-β signaling and enhance breast cancer progression in vivo

**Fig. 6:**
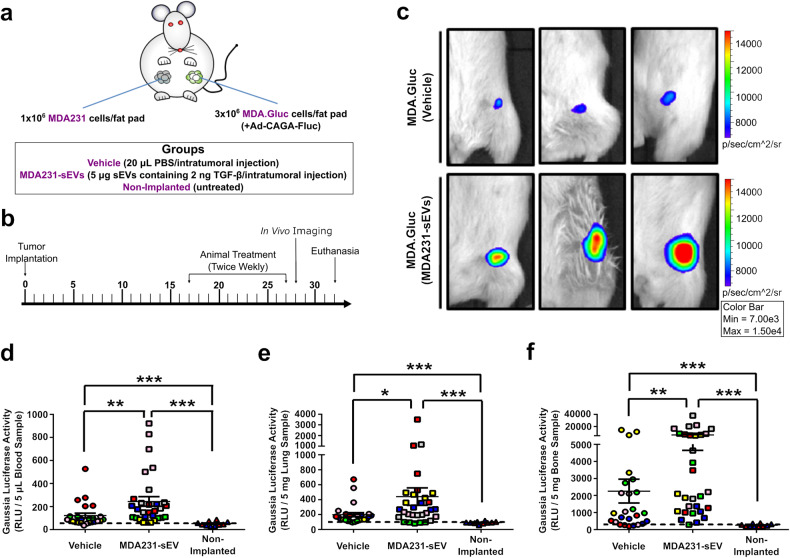
sEVs increase TGF-β signaling and enhance MDA231 breast cancer progression in vivo. **a** Illustration of breast cancer mouse model showing the orthotopic implantation of MDA231 cells. Unlabeled MDA231 and Gaussia luciferase-labeled MDA.Gluc cells were implanted contralaterally. **b** Experiment timeline for (**a**). **c** TGF-β/SMAD3 signaling reporter (Ad-CAGA-Fluc) activity quantified in MDA.Gluc tumors (3 animals/group) by IVIS. **d**–**f** Gaussia luciferase activity quantified by luciferase assay in (**d**) blood, (**e**) lung, and (**f**) bone samples (5–6 mice/group). Animals are color-coded. Black dashed lines indicate the background activity for the Gaussia luciferase quantified in samples from non-implanted mice (*n* = 2). Results represent mean ± SEM. One-Way ANOVA followed by Tukey’s Multiple Comparison Test. *p < 0.05, **p < 0.01, ***p < 0.001

To evaluate if the amplification of TGF-β signaling induced by sEVs in vitro could be reproduced in vivo, a breast cancer xenograft mouse model^39,40^ was used. Unlabeled MDA231 cells and Gaussia luciferase-labeled MDA231 (MDA.Gluc) cells were orthotopically and contralaterally implanted and investigated in vivo as breast cancer cells highly responsive to TGF-β (Fig. 6a, b). MDA231-sEVs were intratumorally injected in MDA.Gluc tumors infected with Ad-CAGA-Fluc adenovirus and the TGF-β/SMAD3 reporter (Ad-CAGA-Fluc) signaling activity was evaluated in tumors by In Vivo Imaging System (IVIS) (Fig. 6c and Supplementary Fig. 10a). Compared with vehicle-injected tumors, treatment with MDA231-sEVs did not impact tumor growth (Supplementary Fig. 10b–g). Remarkably, however, the bioluminescence quantified in MDA231-sEV-treated tumors (12.49 ± 2.41 × 10^4^ p/s) was approximately 3.2-fold higher than in vehicle-treated tumors (3.8 ± 0.45 × 10^4^ p/s) (Fig. 6c and Supplementary Fig. 10a), confirming sEV-led TGF-β signaling hyperactivation in vivo.

To investigate if exogenous sEVs could impact cancer progression, tissues and distant organs from mice implanted with MDA.Gluc cells were then analyzed ex vivo. Tissues and organs harvested from age-matched mice not implanted with MDA.Gluc cells were used as negative controls and established a baseline for the Gaussia luciferase activity at each site. Importantly, the elevated activity shown by Gaussia luciferase combined with the high sensitivity of the ex vivo luciferase assay allows the detection and quantification of Gaussia luciferase-labeled cells at single-cell level (Supplementary Fig. 8d).^6^ CTCs were detected in all tumor-implanted animals, while MDA231-sEV treatment further increased the number of CTCs (Fig. 6d and Supplementary Fig. 10h). Furthermore, sEV-treated mice also showed elevated metastatic levels in lungs (Fig. 6e and Supplementary figure 10i) and bones (Fig. 6f and Supplementary Fig. 10j), while alterations were not observed in liver (Supplementary Fig. 10k, l) or brain (Supplementary Fig. 10m, n) samples. Additionally, a statistically non-significant trend in unlabeled MDA231 tumors (Supplementary Fig. 10o, p) suggested elevated tumor-self-seeding in MDA231-sEV-treated mice, an outcome characterized by the return of CTCs from the circulation to the primary tumor.^41^

**Fig. 7:**
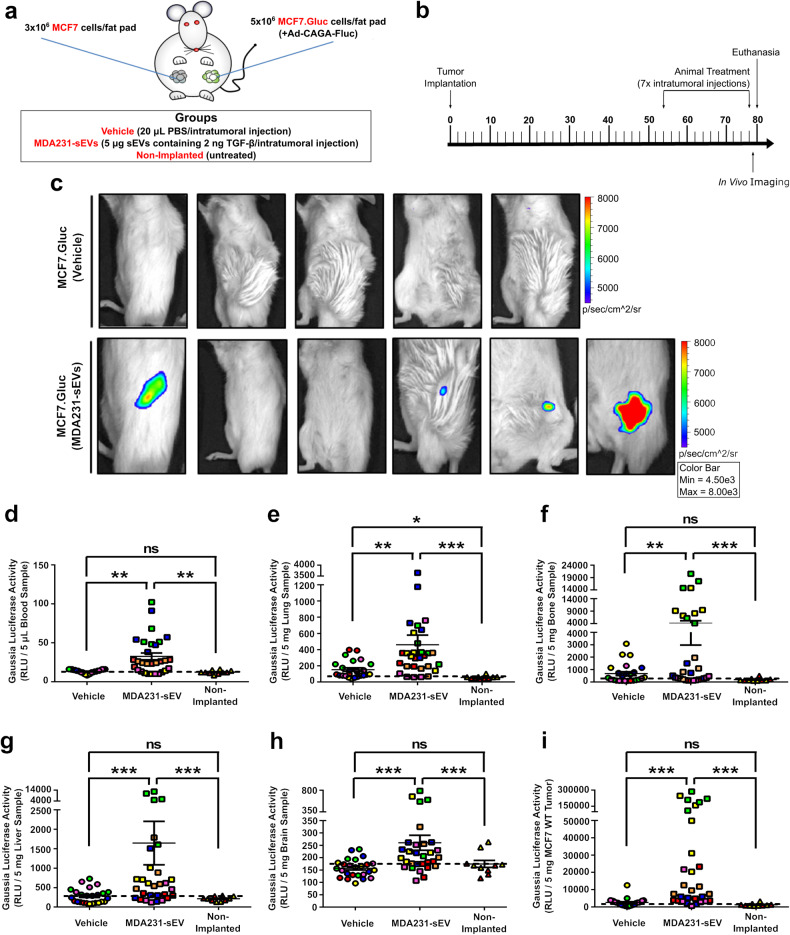
sEVs increase TGF-β signaling and enable MCF7 breast cancer progression in vivo. **a** Illustration of MCF7 breast cancer mouse model. Unlabeled MCF7 and Gaussia luciferase-labeled MCF7.Gluc cells were implanted contralaterally. **b** Experiment timeline for (**a**). **c** TGF-β/SMAD3 signaling activity was quantified in MCF7.Gluc tumors (5-6 mice/group). **d**–**i** Gaussia luciferase activity quantified by luciferase assay in (**d**) blood, (**e**) lung, (**f**) bone, (**g**) liver, (**h**) brain, and (**i**) unlabeled MCF7 tumor samples (5–6 animals/group). Animals are color-coded. Black dashed lines indicate the background activity for the Gaussia luciferase quantified in samples from non-implanted mice (*n* = 2). Results represent mean ± SEM. One-Way ANOVA followed by Tukey’s Multiple Comparison Test. *p < 0.05, **p < 0.01, ***p < 0.001, ns: statistically non-significant

Next, we investigated if a similar effect could be observed in a model of poorly-metastatic breast cancer cells. Unlabeled MCF7 cells and Gaussia luciferase-labeled MCF7 (MCF7.Gluc) cells were contralaterally implanted and analyzed in vivo as breast cancer cells weakly responsive to TGF-β (Fig. 7a, b). As above, vehicle- and MDA231-sEV-treated tumors exhibited similar tumor growth (Supplementary Fig. 11a–f). Nonetheless, while we failed to detect bioluminescence in vehicle-treated MCF7.Gluc tumors, significant TGF-β/SMAD3 signaling activity was observed in four of six MCF7.Gluc tumors injected with MDA231-sEVs (3.327 ± 2.160 × 10^4^ p/s) (Fig. 7c). Further corroborating our hypothesis, CTCs were almost absent in vehicle-treated mice, but MDA231-sEV treatment effectively induced the intravasation of MCF7.Gluc cells as observed by the higher Gaussia luciferase activity in blood samples of sEV-treated mice (Fig. 7d and Supplementary Fig. 11g). Remarkably, MDA231-sEV-treated mice showed increased metastasis in lungs (Fig. 7e and Supplementary Fig. 11h), bones (Fig. 7f and Supplementary Fig. 11i), liver (Fig. 7g and Supplementary Fig. 11j), and brain (Fig. 7h and Supplementary Fig. 11k) when compared with vehicle-injected animals. Additionally, MDA231-sEV treatment significantly increased tumor self-seeding (Fig. 7i and Supplementary Fig. 11l). Collectively, these results confirm the contribution of sEVs to TGF-β signaling pathway hyperactivation by recapitulating this pattern in breast cancer cells in vivo.

### In vivo TGF-β signaling activity and breast cancer progression are impaired by combined treatment with DMA and SB431542 at suboptimal doses

We next questioned if targeting the TGF-β signaling pathway could be improved by combining suboptimal doses of a specific TGF-β inhibitor with drugs targeting sEV trafficking. DMA showed the strongest effects and the higher potential to be used at low doses, minimizing the risk of adverse effects in vivo. Suboptimal concentrations for DMA (Fig. 4e) and SB431542 (Supplementary Fig. 12a) were determined in vitro. Combined treatment with SB431542 and DMA at low concentrations potently inhibited the TGF-β/SMAD3 signaling activity (Supplementary Fig. 12b) and the migration (Supplementary Fig. 12c) of MDA231 cells in vitro.

**Fig. 8:**
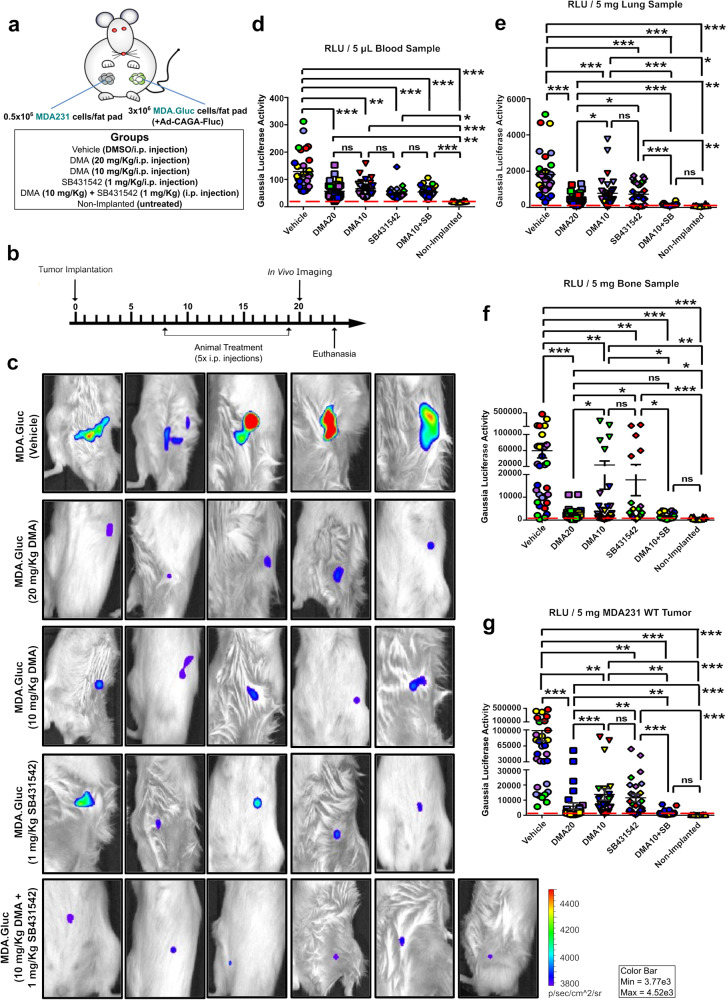
In vivo TGF-β signaling activity and breast cancer progression are impaired by combined treatment with DMA and SB431542 at suboptimal doses. **a** Illustration of breast cancer mouse model showing MDA231 orthotopic implantation. Unlabeled MDA231 and Gaussia luciferase-labeled MDA.Gluc cells were implanted contralaterally. **b** Experiment timeline for (**a**). **c** TGF-β/SMAD3 signaling reporter (Ad-CAGA-Fluc) activity quantified in MDA.Gluc tumors (5–6 animals/group). **d**–**g** Gaussia luciferase activity quantified by luciferase assay in (**d**) blood, (**e**) lung, (**f**) bone, and (**g**) unlabeled MDA231 tumor samples (6 animals/group). Animals are color-coded. Red dashed lines indicate the background activity for the Gaussia luciferase quantified in samples from non-implanted mice (*n* = 2). One-Way ANOVA followed by Tukey’s Multiple Comparison Test. Unpaired Student’s *t*-test used for comparison of drug-treated groups. *p < 0.05, **p < 0.01, ***p < 0.001, ns: statistically non-significant

To investigate if this effect could be translated as anti-metastatic therapy, we first analyzed the TGF-β signaling activity in breast cancer cells in vivo. Based on previous findings,^27,42^ mice were treated with 20 mg/Kg or 10 mg/Kg DMA as optimal and suboptimal doses, respectively. Similarly, 1 mg/Kg SB431542 was determined as the suboptimal dose to be used here considering previous reports.^43,44^ To quantify the TGF-β signaling activity in breast cancer cells in vivo, MDA231 and MDA.Gluc cells were implanted in NOD-SCID mice (Fig. 8a, b). Drug treatment did not affect tumor growth (Supplementary Fig. 13a–f). Compared with tumors from vehicle-treated mice, the TGF-β/SMAD3 signaling activity in MDA.Gluc tumors was reduced in all drug-treated groups (Fig. 8c and Supplementary Fig. 13g). Furthermore, combination treatment with DMA and SB431542 at low doses further decreased the TGF-β/SMAD3 signaling reporter (Ad-CAGA-Fluc) activity in MDA.Gluc tumors compared with tumors from animals treated either with 10 mg/Kg DMA or 1 mg/Kg SB431542 singly (Fig. 8c and Supplementary Fig. 13g).

Next, we evaluated the impact caused by DMA and SB431542 in breast cancer progression. Treatment with individual drugs significantly decreased CTCs (Fig. 8d and Supplementary Fig. 14a), metastasis in lungs (Fig. 8e and Supplementary Fig. 14b) and bones (Fig. 8f and Supplementary Fig. 14c), and tumor self-seeding (Fig. 8g and Supplementary Fig. 14d). Remarkably, combined treatment almost abolished the Gaussia luciferase activity in lungs (Fig. 8e and Supplementary figure 14b), bones (Fig. 8f and Supplementary Fig. 14c), and unlabeled tumors (Fig. 8g and Supplementary Fig. 14d), reducing it to background levels comparable to samples from non-implanted mice. Noteworthy, whereas a combined effect was not observed in liver (Supplementary Fig. 14e, f) and brain (Supplementary Fig. 14g, h) samples, treatment with DMA and/or SB431542 has also significantly reduced metastasis in these sites.

**Fig. 9:**
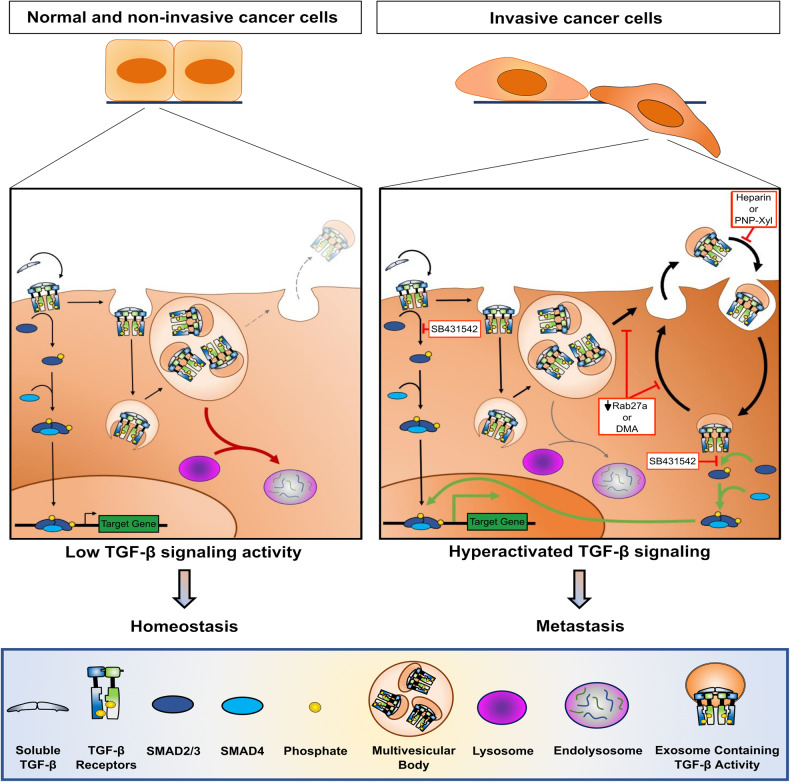
Working model. Normal and non-invasive breast cancer cells show low TGF-β signaling activity required for homeostasis. The TGF-β signaling in these cells activated downstream ligand-receptor binding is shortly terminated following endocytosis of the ligand-receptor complex and its degradation in endolysosomes. However, invasive breast cancer cells exhibit TGF-β signaling hyperactivation that induces metastasis. In these cells, degradation of ligand-receptor complex is potently antagonized by increased exosome secretion. Uptake of exosomes containing TGF-β activity drives TGF-β signaling hyperactivation which is prolonged in highly-metastatic breast cancer cells by sustaining cycles of exosome secretion and uptake. Targeting exosome secretion by reducing Rab27a levels or DMA treatment reduces TGF-β signaling levels in invasive breast cancer cells, an effect similarly observed by targeting exosome uptake via heparin or PNP-Xyl treatment. Simultaneously targeting exosome trafficking (DMA, heparin, or PNP-Xyl) and TβRI kinase activity (SB431542) is a novel therapeutic strategy to normalize TGF-β signaling levels and efficiently impair cancer progression and metastasis. Here, fading colors and arrow thickness represent the intensity of a given step. Red arrows indicate TGF-β signaling termination. Green arrows highlight TGF-β signaling hyperactivation. Red and white boxes show strategies to inhibit the TGF-β signaling

Whereas non-invasive breast cancer cells show low TGF-β signaling activity, this molecular pathway is hyperactivated in invasive counterparts due to increased sEV trafficking. Thus, characterizing this process allows targeting sEV secretion/uptake to normalize TGF-β signaling levels and inhibit metastasis. Moreover, if a systemic effect cannot be discarded, our results strongly suggest that a combined approach with DMA and SB431542 at low doses can effectively impair breast cancer metastasis by further improving the inhibition of the TGF-β signaling in primary tumors (Fig. 9).

## Discussion

TGF-β and its signaling are critical drivers for metastasis and negatively associated with cancer patient survival.^4,6^ Consequently, numerous clinical trials have been developed targeting TGF-β signaling for cancer treatment. It is unfortunate that none of these have so far succeeded clinically even though this is one of the most important targets.^10^ This could be the result of a potential knowledge gap between the signaling mechanism we understand and what is critical in reality. Here, we show that sEVs are critical players driving the TGF-β signaling hyperactivation in highly-invasive breast cancer cells. Moreover, sEVs also induced an aggressive behavior in otherwise poorly-invasive breast cancer cells, enabling their progression towards metastasis and tumor self-seeding. By hindering sEV trafficking, we successfully inhibited TGF-β signaling in vitro and in vivo, reducing cancer cell invasive phenotype, and ameliorating cancer progression. Finally, we established a new and effective therapeutic approach to decrease metastasis and tumor self-seeding by combining SB431542 and DMA at low, suboptimal doses, which simultaneously target TGF-β signaling and sEV trafficking.

Due to a growing effort to characterize EV’s biology, emerging EV-related signatures have been recently reported, reinforcing the relationship between elevated EVs’ activity and cancer progression.^31–35^ In this study, we generated a score system for individual breast cancer patients to obtain molecular signatures representing the average expression of genes involved in the biogenesis of exosomes, a type of EV. Whereas most studies focused on alterations in EV cargos, we intentionally mined information on intracellular molecules known to regulate exosome biogenesis, which enabled a broader investigation on interactions between different biological processes during cancer progression. Analysis of two independent cohorts revealed that increased scores for exosome secretion are well correlated with reduced survival of breast cancer patients, particularly regarding progression to metastasis. Further, the positive association between the scores for exosome secretion, TGF-β signaling, and EMT suggests a cooperation between sEV secretion and TGF-β signaling to drive cancer progression, which was further confirmed in vitro and in vivo. In combination with further research on the intracellular effectors controlling EV biogenesis, the association between alterations in EV secretion and molecular signatures for other EMT-related pathways^45,46^ can also be explored. A deeper understanding of the crosstalk between multiple signaling pathways may help us to more accurately determine molecular drivers promoting disease progression.

Whereas TGF-β-induced cancer progression is a well-established concept, it is reported that non-invasive cancer cells exhibit a weaker responsiveness to TGF-β that is insufficient to drive metastasis.^10^ Further, low TGF-β signaling levels are often explained by prompt signaling termination following endocytosis and proteasome/lysosome degradation of the TGF-β receptors complexes.^47,48^ In this study, we show that hyperactivation of TGF-β signaling in invasive cancer cells is caused by accelerated secretion and uptake of sEVs containing TGF-β activity. This vicious cycle generated by enhanced sEV trafficking not only compensates but exceeds degradation-mediated TGF-β signaling termination. Furthermore, this imbalance between endolysosomal degradation and increased sEV trafficking (secretion and uptake) enables invasive cancer cells to sustain elevated TGF-β signaling levels for prolonged periods. Amplified and sustained TGF-β signaling as a result of increased sEV trafficking are required for metastasis, which has been reproduced in our study in non-invasive breast cancer cells by providing sEVs containing TGF-β activity.

By activating the TGF-β signaling, sEVs induce EMT and cancer progression.^17^ This may be partially explained by the transfer of TGF-β and its receptors to recipient cells via sEVs.^18,49,50^ However, we demonstrate that sEVs not only activate, but also underlie the amplification of TGF-β signaling levels even shortly after treatment, implying that sEVs may transfer TGF-β and TGF-β receptors as an activated ligand-receptor complex. Moreover, since sEV-mediated TGF-β signaling amplification enabled a metastatic behavior in poorly-invasive breast cancer cells, this implies that sEVs could mediate phenocopy within a heterogeneous tumor mass. In addition to transporting mutated oncogenic cargo to non-malignant cells,^51,52^ we show in this work that sEVs can functionally transfer and amplify a pro-metastatic signaling pathway into recipient cancer cells. Future studies must establish the precise contribution of sEV-mediated phenocopy to metastasis by analyzing the activity of these EVs in malignant tumors composed of invasive and non-invasive cancer cells in different ratios. Also, the transference of an aggressive behavior that is mediated by sEVs might also involve the crosstalk between cancer cells and non-cancer cells within the tumor microenvironment (TME).^53^ EVs are known to activate the TGF-β signaling in non-cancer cells, inducing fibroblast activation^18,19,30^ and immunosuppression.^20,54^ Therefore, cancer cell-sEVs may not only activate, but also accelerate the co-option of a pro-metastatic TME by enhancing the activity of TGF-β and other growth factors by targeting non-cancer cells. Moreover, even early-stage tumors containing few aggressive cancer cells, could show non-cancer cells that are able to secrete EVs, thus, driving a similar pro-metastatic phenotype. As previously described, sEVs secreted by non-cancer cells can promote TGF-β signaling activation and EMT-related effects in cancer cells.^17,49^ Nonetheless, additional research is still needed to conclusively characterize the role of sEVs secreted by non-cancer cells, particularly regarding their ability to drive metastasis by hyperactivating molecular pathways in cancer cells.

Since we have shown a critical role for sEVs in enhancing TGF-β-induced cancer progression, a therapeutic approach to counteract this effect would involve inhibiting sEV trafficking. Pharmacological targeting of EV trafficking has been shown to reduce EV secretion in vivo.^27,42,55^ However, the anti-metastatic activity of these drugs remains unclear. Here, in vivo administration of DMA at high dose potently inhibited the TGF-β signaling in a MDA231 breast cancer model, decreasing CTCs, metastasis, and tumor self-seeding without altering cancer growth. Further, treatment with the specific TβRI kinase inhibitor SB431542 was potentiated by combination with DMA at low, suboptimal doses, to efficiently inhibit the TGF-β signaling in vitro and in vivo in an orthotopic xenograft breast cancer model. The powerful effect obtained with this combined therapy not only reduced, but almost abolished tumor self-seeding and metastasis in the lungs and bones. This approach was designed to be more than a new method to inhibit TGF-β-induced metastasis as it may have implications for multiple cancer hallmarks. Whereas our work highlighted a major relevance for TGF-β signaling in breast cancer progression, impairing sEV trafficking might help to simultaneously target multiple pro-metastatic effectors transported by EVs. Also, the rationale of targeting sEVs must not be limited to an on-off effect of signaling pathways. Rather, the potential therapy proposed in this study aimed at normalizing the TGF-β signaling by reducing the activity of a pro-metastatic signaling pathway from an hyperactivated state, seen in highly-invasive breast cancer cells towards near-normal levels seen in poorly-metastatic breast cancer cells. Such a novel strategy could have potential clinical relevance to slow down breast cancer progression.

Although our approach aims to normalize an otherwise hyperactivated signaling pathway, it is noteworthy that the translation of this low, suboptimal dosage, treatment strategy into clinics would benefit patients with reduced side-effects. Indeed, many cancer clinical trials for pharmacological inhibitors targeting the TGF-β signaling have reported side effects, as activation of this molecular pathway is required for normal tissue homeostasis.^10^ Additionally, whereas many studies have investigated the contribution of EVs to pathological conditions, their role in normal physiology must not be ignored,^21^ and therapies used to inhibit EV trafficking might also trigger adverse events. Our strategy was designed to overcome these limitations in immunocompromised mouse models by combining suboptimal doses of DMA and SB431542 while retaining their anti-metastatic activity. Yet, impacts on immune cells and the possibility of drug interactions may not be neglected, particularly when considering well-established differences in the metabolism of xenobiotics across species.

In conclusion, this work establishes a new role for EVs in driving breast cancer metastasis via hyperactivation of the TGF-β signaling in cancer cells, a previously poorly characterized phenomenon that may explain the failure in translating TGF-β signaling inhibitors from bench-to-bedside. Further, we demonstrate the feasibility to effectively suppress TGF-β-induced metastasis by combining DMA and SB431542 treatment at relative low doses. We envision that future work will build on our findings to improve the strategies currently used to inhibit TGF-β-induced cancer progression, particularly by exploring the potential clinical benefits of inhibitors targeting EV trafficking.

## Materials and methods

### Breast cancer cohorts and datasets

Human clinical breast cancer data downloaded from USCS Xena Browser (https://xenabrowser.net) were derived from the publicly available datasets TCGA BRCA^36^ and NKI-295 (ref. ^37^). Normalized RNA sequencing- or microarray-based gene expression data were obtained, matched with patient clinical data and used for further analyses.

### Gene expression and gene signature scores

The full list of genes analyzed in this study is available in the Supplementary information. Genes coding proteins involved in exosome biogenesis or secretion were manually curated from published papers and/or associated with these processes by the Gene Ontology Resource (http://geneontology.org/).

Exosome-related genes were categorized into four biological processes as described in the Supplementary information. Signature scores representing these biological processes were defined as the mean of the weighted gene expression (Eq. (1)). Previously described gene expression signatures relative to TGF-β responsive genes (TBRS)^56^ and EMT-related genes^38^ were used to investigate TGF-β signaling activity and EMT in human breast cancers. Missing genes were removed from the final gene expression signature when analyzing the microarray-based NKI-295 dataset. Genes matching more than one microarray probe in the NKI-295 dataset were merged and the expression of this gene was represented by the mean value of individual microarray probes.

1 $${S}_{m}=\frac{{\Sigma }_{i=1}^{N}({\omega }_{i}\times {\exp }_{i,m})}{N}$$

where ${\omega }_{i}$ represents the weighting factor. $N$ is the number of genes in a set of genes containing $i$, whereas ${\exp }_{i,m}$ is the expression of any gene $i$ in each sample $m$.

Data analysis was performed with R (version 3.6.3; https://www.r-project.org/). Kaplan-Meier survival curves were obtained with the R packages ‘survival’ (v3.1-12) and ‘survminer’ (v0.4.6). Correlation plots were obtained with R packages ‘ggstatsplot’ (v0.9.3) and ‘ggplot2’ (v0.4.0).

### Cell lines and cell culture

The human breast cancer cell lines MDA-MB-231 (MDA231) and MCF7 were purchased from American Type Culture Collection (ATCC). Genetically modified breast cancer cell lines (MDA.Gluc and MCF7.Gluc) were generated by transfection with Gaussian-luc cDNA construct to promote the constitutive expression of Gaussia luciferase in neomycin resistant cells. The stable Rab27a knockdown cell line (MDA.Rab27a.shRNA) was established by Rab27a shRNA lentivirus (sc-41834-v, Santa Cruz Biotechnology, Bio-Strategy Pty Limited, Australia) transduction using Polybrene® (sc-134220, Santa Cruz Biotechnology, Bio-Strategy Pty Limited, Australia) in puromycin resistant cells. Transient Rab27a knockdown in MDA231 cells was established by Rab27a siRNA (sc-41834, Santa Cruz Biotechnology, Bio-Strategy Pty Limited, Australia) transfection using Lipofectamine™ RNAiMAX Transfection Reagent (Invitrogen, Thermo Fisher Scientific, Australia PTY LTD). Successful knockdown was confirmed by western blot. All cell lines were cultured in Dulbecco’s modified Eagle’s medium (DMEM) (1965118, Gibco, Thermo Fisher Scientific, Australia PTY LTD) supplemented with 10% fetal calf serum (FCS) (HyCloneTM, GE Healthcare Life Sciences, USA), 10 µg/mL penicillin and 100 µg/mL streptomycin (Invitrogen, Thermo Fisher Scientific, Australia PTY LTD). Cell cultures were grown at 37 °C with 10% CO_2_ in a humidified atmosphere.

### DNA constructs and adenovirus production

The Ad-CAGA-Fluc, Ad-CMV-Gluc, Ad-CAGA-Gluc, Ad-BRE-Fluc, Ad-TCF-Fluc, Ad-CMV-GFP, and Ad-CMV-Flag-SMAD7 adenoviruses were produced and amplified as previously described.^57,58^ To quantify the TGF-β/SMAD3 signaling activity, *pCAGA-Fluc* DNA was initially cloned into *pENTR 1* *A* entry clone vector (Invitrogen, Thermo Fisher Scientific, Australia PTY LTD) to obtain *pENTR-CAGA-FLuc*. Next, attL-arrR (LR) recombination was performed with *pAd/PL-DEST* Destination vector (Invitrogen, Thermo Fisher Scientific, Australia PTY LTD) to obtain *pAdCAGA*_*12*_*-luc* Adenoviral plasmid. After digestion with *Pac I* to expose the inverted terminal repeats, the plasmid was transfected into the adenovirus producing 293 A cell line using Lipofectamine LTX transfection reagent (Invitrogen, Thermo Fisher Scientific, Australia PTY LTD). After most cells were lysed, nearly two weeks after transfection, floating cells were harvested, the adenovirus was amplified, titered and used to quantify the cellular response to TGF-β. Similar procedures were done to produce Ad-CMV-Gluc, Ad-CAGA-Gluc, Ad-BRE-Fluc, Ad-TCF-Fluc, Ad-CMV-GFP, and Ad-CMV-Flag-SMAD7 adenoviruses.

### Cell culture treatment and small molecule compounds

Cell cultures were treated with rhTGF-β1 (100-21-100ug, Capsugel Australia PTY LTD), EVs, or EV-depleted conditioned medium to activate the TGF-β signaling pathway. EVs were used to treat cell cultures based on total protein content or TGF-β activity as indicated. Unless indicated otherwise, most experiments were done using rhTGF-β at high concentrations (2–5 ng/mL) and correspondent EV concentration. Additional reagents used in this study are described in the Supplementary information.

### Extracellular vesicle isolation

EVs were isolated from MDA231 conditioned medium by differential centrifugation and filtration (Supplementary information, Supplementary figure 3a). Briefly, MDA231 cells (2 × 10^6^ cells/dish) were cultured in Petri dishes (150 mm × 25 mm) for 48–72h or until approximately 80% confluence (approximately 8 × 10^6^ cells/dish) was observed. Thereafter, cell culture medium was aspirated and replaced by serum free medium (SFM) supplemented or not with rhTGF-β1 as indicated in the results section. MDA231 cells were incubated for 2–4h. After incubation in SFM, conditioned medium was harvested and centrifuged at 300 ×g (5 min, 4 °C) to remove floating cells and cell debris. Resulting supernatant was sequentially centrifuged at 2000 × g (15 min, 4 °C) and 3166 × g (15 min, 4 °C) to remove remaining cell debris and apoptotic bodies. After the pellet was discarded, supernatant (solution 1) was concentrated by centrifugation at 3166 × g (4 °C) in 100 K NMWL tubes (UFC910024, Amicon Ultra-15, Merck Millipore, Australia). After the concentration step was concluded, concentration tube filters were washed twice with ice-cold SFM or PBS (1×) to discard residual rhTGF-β1 present in its soluble form. Approximately 200 µL–1000 µL of filtered solution (solution 2) were transferred to microtubes and centrifuged at 10,000 × g (90 min, 4 °C). Supernatant (solution 3) was transferred to polycarbonate thick wall tubes. Large EVs (10 K pellet) were resuspended in 100 µL – 500 µL ice-cold SFM or PBS (1x) and stored (4 °C) for further analysis. Solution 3 was ultracentrifuged at 116 000 × g (20–24h, 4 °C). Resulting supernatant (solution 4) was transferred to microtubes and stored (4 °C) for further analysis. Pellets containing small EVs (100 K pellet) were resuspended in 100 µL–500 µL ice-cold SFM or PBS (1×) and stored (4 °C) for further analysis.

### Extracellular vesicle characterization

Detailed methods used for the characterization of EVs is described in the Supplementary information. In brief, total protein content of isolated EVs was quantified by bicinchoninic acid protein (BCA) assay (23225, Thermo Fisher Scientific Australia PTY LTD). The expression of the EV molecular markers was assessed by western blot. Particle size distribution and concentration in EV-containing solutions were assessed by using a NanoSight N300 (Malvern Panalytical) where the Brownian movement of each particle was tracked in videos recorded by a SCMOS camera (5 videos; 30 s/video) by using a Nanoparticle tracking analysis software (NTA 3.2 Dev Build 3.2.16). EVs were visualized by cryo-electron microscopy (cryo-EM) on formvar carbon-coated nickel grids and stained with 2% uranyl acetate solution in a Tecnai TF30 electron microscope.

### Dual luciferase reporter assay

Cells infected with Ad-CAGA-Fluc (MOI: 2000) and Ad-CMV-Gluc (MOI: 200) adenoviruses were seeded in 96-well plates (3 × 10^3^ cells/well) overnight. Cells were then treated with rhTGF-β1 or EVs at concentrations indicated in the results section. Three technical replicates were used. After treatment (24h-48h as indicated in the results section), cell culture medium was aspirated, cells were incubated with 50 µL/well Cell Culture Lyses Reagent (E1910, Promega Australia PTY LTD) under gentle agitation (30 min, 4 °C). Cell lysates (30 µL/well) were transferred to a 96-well opaque reading plate and the luciferase activity was quantified on a GloMax® 96 Microplate Luminometer by using the Dual-Luciferase® Reporter Assay System (E1910, Promega Australia PTY LTD) according to manufacturer’s instructions. TGF-β/SMAD3 signaling reporter (Ad-CAGA-Fluc) activity was normalized by Ad-CMV-Gluc activity. TGF-β/SMAD3 signaling reporter (Ad-CAGA-Fluc) activity was represented as relative light units (RLU) and the fold change was calculated relative to untreated cell cultures. Alternatively, TGF-β/SMAD3 signaling reporter (Ad-CAGA-Gluc) activity was quantified as indicated in the results section and figure legends. Similar procedures were adopted to quantify the BMP signaling reporter (Ad-Bre-Fluc) activity and the Wnt signaling reporter (Ad-TCF-Fluc) activity.

### Quantification of TGF-β activity in extracellular vesicles

TGF-β activity in EVs was quantified by dual luciferase assay in MDA-MB-231 cells infected with Ad-CAGA-FLuc and Ad-CMV-GLuc adenoviruses as previously described.^39^ To establish a standard curve, the TGF-β/SMAD3 signaling reporter (Ad-CAGA-Fluc) activity was determined in cell cultures treated with rhTGF-β1 at increasing concentrations. Simultaneously, the TGF-β/SMAD3 signaling reporter (Ad-CAGA-FLuc) activity was quantified in cell cultures treated with EVs and EV-depleted conditioned medium at increasing volumes. The TGF-β/SMAD3 signaling reporter (Ad-CAGA-Fluc) activity quantified in cell cultures treated with EVs and EV-depleted conditioned medium was interpolated in the standard curve obtained from cell cultures treated with rhTGF-β1 at increasing concentrations. The equivalent concentration of active TGF-β in EVs and EV-depleted conditioned medium was calculated by considering only the exponential phase of the TGF-β standard curve, typically obtained in cells treated with 0.01–1.0 ng/mL rhTGF-β1.

### Western blotting

In brief, cell cultures treated ± rhTGF-β1 or EVs (concentration and time indicated in the results section) were grown until 90–100% in confluence. Protein extracts obtained after cell lysis were separated by electrophoresis followed by a transference step to a nitrocellulose membrane. Nitrocellulose membranes were blocked before incubation with primary antibody solution, followed by incubation with a secondary antibody conjugated to horseradish peroxidase. The signal was visualized and imaged by a CCD camera. Further information and a list of reagents is described in the Supplementary information.

### Immunofluorescence staining

In brief, cell cultures were treated ± rhTGF-β1 or EVs and cultured until 70–80% confluence. Cells were then fixed, permeabilized and blocked before incubation with primary antibody and secondary antibody conjugated to Alexa Fluor® 546. Cell nuclei were stained with Hoechst 33342. Fluorescence was visualized by a CCD camera coupled to a fluorescent microscope. Additional information and a list of reagents is described in the Supplementary information.

### Wound healing assay

Cell cultures (80–90% confluent) were manually scratched with a P200 pipette tip to obtain a straight wound in the monolayer. Cell culture medium was aspirated, cell cultures were washed with PBS (1×) to remove floating cells, and fresh culture medium supplemented with reduced serum (1% FCS) was added. Cell cultures treated ± rhTGF-β1 or EVs immediately after scratching were imaged at 0 h and 24 h after treatment by a CCD camera coupled to microscope (4× magnification). The wounded area was quantified by using the ImageJ software and cell migration was determined by considering the wounded area observed 24 h after treatment relative to the wound area 0 h after treatment.

### Cell migration assay and cell invasion assay in transwell inserts

Cells were seeded on top of the membrane of transwell polycarbonate inserts (8.0 μm pore size) and simultaneously treated ± rhTGF-β1 or EVs for 24 h in cell culture medium supplemented with reduced serum (1% FCS). Culture medium was then aspirated from the upper chamber of transwell inserts and non-migrated cells were removed with cotton buds dipped in PBS (1×). Migrated cells were fixed in 3.7% formaldehyde (5 min, RT), washed with PBS (1×), and cell nuclei were stained with Hoechst 33342 (5117, Tocris, In Vitro Technologie Pty Ltd, Australia) (10 min, RT). Staining solution was aspirated and transwell inserts were washed with PBS (1×). Cell nuclei were then visualized and counted by using a CCD camera coupled to a fluorescent microscope (10–20× magnification). Alternatively, Gaussia luciferase-labeled cells were seeded in transwell inserts and treated as before for cell migration quantification as described in the Supplementary information.

### Quantification of TGF-β/SMAD3 signaling activity in vivo

Six-week-old female mice homozygous for the severe combined immune deficiency spontaneous mutation in DNA dependent protein kinase active subunit Prkdc^SCID^ (NOD-SCID mice) were purchased from Animal Resource Centre (ARC, West Australia). All animal experiments were performed in accordance with National Health Medical Research Council of Australia code of practice for the care and use of animals for scientific purposes and approved by The University of Melbourne Animal Ethics Committee (Ethics ID: 1914917).

To establish a model of metastatic breast cancers highly responsive to TGF-β, NOD-SCID mice were orthotopically and contralaterally implanted with MDA231 cells (1× MDA231 tumor/mouse; right mammary fat pad) and Gaussia luciferase-labeled MDA231 (MDA.Gluc) cells (1× MDA.Gluc tumor/mouse; left mammary fat pad). To quantify the TGF-β/SMAD3 signaling activity in primary tumors,^39,40^ MDA.Gluc tumors infected with TGF-β/SMAD3 signaling reporter (Ad-CAGA-Fluc) were injected with 20 µL solution containing 5 µg MDA231-sEVs (2 ng TGF-β activity) or vehicle (PBS 1×). Intratumoral injections were administered twice weekly (dpi 17-27) until in vivo quantification of the TGF-β/SMAD3 signaling activity in MDA.Gluc tumors. Alternatively, NOD-SCID mice contralaterally implanted with MDA231 and MDA.Gluc cells were treated with DMA ± SB431542 or vehicle (DMSO) via intraperitoneal injections. DMA and SB431542 were administered five times (dpi 8-19) until in vivo quantification of the TGF-β/SMAD3 signaling in MDA.Gluc tumors.

Alternatively, to establish a model of poorly metastatic breast cancer cells with low response to TGF-β, NOD-SCID mice were orthotopically and contralaterally implanted with MCF7 cells (1x MCF7 tumor/mouse; right mammary fat pad) and Gaussia luciferase-labeled MCF7 (MCF7.Gluc) cells (1x MCF7.Gluc tumor/mouse; left mammary fat pad). Mice were intratumorally injected with MDA231-sEVs or vehicle (dpi 54-78). Seven injections per animal were administered until in vivo quantification of the TGF-β/SMAD3 signaling activity in MCF7.Gluc tumors infected with TGF-β/SMAD3 signaling reporter (Ad-CAGA-Fluc).

The bioluminescence signal emitted by the fat pad area was quantified using an In Vivo Imaging System (IVIS 200 Series, Caliper Life Sciences).^39,40^ Mice lying supine were imaged each 1–2 min for 30 min following intraperitoneal injection with 150 mg/kg VivoGlo™ Luciferin, In Vivo Grade (P1043, Promega Corporation Australia). To image different areas of target tumors aiming at establishing the maximum TGF-β/SMAD3 signaling activity, mice were tilted on their flank after the emitted bioluminescence reached a plateau. Animals were imaged individually or simultaneously with 1–2 additional mice. The signal intensity was analyzed using total flux (photons/second) in the regions of interest and normalized to background signal by Living Image software (V3.2, Caliper Life Sciences).

### Quantification of circulating tumor cells, metastasis, and tumor self-seeding ex vivo

Mice were implanted with human breast cancer cells as described in this section (Quantification of TGF-β/SMAD3 signaling activity in vivo) and animal weight and tumor growth were monitored 2–3 times per week. Once palpable tumors were detected, tumor volume was calculated based on caliper measurements. To calculate a tumor volume, the tumor’s largest dimension (d_a_) and smallest dimension (d_b_) were used in Eq. (2). Tumors and tissues/organs were collected for analysis after animal euthanasia as indicated in the results section.

2 $$\frac{{d}_{a}\times {d}_{b}^{2}}{2}$$

To evaluate breast cancer progression, blood samples, peripherical organs, and tumors were harvested immediately after mice euthanasia. The detection of MDA.Gluc or MCF7.Gluc CTCs was assessed by quantifying the Gaussia luciferase activity in lysed blood samples (5 µL/sample; 5 samples/mouse).^6^ The presence of metastatic Gaussia luciferase-labeled cells was evaluated in lung, liver, brain, and bone samples.^6^ For this purpose, each organ was individually cut in several fragments, and five fragments were randomly selected for lysis and ex vivo quantitation of Gaussia luciferase activity by luciferase assay (5 mg/fragment; 5 fragments/organ/mouse). Tumor self-seeding^41^ was similarly quantified by ex vivo analysis of the Gaussia luciferase activity in unlabeled tumors (5 mg/fragment; 5 fragments/tumor/mouse).^6^ Two NOD-SCID mice at the same cohorts were not implanted with cancer cells (non-implanted animals) and used as negative controls.

### Statistics

Most data from in vitro experiments are represented as the mean ± standard deviation (SD), unless otherwise indicated in figure legends. Data from in vivo and ex vivo experiments are represented as the mean ± standard error (SEM). Data distribution was analyzed by Kolmogorov–Smirnov test and groups were compared by unpaired Student’s *t*-test or analysis of variance (ANOVA) if parametric. Non-parametric correspondent tests and post-tests were performed if necessary and are indicated in figure legends. The Kaplan–Meier method was used to plot survival probability curves. Log-rank analysis was used to compare the differential survival of breast cancer patients. Spearman’s rank correlation coefficient or Pearson’s rank correlation coefficient was used to analyze the correlation between gene signature scores as indicated. Multivariate Cox analysis was used to analyze the correlation between gene signature scores and breast cancer patient survival. Only two-sided tests were used. Differences between groups were considered significant when *p* < 0.05. Statistical analyses were performed by InStatGraphpad software (GraphPadInStat version 6.0, GraphPad Prism Software).

### Supplementary information

Supplementary Information

## Data Availability

All data supporting the findings of this study are available within the article and its Supplementary Information files. Raw data is available from the corresponding authors upon request.
