# Supplementary material for: Simultaneously targeting extracellular vesicle trafficking and TGF-β receptor kinase activity blocks signaling hyperactivation and metastasis
Source: Signal Transduct Target Ther. 2023 Dec 18;8:456. doi: 10.1038/s41392-023-01711-1 (PMC10725874; doi:10.1038/s41392-023-01711-1)
Supplement: Supplementary file 1 — Supplementary Information [file 41392_2023_1711_MOESM1_ESM.docx]

**Supplementary Information**

**Simultaneously targeting extracellular vesicle trafficking and TGF-β receptor kinase activity blocks signaling hyperactivation and metastasis**

Adilson Fonseca Teixeira^1,2^, Yanhong Wang^1^, Josephine Iaria^1,2^, Peter ten Dijke^3^, and Hong-Jian Zhu^1,2,*^

^*^**Correspondence:** Department of Surgery, The University of Melbourne, 5th Floor Clinical Sciences Building, The Royal Melbourne Hospital, Parkville 3050, Australia (Tel: 61 3 8344 3025; Fax: 61 3 9347 6488, email: [hongjian@unimelb.edu.au](mailto:hongjian@unimelb.edu.au))

**This file includes:**

Supplementary materials and methods

Supplementary figures 1-14

Supplementary tables 1-12

**Supplementary materials and methods**

**Gene expression and gene signature scores**

Kaplan Meier curves generated by the web-based tool USCS Xena Browser (https://xenabrowser.net) were used to evaluate the association between the expression of individual exosome-related genes and the survival of breast cancer patients in the TCGA BRCA and NKI-295 datasets. High and low expression of exosome-related genes were determined by raking samples according to gene expression and dichotomizing the cohort at the 50th percentile. Alternatively, top and bottom quartiles were compared to represent patient samples with high or low gene expression, respectively.

Exosome-related genes were categorized into four biological processes: (i) enriched in Rab GTPase and associated proteins (Endosome maturation), (ii) enriched in retromer complex proteins and associated molecular partners (Retromer complex), (iii) enriched in ESCRT complex proteins and associated molecular partners (ESCRT complexes), and (iv) enriched in SNARE complex proteins and associated molecular partners (SNARE complexes).

**Gene mutation analysis**

Mutations in exosome-related genes in breast cancers were analyzed in the TCGA BRCA dataset by using the National Cancer Institute (NIH) Genomic Data Commons (GDC) Data Portal (https://portal.gdc.cancer.gov/). The ‘exploration’ tab was selected to obtain the required genomic information and breast cancer cases were filtered by selecting: ‘program: TCGA’, ‘project: TCGA-BRCA’, ‘sample type: primary tumor’, ‘available variation data: ssm’. Clinical data was not used for sample exclusion, resulting in 965 samples evaluated. Exosome-related genes were individually analyzed in the tab ‘genes’ by computing the number of affected cases in the selected cohort, type of mutation, and consequences for the canonical transcript. Predicted impacts for the canonical transcripts were analyzed by using the GDC plugins: Variant Effect Predictor (VEP) (http://asia.ensembl.org/info/docs/tools/vep/index.html), Sorting Intolerant From Tolerant SIFT (http://sift.jcvi.org/), and PolyPhen (<http://genetics.bwh.harvard.edu/pph2/>).

**Cell culture treatment and small molecule compounds**

Cell cultures were treated with recombinant human (rh)TGF-β1 (100-21-100ug, Capsugel Australia PTY LTD), extracellular vesicles (EVs), or EV-depleted conditioned medium to activate the TGF-β signaling pathway. EVs were used to treat cell cultures based on total protein content or TGF-β activity as indicated. Unless indicated otherwise, most experiments were done using rhTGF-β at high concentrations (2-5 ng/mL) and correspondent EV concentration. Cell cultures treated with rhBMP4 (120-05, PeproTech, Australia) were used as positive controls for the quantification of the BMP-SMAD1/5 signaling reporter (Ad-Bre-Fluc) activity. Cell cultures treated with Wnt3a (H17001, Sigma-Aldrich, Merck PTY LTD, Australia) were used as positive controls for the quantification of the Wnt/TCF signaling reporter (Ad-TCF-Fluc) activity. In certain experiments, cells were treated with the TGFβ type 1 receptor inhibitor SB431542 (S4317, Sigma-Aldrich, Merck PTY LTD, Australia) to inhibit the TGF-β signaling pathway. Unless indicated otherwise, 2 µM SB431542 were used. Alternatively, TGF-β signaling inhibition was achieved by infecting cells with Ad-CMV-Flag-SMAD7 adenovirus (home-made) to induce SMAD7 overexpression. EV trafficking was targeted by treatment with 5-(N,N-Dimethyl)amiloride hydrochloride (DMA) (A4562, Sigma-Aldrich, Merck PTY LTD, Australia), heparin (H3393, Sigma-Aldrich, Merck PTY LTD, Australia), or 4-Nitrophenyl β-D-xylopyranoside (PNP-Xyl) (N2132, Sigma-Aldrich, Merck PTY LTD, Australia). Unless indicated otherwise, 100 µM DMA, 100 µg/mL heparin, and 2.5 mM PNP-Xyl were used.

**Extracellular vesicle characterization**

Total protein content of isolated extracellular vesicles (EVs) was quantified by bicinchoninic acid protein (BCA) assay. In brief, aliquots of EV-containing solution (1-5 µL) were mixed with 200 µL Pierce BCA Protein Assay Kit (23225, Thermo Fisher Scientific Australia PTY LTD), incubated for 30 minutes at 37 ºC, and the solution absorbance was quantified at 562 nm according to the manufacturer’s instructions. Protein concentration in EV-containing solution was established by interpolation in a bovine serum albumin (BSA) standard curve. The expression of the EV molecular markers Alix, TSG101, and CD63 were assessed by western blot. Antibodies used for western blot are listed below.

Particle size distribution and concentration in EV-containing solutions were assessed by using a NanoSight N300 equipped with a 488 nm blue laser to detect and record scattered light from individual particles (Malvern Panalytical). Samples were diluted 1:20–1:100 in PBS (1x) (viscosity 0.925 - 0.929 cP) to achieve a concentration within the recommended measurement range (1x106 to 1x109 particles/mL). NanoSight syringe pump and a script control system were used for automatic sample injection. The Brownian movement of each particle was tracked in videos recorded by a SCMOS camera (5 videos; 30s/video) by using a Nanoparticle tracking analysis software (NTA 3.2 Dev Build 3.2.16). Individual particle tracks (10-2000 nm) were analyzed based on the particle’s hydrodynamics and considering the Stokes−Einstein equation. Capture settings were defined considering 25 frames/second (FPS) at 23 ºC. Camera detection threshold and blur size were set to 9 and ‘auto’, respectively. Post-acquisition settings optimized for NTA were defined and used to compare distinct samples. NTA was used to calculate the mean, mode, and median size of analyzed particles and to define the particle concentration in each EV sample.

Extracellular vesicles isolated from MDA231-conditioned medium were visualized by cryo-electron microscopy (cryo-EM). MDA231-EVs resuspended in PBS (1x) were dropped on formvar carbon-coated nickel grids and stained with 2% uranyl acetate solution. Excess fluid was carefully removed, and grids were air-dried for visualization in a Tecnai TF30 electron microscope. The equipment coupled to a HAADF STEM detector, a Gatan quantum 965 energy filter, and an upper CETA 4x4k CMOS camera was operated at 300kV.

**Western blotting**

Cell cultures treated ± recombinant human (rh)TGF-β1 or extracellular vesicles (EVs) (concentration and time indicated in the results section) were grown until 90-100% in confluence. Harvested cells were incubated with ice-cold lysis buffer (30mM HEPES, 1% TritonX-100, 2mM MgCl_2_, 150mM NaCl, 5mM EDTA, complete protease inhibitor tablet and phosphostop phosphatase cocktail tablet) under agitation (30 min, 4 ºC). Cell lysates were transferred to microtubes, and cell debris were eliminated by centrifugation at 1 3000 rpm (15 min, 4 ºC). Protein concentration was determined in supernatant by using a bicinchoninic acid protein assay. Supernatant was then mixed with Laemmli sample buffer (4x) (Bio-Rad laboratories PTY LTD, Australia) and proteins were heat-denatured (10 min, 95 ºC). Protein separation was done in Bolt™ 4-12%, Bis-Tris, 1.0 mm, Mini Protein Gels (Thermo Fisher Scientific, Australia PTY LTD) followed by a transference step to a nitrocellulose membrane using an iBlot 2 Dry Blotting System (Invitrogen, Thermo Fisher Scientific). Nitrocellulose membranes were blocked by incubation in 5% skim milk solution (1h, RT) and washed (30 min, RT) before incubation with primary antibody solution (4 ºC, overnight). Membranes were washed again and incubated with a solution containing a secondary antibody conjugated to horseradish peroxidase (HPR) (1h-2h, RT). The signal was visualized by using the Western Lightning® ECL Pro Enhanced Chemiluminescence Substrate NEL121001EA (PerkinElmer PTY LTD Australia) and imaged by a CCD camera in a ChemiDoc MP Imaging System® (Bio-Rad laboratories). Total SMAD2 levels and/or β-Actin levels were used as loading controls. The antibodies were raised against the following proteins: phospho-SMAD2 (pSMAD2, home-made) (Persson *et al*., 1998), SMAD2 (610843, Becton Dickinson PTY LTD (BD), Australia), ZO-1 (610967, Becton Dickinson PTY LTD (BD), Australia), E-cadherin (610182, Becton Dickinson PTY LTD (BD), Australia), Rab27a (Ab55667, Abcam, Australia), Flag M2 (F3165, Sigma-Aldrich, Merck PTY LTD, Australia), β-actin (A5441, Sigma-Aldrich, Merck PTY LTD, Australia), Alix (2171S, Cell Signaling Technology, New England Biolabs, Australia), TSG101 (612696, Becton Dickinson PTY LTD (BD), Australia), and CD63 (sc-5275, Santa Cruz Biotechnology, Bio-Strategy Pty Limited, Australia). Goat anti-rabbit IgG (H+L)-HRP conjugate (1706515, Bio-Rad Laboratories PTY LTD, Australia) and goat anti-mouse (H+L)-HRP conjugate (1706516, Bio-Rad Laboratories PTY LTD, Australia) were used as secondary antibodies.

**Immunofluorescence staining**

Cell cultures were treated ± recombinant human (rh)TGF-β1 or extracellular vesicles (EVs) (concentration and time indicated in the results section) and cultured until 70-80% confluence. Cells were then fixed in a solution of 3.7% formaldehyde (5 min, RT), washed once in PBS (1x), permeabilized with 0.1% Triton-X-100 (5 min, RT), and washed again in PBS (1x). Permeabilized cells were blocked in 5% BSA (1h, RT). Cell cultures were then incubated with primary antibody (1h-2h, RT), washed once in PBS (1x) and incubated with secondary antibody conjugated to Alexa Fluor® 546 (1h-2h, RT) while protected from light. Cell cultures were washed again in PBS (1x) and cell nuclei were stained with Hoechst 33342 (5117, Tocris, In Vitro Technologie Pty Ltd, Australia) (10 min, RT) while protected from light. After PBS (1x)- and double-distilled water- washing, fluorescence was visualized by a CCD camera coupled to a fluorescent microscope (20-40x magnification). The antibodies were raised against the following proteins: ZO-1 (610967, Becton Dickinson PTY LTD (BD), Australia), E-cadherin (610182, Becton Dickinson PTY LTD (BD), Australia), and Flag M2 (F3165, Sigma-Aldrich, Merck PTY LTD, Australia). Goat anti-mouse IgG (H+L) Alexa Fluor™ 546 (A-11003, Thermo Fisher Scientific, Australia PTY LTD) was used as a secondary antibody.

**Quantification of cell numbers *in vitro***

Cell proliferation and numbers were analyzed by quantifying the Gaussia luciferase activity in MDA.Gluc breast cancer cells or MDA231 cells infected with Ad-CMV-Gluc adenovirus. Gaussia luciferase-labeled cells were titered and the Gaussia luciferase activity quantified by luciferase assay was correlated with cell numbers. Luciferase assay was done using cell lysates as described in this section (Dual luciferase reporter assay).

**Gaussia luciferase-labeled cell migration/invasion assay in transwell inserts**

Gaussia luciferase-labeled cells were seeded in transwell inserts and treated ± recombinant human (rh)TGF-β1 or extracellular vesicles. Cell culture was aspirated 24h after treatment and cell cultures were washed with PBS (1x). A scalpel blade was used to gently remove the membrane of transwell inserts that were then transferred to empty wells, covered with 100 µL Cell Culture Lyses Reagent (E1910, Promega Australia PTY LTD), and incubated under gentle agitation (30 min, 4 ºC). Cell lysate (30 µL/well) were transferred to a 96-well opaque reading plate in duplicates and the Gaussia luciferase activity was quantified on a GloMax® 96 Microplate Luminometer by using Dual-Luciferase® Reporter Assay System (E1910, Promega Australia PTY LTD) according to manufacturer’s instructions. Gaussia luciferase activity was presented as relative light units (RLU) and the migration fold change was calculated relative to untreated cell cultures. The correlation between cell numbers and Gaussia luciferase activity was established by cell titration as described in the section ‘Quantification of cell numbers *in vitro*’. Cell invasion was similarly quantified by adjusting this protocol. Cells were seeded on top of the membrane of Matrigel-coated transwell inserts, treated as before, and cultured for 48h. After removing non-invasive cells, invasive cells were stained with Hoechst 33342 for visualization and counting using a CCD camera coupled to a fluorescent microscope (10-20x magnification).

**
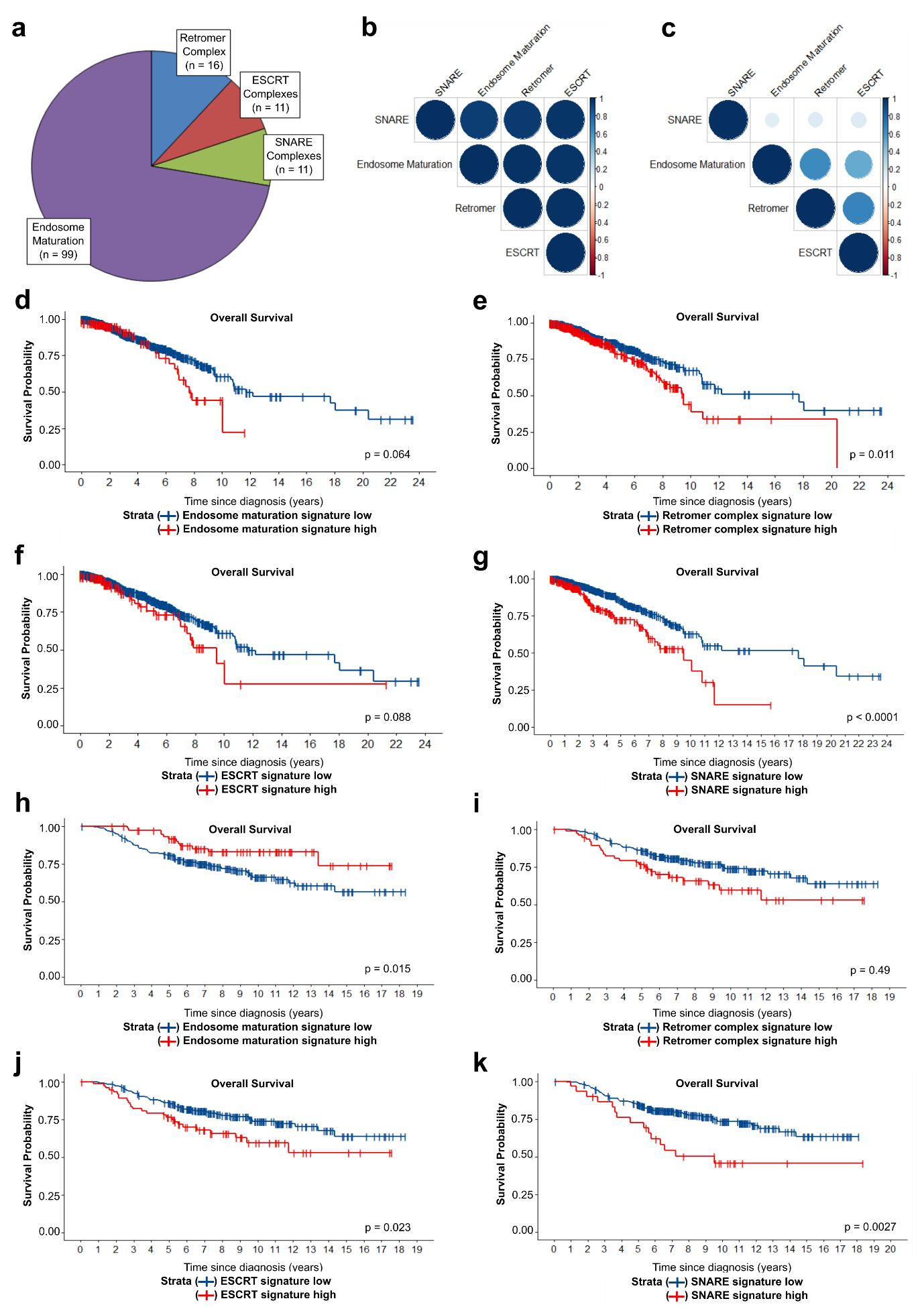
**

**Supplementary figure 1: Additional characterization of the correlation between the expression of exosome-related genes and the overall survival of breast cancer patients, related to Figure 1. (a)** Proportional distribution of genes associated with the main steps required for exosome biogenesis and secretion. **(b-c)** Correlation matrix for the expression of exosome-related genes according to the steps required for exosome biogenesis and secretion in the **(b)** TCGA BRCA cohort and the **(c)** NKI-295 cohort calculated according to the Spearman's rank correlation coefficient. Color bar indicates the correlation coefficient. Only statistically significant correlations (p < 0.05) are represented. Kaplan-Meier curves showing the overall survival for breast cancer patients (TCGA BRCA cohort) stratified by **(d)** endosome maturation signature, **(e)** retromer signature, **(f)** ESCRT signature, and **(g)** SNARE signature. Kaplan-Meier curves showing the overall survival for breast cancer patients (NKI-295 cohort) stratified by **(h)** endosome maturation signature, **(i)** retromer signature, **(j)** ESCRT signature, and **(k)** SNARE signature. Log-rank test was used to analyze survival probability.


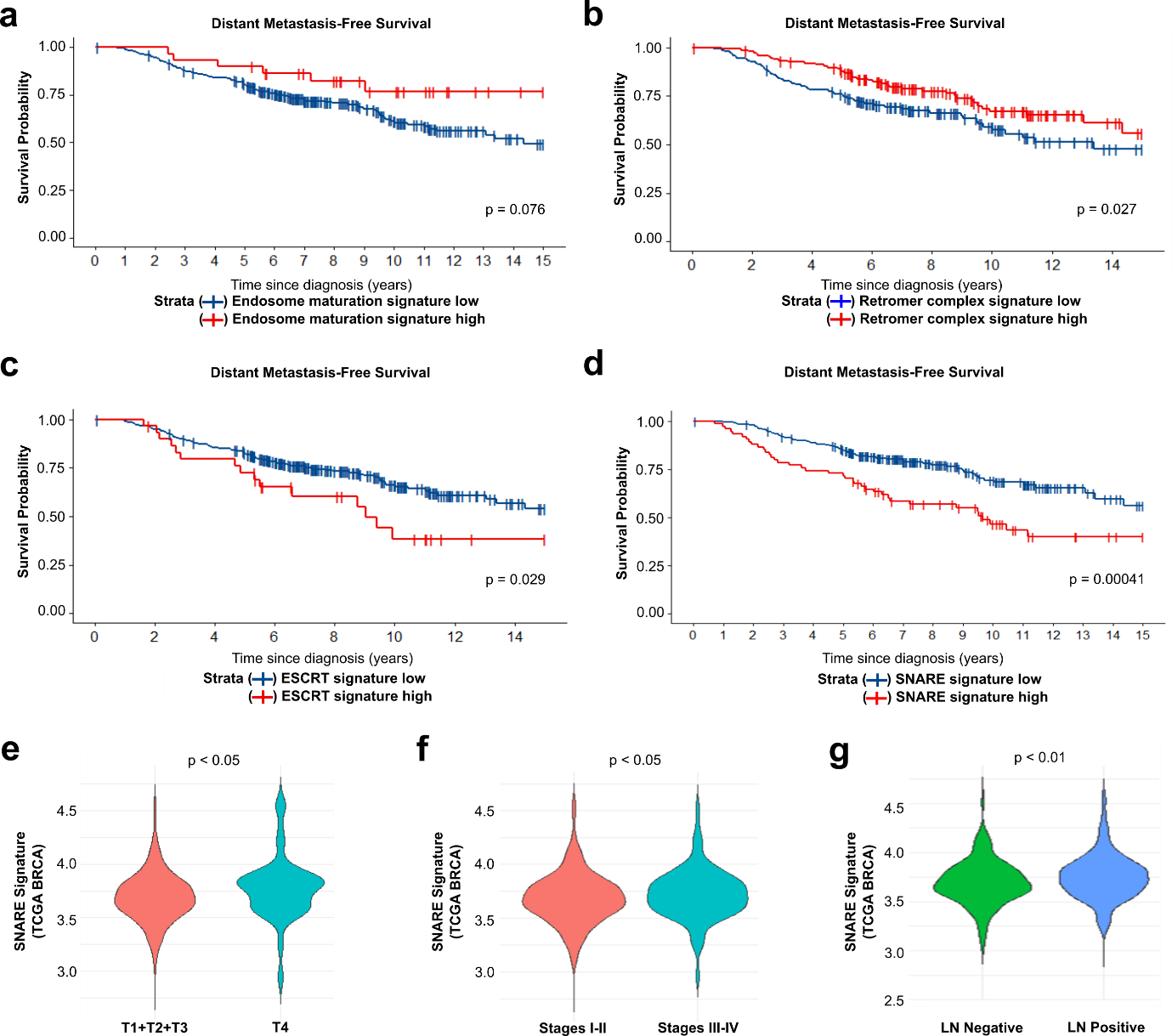


**Supplementary figure 2: Additional characterization of the correlation between the expression of exosome-related genes and the progression of breast cancer patients, related to Figure 1.** Kaplan-Meier curves showing the distant metastasis-free survival for breast cancer patients (NKI-295 cohort) stratified by **(a)** endosome maturation signature, **(b)** retromer signature, **(c)** ESCRT signature, and **(d)** SNARE signature. Log-rank test was used to analyze survival probability. Violin plots comparing the SNARE-related gene signature in breast cancers **(e)** at different sizes (T stage), **(f)** at different stages (stages I+II versus stages III+IV) and **(g)** negative or positive for lymph node (LN) metastasis as analyzed by Unpaired Student’s t test (TCGA BRCA cohort).

**
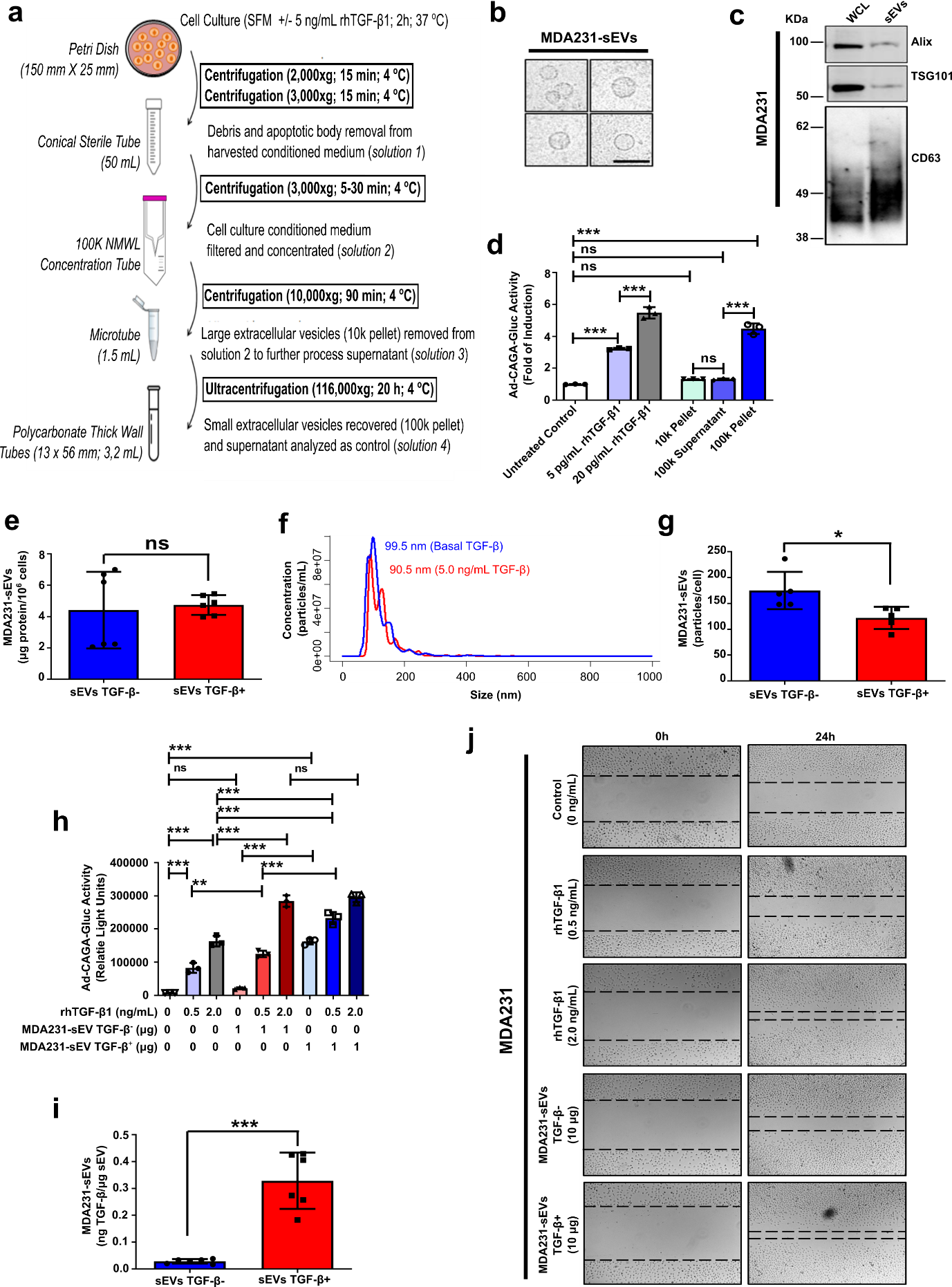
**

**Supplementary figure 3: Characterization of isolated small extracellular vesicles (sEVs) and validation of sEV-induced TGF-β signaling activity, related to Figure 2. (a)** Schematic illustration showing the procedure adopted for sEV isolation. Breast cells were cultured in serum-free medium supplemented or not with recombinant human (rh)TGF-β1. Cell culture conditioned medium was harvested 2-4h after starvation started and EVs were isolated by combining ultrafiltration (100K NMWL tubes) and differential ultracentrifugation. **(b)** The morphology of MDA231-sEVs was evaluated by cryogenic electron microscopy. Scale bar is equal to 200 nm. **(c)** The expression of extracellular vesicle markers was assessed in MDA231-derived whole cell lysates (WCL) and sEVs protein extracts by western blot. **(d)** The TGF-β/SMAD3 signaling reporter (Ad-CAGA-Gluc) activity was quantified in MDA231 cells treated with MDA231-EVs for 24h. Large (l)EVs (10K pellet), small (s)EVs (100K pellet), and EV-depleted conditioned medium (100K supernatant) were tested. Treatment with rhTGF-β1 was used as positive control. **(e-i)** sEVs isolated from MDA231 cell culture conditioned medium treated (TGF-β^+^) or not treated (TGF-β^-^) with 5 ng/mL rhTGF-β1 for 2h were compared. **(e)** The total sEV secretion was quantified by BCA assay in sEV protein extracts. **(f)** Particle size distribution and **(g)** concentration were evaluated by nanoparticle tracking analysis (NTA). **(h)** The TGF-β/SMAD3 signaling reporter (Ad-CAGA-Gluc) activity was assessed in MDA231-sEVs isolated from the conditioned medium of cell cultures treated or not treated with rhTGF-β1 during cell culture starvation. Treatment with rhTGF-β1 was used as positive control. **(i)** TGF-β activity in MDA231-sEVs was normalized per µg of sEV total protein. **(j)** MDA231 cell migration was evaluated by wound healing assay in cells treated with MDA231-sEVs (TGF-β^+^ or TGF-β^-^) for 24h. Treatment with rhTGF-β1 was used as positive control. Results represent mean ± SD (n≥3). One-way ANOVA test followed by Dunn's Multiple Comparison test was used to analyze data in **(d&h).** Unpaired Student’s t-test was used to analyze data in **(e, g, i)**. ns: statistically non-significant, *p < 0.05, **p < 0.01, ***p < 0.001.

**
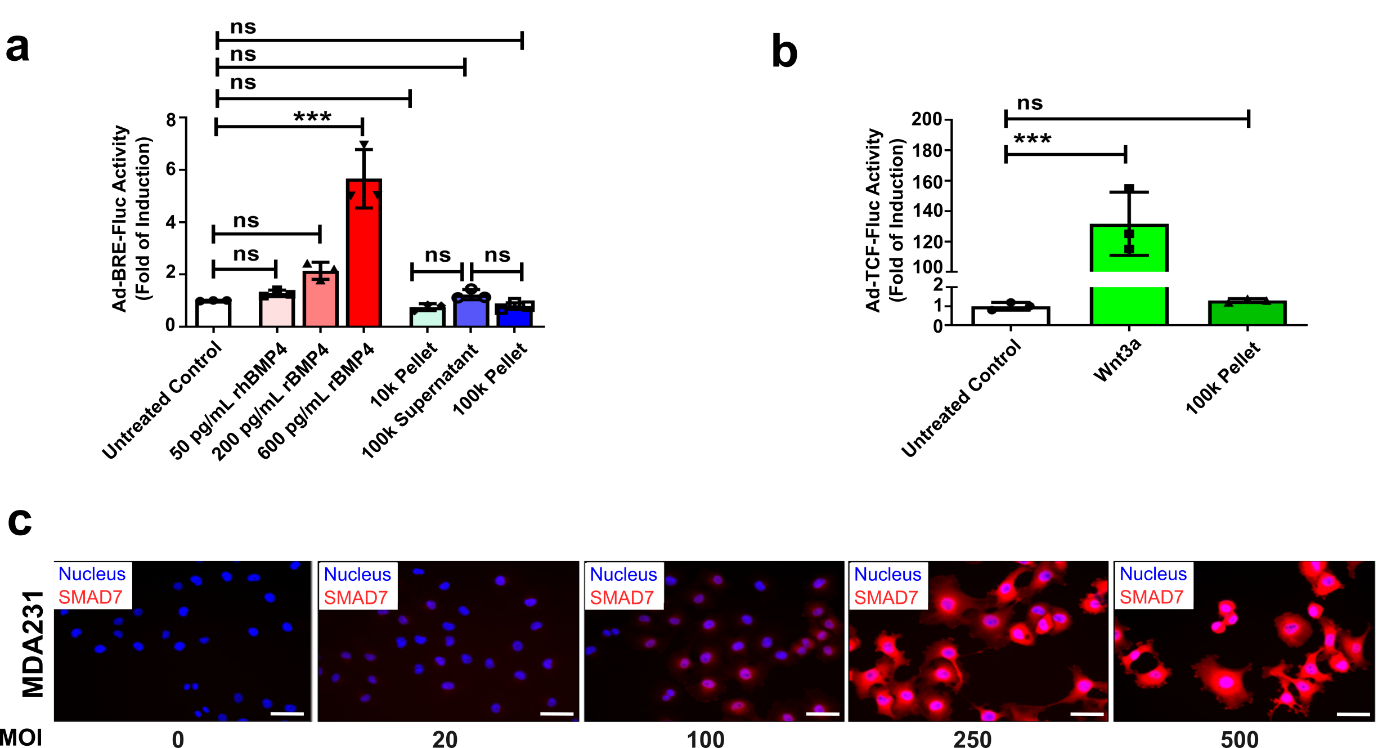
**

**Supplementary figure 4: Additional analyses of isolated MDA231-sEVs, related to Figure 2. (a)** The BMP-SMAD1/5 signaling reporter (Ad-BRE-Fluc) activity was quantified in MDA231 cells treated with MDA231-sEVs. Cell cultures treated with rhBMP4 were used as positive controls. **(b)** The Wnt/TCF signaling reporter (Ad-TCF-Fluc) activity was quantified in MDA231 cells treated with MDA231-sEVs. Cell cultures treated with Wnt3a (50 ng/mL) were used as positive control. **(c)** Exogenous SMAD7 expression was assessed by evaluating the M2-Flag expression in MDA231 cells by immunofluorescence staining. Cell cultures were infected with Ad-CMV-Flag-SMAD7 adenovirus at increasing multiplicity of infection (MOI). Non-infected cells were used as controls. Scale bar is equal to 50 µm. Results represent mean ± SD (n≥3). One-way ANOVA test followed by Dunn's Multiple Comparison test. ns: statistically non-significant, ***p < 0.001.

**
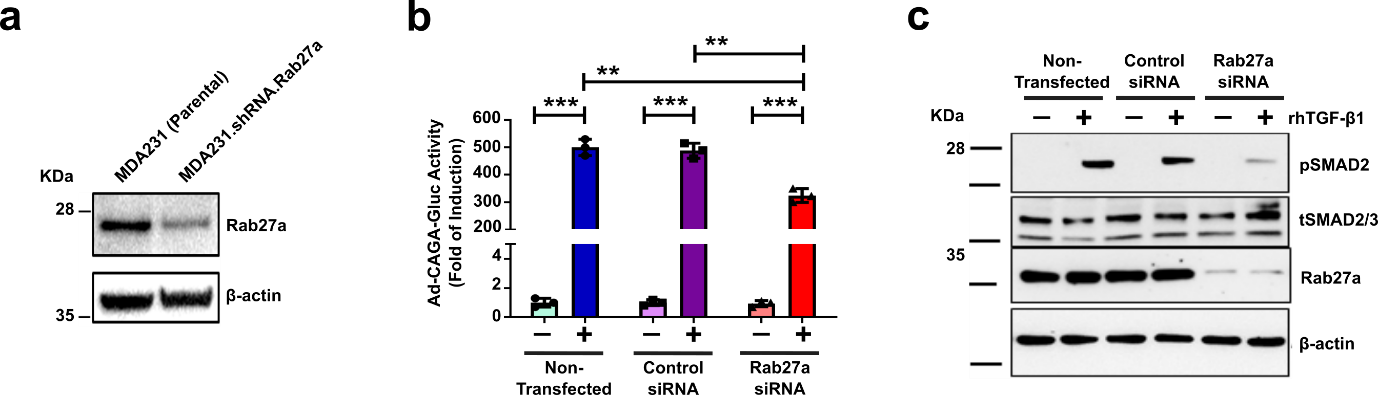
Supplementary figure 5: Validation of biochemical effects associated with Rab27a KD in MDA231 cells, related to Figure 3. (a)** Rab27a expression levels evaluated in parental MDA231 and MDA.Rab27a.shRNA cells. β-actin levels were used as loading control. **(b)** The TGF-β/SMAD3 reporter (Ad-CAGA-Fluc) activity was quantified in MDA231 cells transfected with control siRNA or Rab27a siRNA. Cell cultures were treated ± recombinant human (rh)TGF-β1 (5 ng/mL) for 24 before lysis. Results were normalized by Gaussia luciferase (Ad-CMV-Gluc) activity. Results represent mean ± SD (n≥3). One-way ANOVA test followed by Dunn's Multiple Comparison test was used to analyze obtained data. **p < 0.01, ***p < 0.001. **(c)** Phosphorylated (p)SMAD2 levels were assessed in MDA231 cells transfected with control siRNA or Rab27a siRNA and treated ± rhTGF-β1 (5 ng/mL) for 8h. Total (t)SMAD2/3 and β-actin levels were used as loading controls.

**
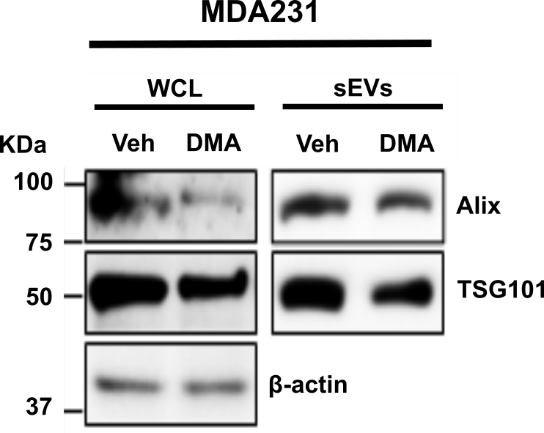
**

**Supplementary figure 6: Additional characterization of sEVs secreted by MDA231, relative to Figure 4.** sEVs were isolated from MDA231 cell culture conditioned medium after treatment ± 100 µM DMA (2h) and the expression levels of Alix and TSG101 were assessed by western blot in MDA231-derived whole cell lysates (WCL) and sEVs. Β-actin levels were used as loading controls for WCL.

**
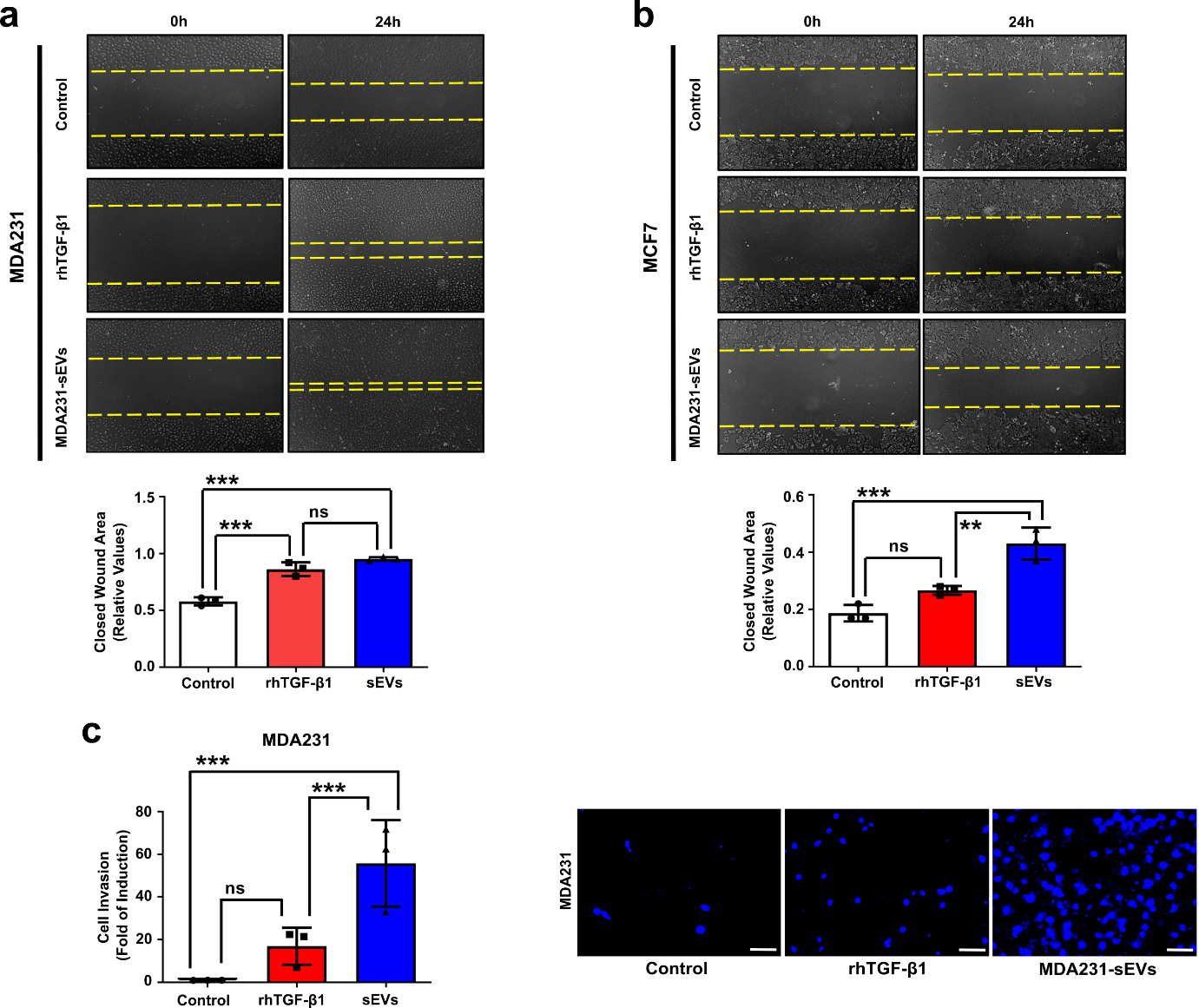
**

**Supplementary figure 7: Additional quantification of breast cell migration and invasion, related to Figure 5.** Breast cancer cell migration was quantified by wound healing assay in **(a)** MDA231 and **(b)** MCF7 cells treated ± rhTGF-β1 or MDA231-sEVs (4x magnification). **(c)** MDA231 cell invasion in Matrigel-coated transwell inserts was quantified for cell cultures treated ± rhTGF-β1 or MDA231-sEVs (48h). Scale bar is equal to 100 µm. Results represent mean ± SD (n≥3). One-Way ANOVA followed by Dunn's Multiple Comparison Test. **p<0.01, ***p<0.001, ns: statistically non-significant.

**
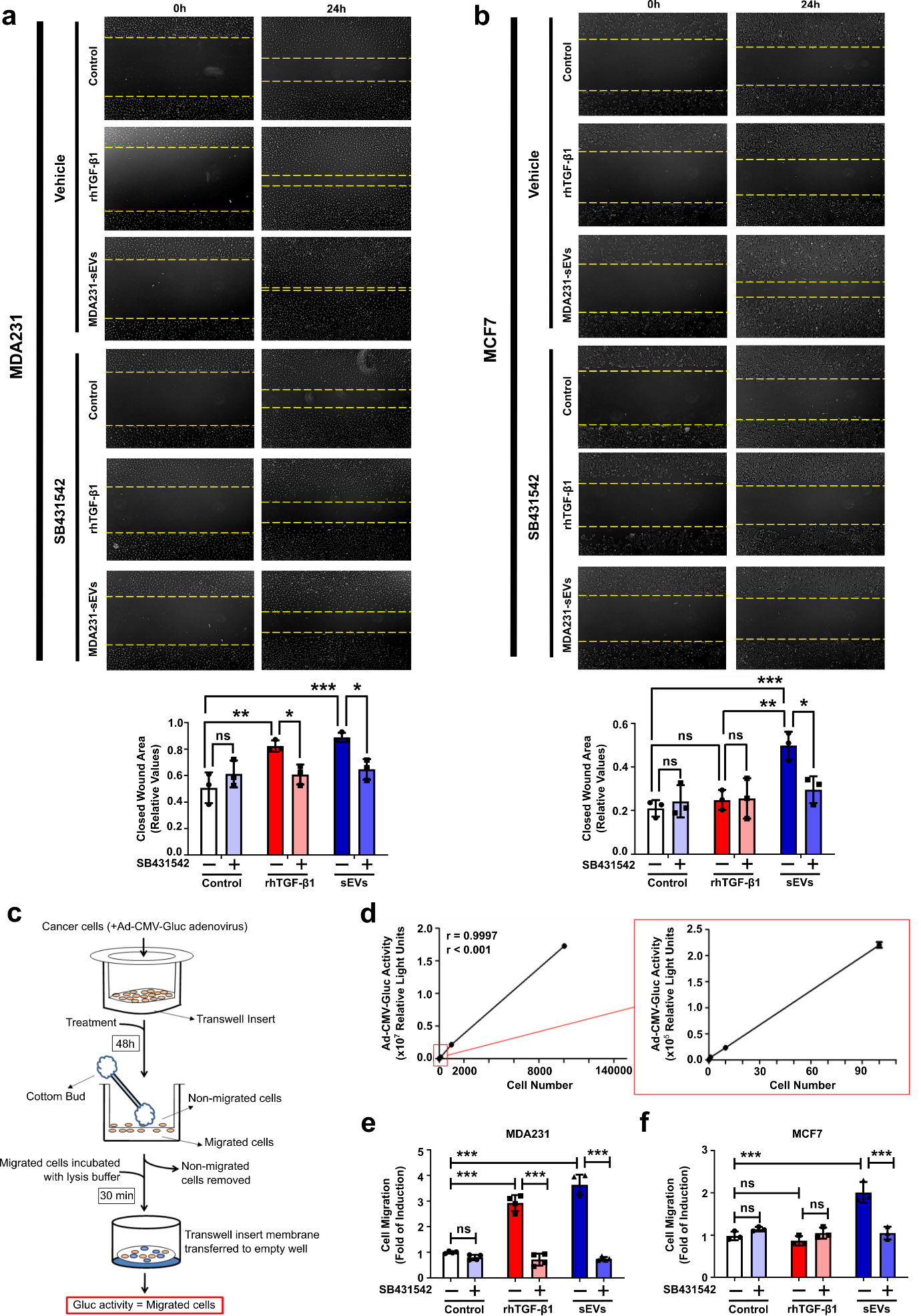
**

**Supplementary figure 8: Additional quantification of breast cancer cell migration in response to SB431542, relative to Figure 5.** Breast cancer cell migration was quantified by wound healing assay in **(a)** MDA231 and **(b)** MCF7 cell cultures treated with rhTGF-β1 or MDA231-sEVs ± SB431542 (24h). DMSO: vehicle for SB431542. **(c)** Schematic illustration showing the cell migration analysis by luciferase assay using transwell inserts. Cells infected with Ad-CMV-Gluc adenovirus are seeded on top of the membrane in transwell inserts and treated for 48h. After removal of non-migrated cells, Gaussia luciferase (Ad-CMV-Gluc) activity is quantified in cell lysates and correlated with cell numbers. **(d)** MDA231 cell number titration was evaluated by luciferase assay. Increasing cell numbers infected with Ad-CMV-Gluc adenovirus were lysed and the Gluc activity correlated with cell numbers according to Pearson's rank correlation coefficient. Results obtained with 1-100 cells are enlarged on the right panel in red. The migration of Gaussia luciferase-labeled breast cancer cells was evaluated for **(e)** MDA231 and **(f)** MCF7 cells treated ± SB431542 (2 µM) prior to stimulation with rhTGF-β1 or MDA231-sEVs. DMSO was used as a vehicle for SB431542. Results represent mean ± SD (n≥3). One-Way ANOVA followed by Dunn's Multiple Comparison Test. *p<0.05, **p<0.01, ***p<0.001, ns: statistically non-significant.

**
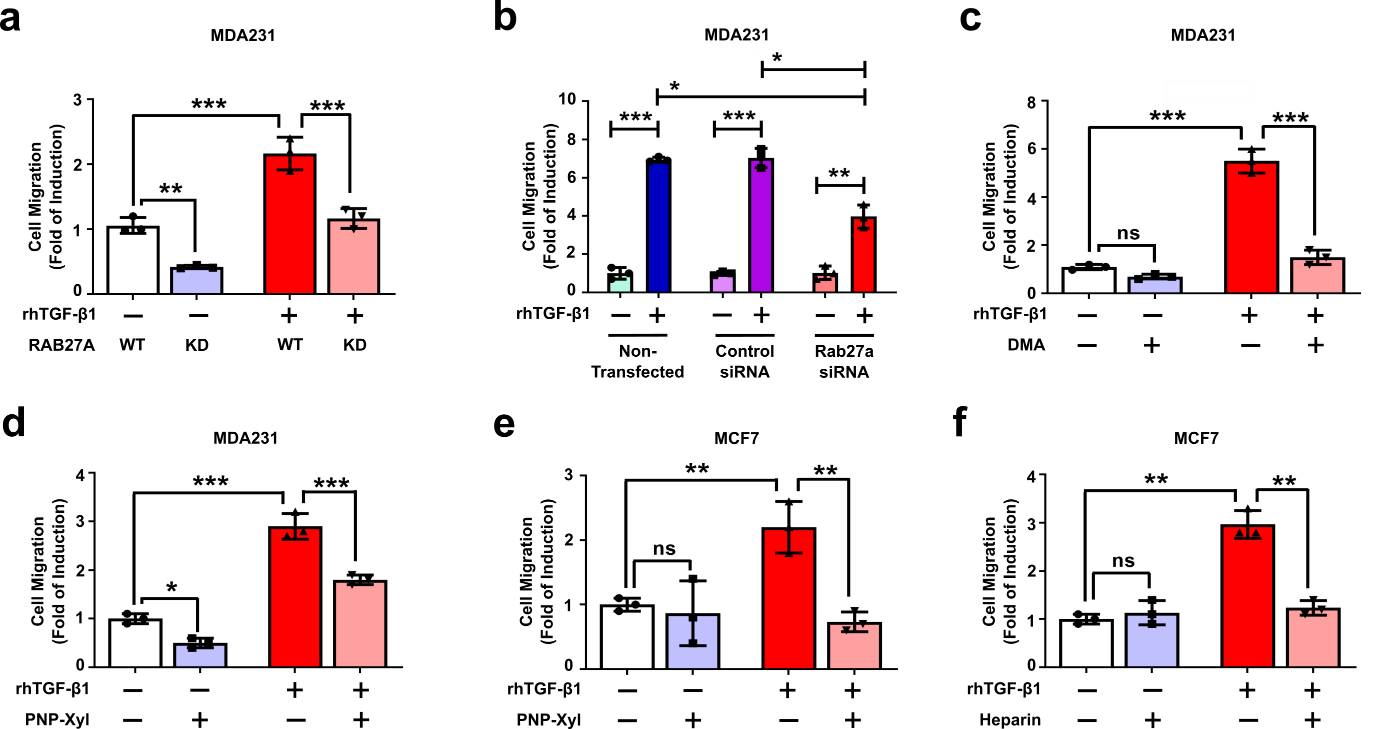
**

**Supplementary figure 9: Characterization of the effects induced by sEV trafficking inhibitors on breast cancer cell migration, relative to Figure 5. (a)** Migration of Gaussia luciferase-labeled MDA231 (WT) and MDA.Rab27a.shRNA (KD) cells treated ± rhTGF-β1 (48h) quantified by luciferase assay. **(b)** MDA231 cell migration evaluated as in **(a)** in wild type MDA231 cell cultures transfected with control siRNA or Rab27a siRNA prior to treatment with rhTGF-β1. **(c)** The migration of wild type MDA231 cells treated ± DMA (50 µM) ± rhTGF-β1 was quantified as in **(a-b)**. DMSO: vehicle for DMA. **(d-e)** Cell migration quantified as in **(a-c)** in cell cultures treated ± rhTGF-β1 or MDA231-sEVs. PNP-Xyl (2.5 mM) was used to treat **(d)** MDA231 and **(e)** MCF7 cells. DMSO: vehicle for PNP-Xyl. Heparin (100 µg/mL) was used to treat **(f)** MCF7 cells. Results represent mean ± SD (n≥3). One-Way ANOVA followed by Dunn's Multiple Comparison Test. *p<0.05, **p<0.01, ***p<0.001, ns: statistically non-significant.

**
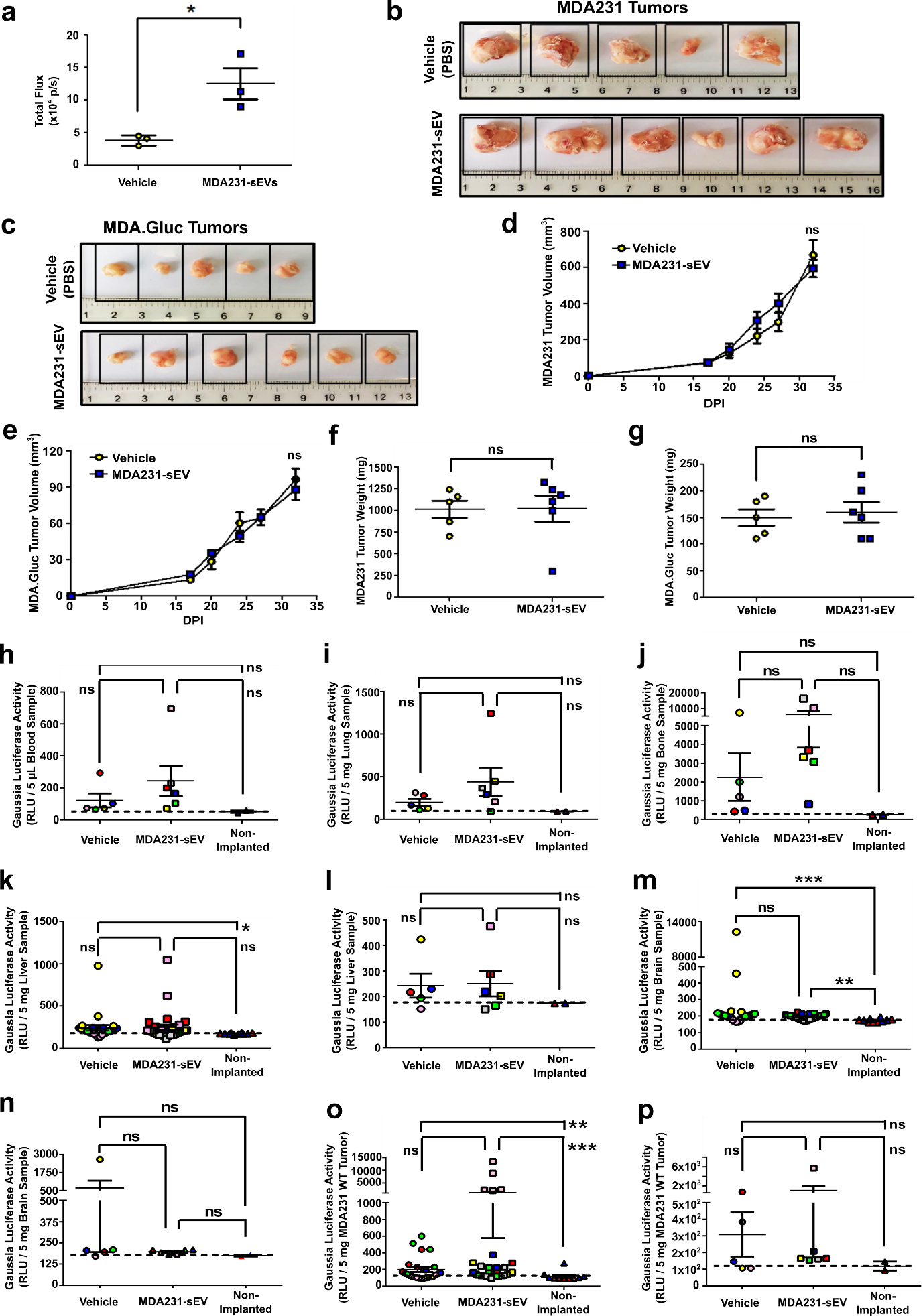
**

**Supplementary figure 10: Additional characterization for the progression of MDA231 and MDA.Gluc tumors in animals treated with MDA231-sEVs, related to Figure 6.**  **(a)** Quantification of bioluminescence emitted by MDA.Gluc tumors injected with vehicle (PBS) or MDA231-sEVs. **(b)** MDA231 and **(c)** MDA.Gluc tumors harvested from animals treated with MDA231-sEVs (or vehicle). The growth of **(d)** MDA231 and **(e)** MDA.Gluc tumors was monitored until palpable and then measured with a caliper. Tumor weights for harvested **(f)** MDA231 and **(g)** MDA.Gluc tumors. Results represent mean ± SEM. Unpaired Student’s t-test was used to analyze data in **(d-g)**. **(h-p)** Gaussia luciferase activity measured by *ex vivo* luciferase assay in **(h)** blood, **(i)** lung, **(j)** bone, **(k-l)** liver, **(m-n)** brain, and **(o-p)** unlabeled MDA231 wild type tumor samples representing the presence of MDA.Gluc cells (5-6 animals/group). Animals are color-coded. Black dashed lines indicate background activity for the Gaussia luciferase as quantified in samples from non-implanted mice (n = 2 mice). After sample processing, five fragments of tissue (organ) per animal were randomly selected for further analyses and the raw data obtained by *ex vivo* luciferase assay is represented in **(k, m**, and **o)**. The average Gaussia luciferase activity calculated for each mouse is represented in **(h-j**, **l**, **n**, and **p)**. One-Way ANOVA followed by Tukey's Multiple Comparison Test. *p<0.05, **p<0.01, ***p<0.001, ns: statistically non-significant.

**
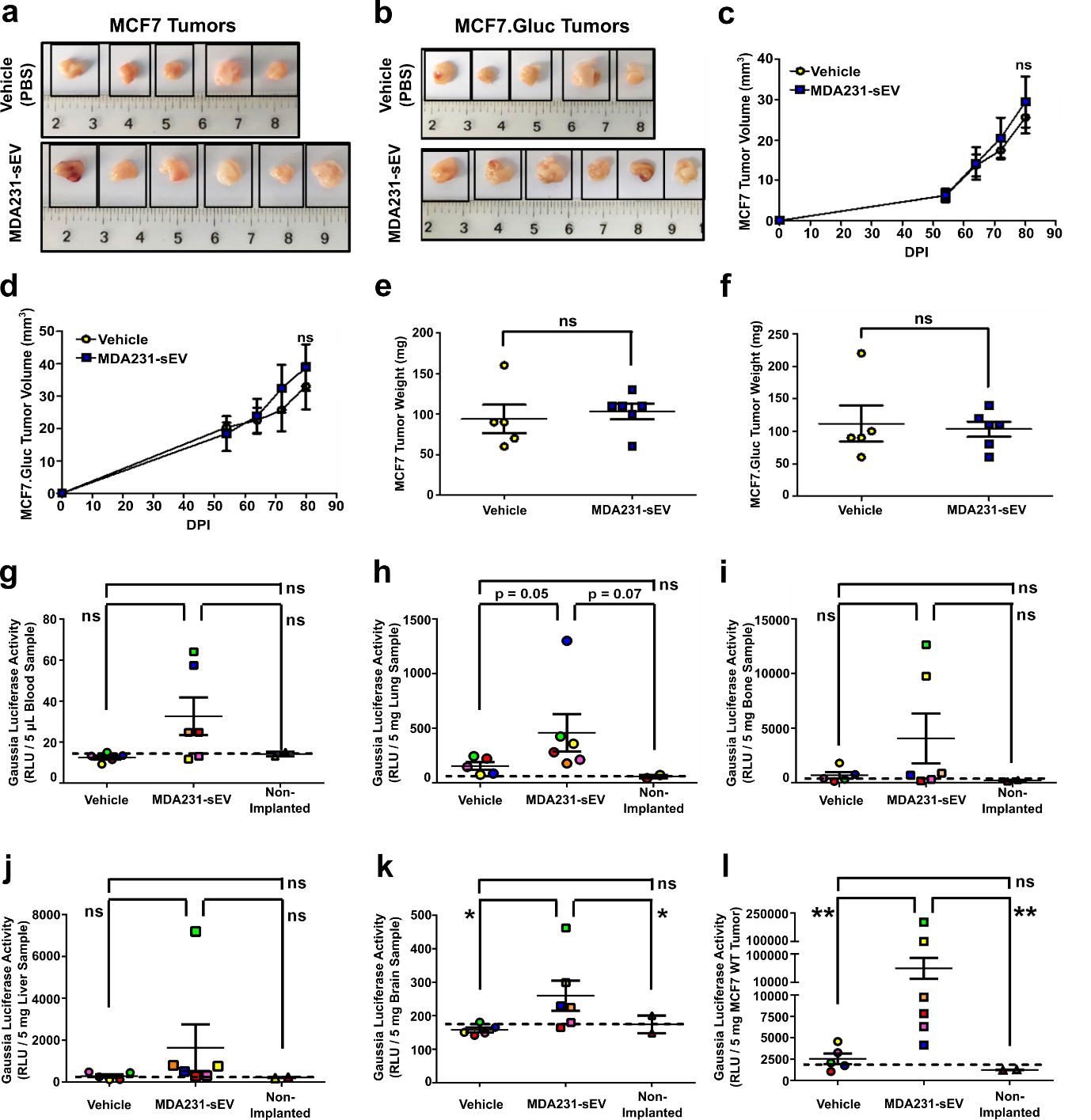
**

**Supplementary figure 11:** **Additional characterization for the progression of MCF7 and MCF7.Gluc tumors in animals treated with MDA231-sEVs, related to Figure 7.** **(a)** MCF7 and **(b)** MCF7.Gluc tumor-containing fat pads harvested from animals treated with MDA231-sEVs (or vehicle). The growth of **(c)** MCF7 and **(d)** MCF7.Gluc tumors was monitored until palpable and then measured with a caliper. Tumor weights for harvested **(e)** MCF7 and **(f)** MCF7.Gluc tumors. Results represent mean ± SEM. Unpaired Student’s t-test was used to analyze data in **(c-f)**. **(g-l)** Gaussia luciferase activity quantified by *ex vivo* luciferase assay in **(g)** blood, **(h)** lung, **(i)** bone, **(j)** liver, **(k)** brain, and **(l)** unlabeled MCF7 wild type tumor samples representing the presence of MCF7.Gluc cells (5-6 animals/group). Animals are color-coded. Black dashed lines indicate background activity for the Gaussia luciferase as quantified in samples from non-implanted mice (n = 2 mice). After sample processing, five fragments of tissue (organ) per animal were randomly selected for further analyses and the average Gaussia luciferase activity calculated for each mouse is represented. One-Way ANOVA followed by Tukey's Multiple Comparison Test. *p<0.05, **p<0.01, ns: statistically non-significant.

**
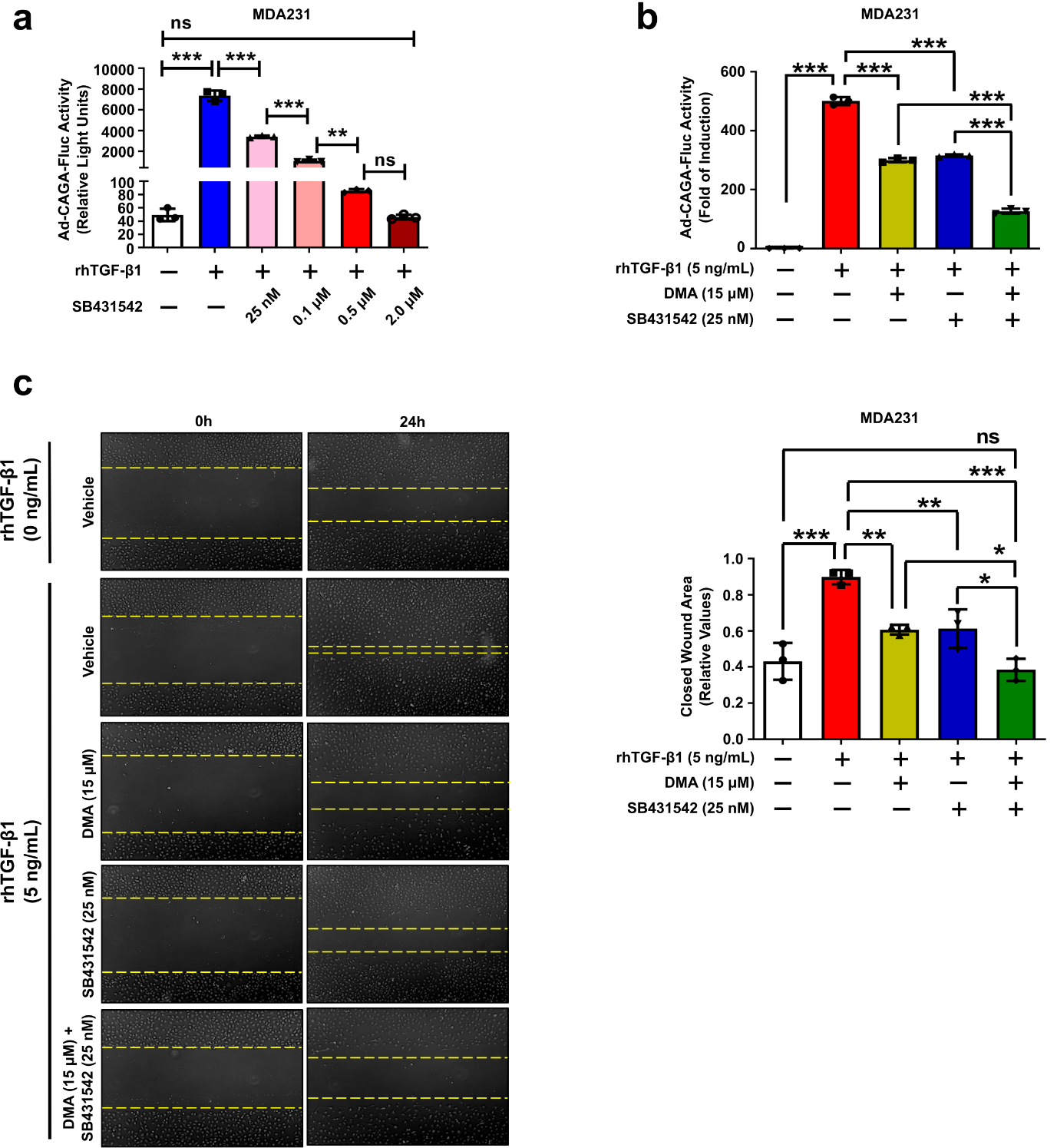
**

**Supplementary figure 12: Analysis of the combined use of DMA and SB431542 at suboptimal concentrations *in vitro*, related to Figure 8. (a)** The TGF-β/SMAD3 signaling reporter (Ad-CAGA-Fluc) activity was quantified in MDA231 cells treated ± SB431542 at increasing concentrations prior treatment with recombinant human (rh)TGF-β1 (5 ng/mL) for 24h. **(b)** Similarly, the TGF-β/SMAD3 signaling reporter (Ad-CAGA-Fluc) activity was quantified in MDA231 cells treated ± DMA ± SB431542 prior to stimulation with rhTGF-β1 for 24h. Results were normalized by Gaussia Luciferase (Ad-CMV-Gluc) activity. **(b)** MDA231 cell migration was evaluated by wound healing assay in cell cultures treated ± DMA and/or SB431542 before rhTGF-β1 treatment. DMSO was used as a vehicle for SB431542 and DMA. Results represent mean ± SD (n≥3). One-way ANOVA test followed by Dunn's Multiple Comparison test was used to analyze the obtained data**.** ns: statistically non-significant, *p < 0.05, **p < 0.01, ***p < 0.001.

**
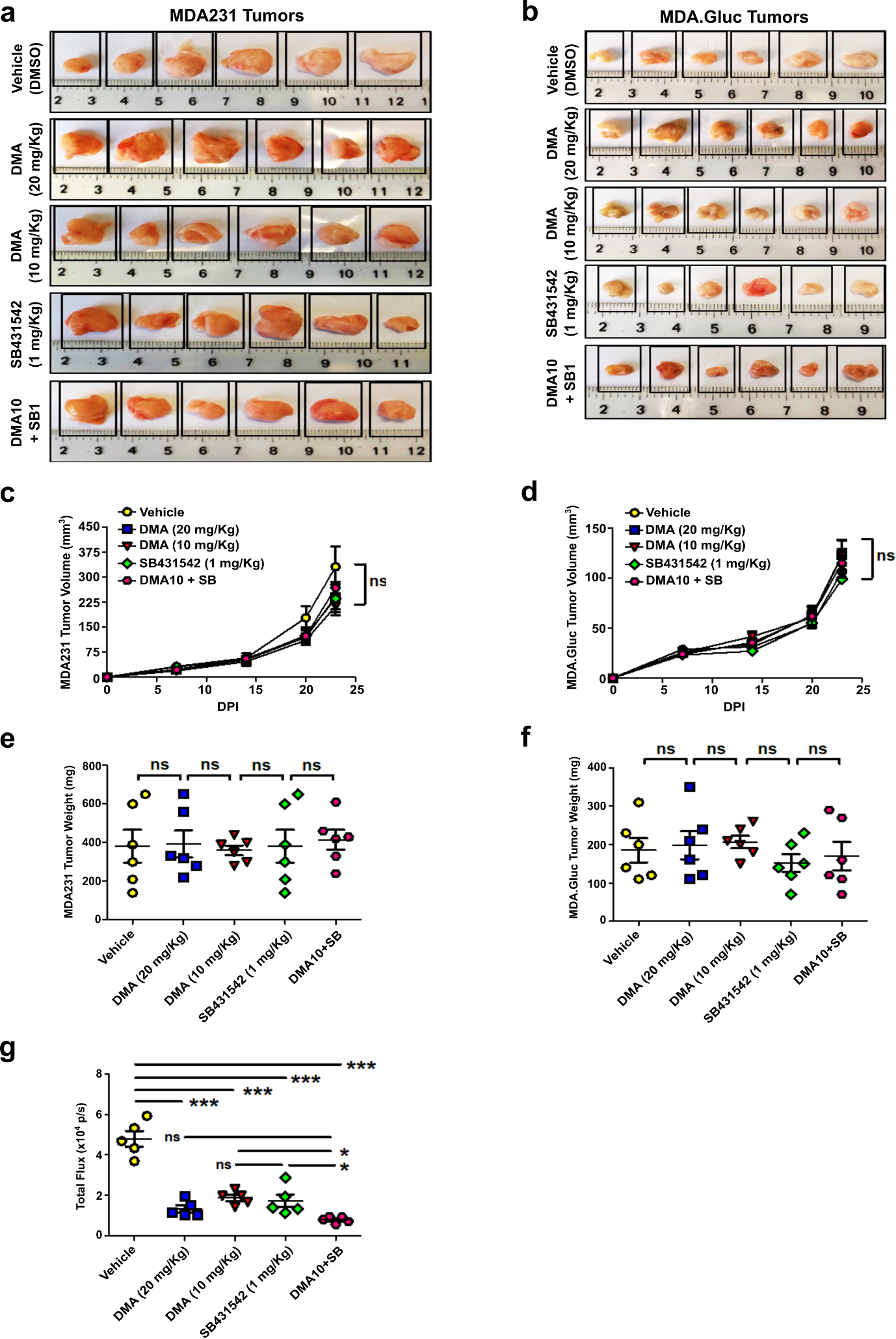
**

**Supplementary figure 13: Additional characterization for the progression of MDA231 and MDA.Gluc tumors in animals treated with DMA ± SB431542, related to Figure 8. (a)** MDA231 and **(b)** MDA.Gluc tumors harvested from animals injected with vehicle (DMSO), DMA and/or SB431542 at indicated concentrations. The growth of **(c)** MDA231 and **(d)** MDA.Gluc tumors was monitored until palpable and then measured with a caliper. Tumor weights for harvested **(e)** MDA231 and **(f)** MDA.Gluc tumors. **(g)** Quantification of bioluminescence emitted by MDA.Gluc tumors from mice treated with vehicle (DMSO), DMA (20 mg/Kg), DMA (10 mg/Kg), SB431542 (1 m/Kg), or a combined therapy with DMA (10 mg/Kg) + SB431542 (1 mg/Kg). One-Way ANOVA followed by Tukey's Multiple Comparison Test. *p<0.05, ***p<0.001, ns: statistically non-significant.

**
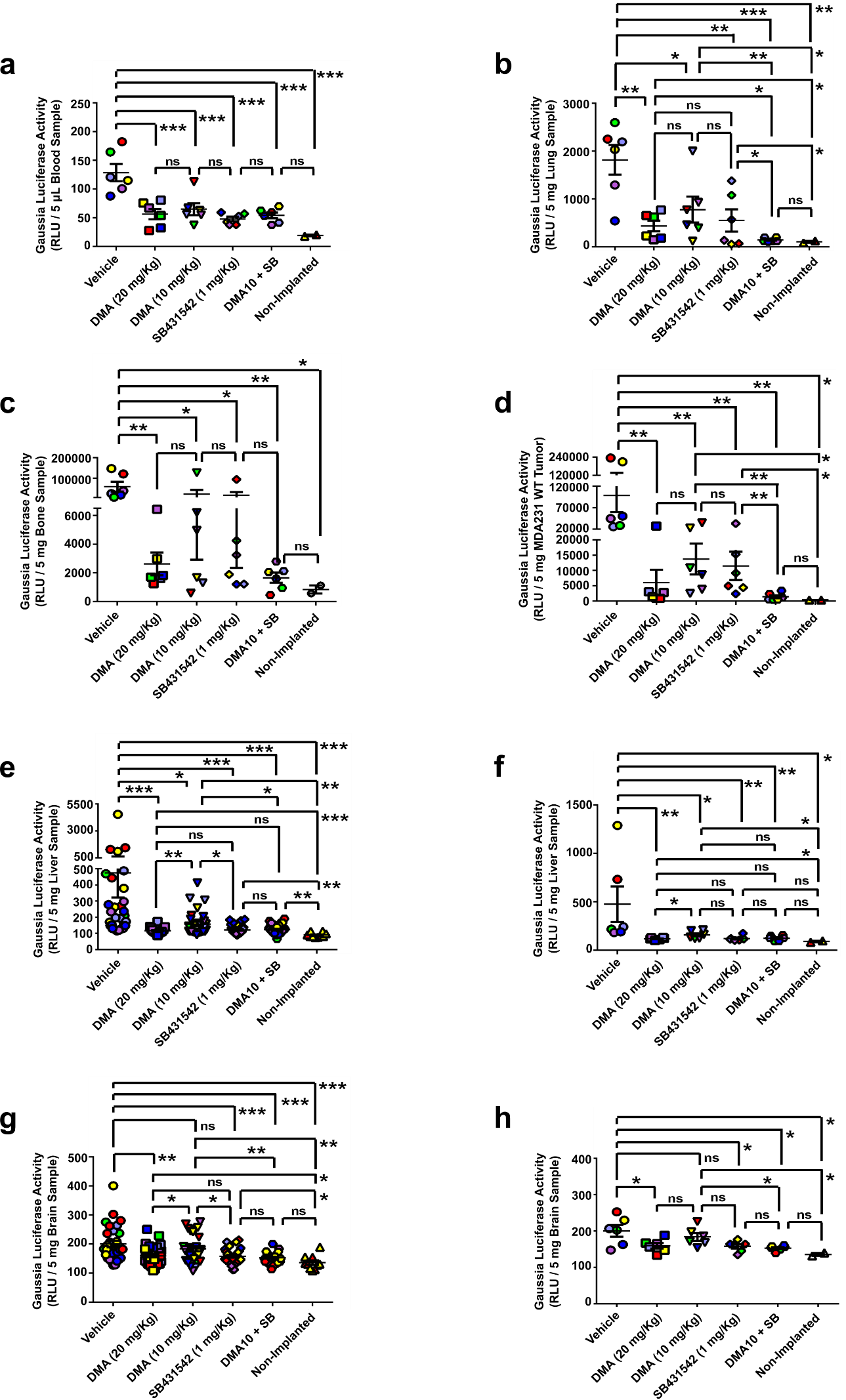
Supplementary figure 14: Quantification of the Gaussia luciferase activity corresponding to MDA.Gluc cells in the circulation and distant organs in mice treated with DMA ± SB431542, related to Figure 8.** Gaussia luciferase activity quantified by *ex vivo* luciferase assay in **(a)** blood samples, **(b)** lung samples, **(c)** bone samples, **(d)** unlabeled MDA231 wild type tumors, **(e-f)** liver samples, and **(g-h)** brain samples, representing the presence of MDA.Gluc cells (6 animals/group). Animals are color-coded. The background activity for the Gaussia luciferase was quantified in samples from non-implanted mice (n = 2 mice). After sample processing, five fragments of tissue (organ) per animal were randomly selected for further analyses and the raw data obtained by *ex vivo* luciferase assay is represented in **(e** and **g)**. The average Gaussia luciferase activity calculated for each mouse is represented in **(a-d**, **f**, and **h)**. One-Way ANOVA followed by Tukey's Multiple Comparison Test. *p<0.05, **p<0.01, ***p<0.001, ns: statistically non-significant.

**Supplementary table 1:** List of exosome-related genes.

| Gene | Ensembl ID | Chromosome | Biological Process |
| --- | --- | --- | --- |
| *ACAP2* | ENSG00000114331.11 | chr3 | Endosome Maturation |
| *ALS2* | ENSG00000003393.13 | chr2 | Endosome Maturation |
| *ANKFY1* | ENSG00000185722.15 | chr17 | Retromer Complex Activity |
| *APPL1* | ENSG00000157500.9 | chr3 | Endosome Maturation |
| *BECN1* | ENSG00000126581.11 | chr17 | Endosome Maturation |
| *CCDC53* | ENSG00000120860.9 | chr12 | Retromer Complex Activity |
| *CHMP1B* | ENSG00000255112.2 | chr18 | ESCRT Complex Activity |
| *CHMP2B* | ENSG00000083937.7 | chr3 | ESCRT Complex Activity |
| *CHMP3* | ENSG00000115561.13 | chr2 | ESCRT Complex Activity |
| *DENND1A* | ENSG00000119522.14 | chr9 | Endosome Maturation |
| *DENND1B* | ENSG00000213047.10 | chr1 | Endosome Maturation |
| *DENND1C* | ENSG00000205744.8 | chr19 | Endosome Maturation |
| *DENND2/ST5* | ENSG00000166444.16 | chr11 | Endosome Maturation |
| *DENND2A* | ENSG00000146966.11 | chr7 | Endosome Maturation |
| *DENND2C* | ENSG00000175984.13 | chr1 | Endosome Maturation |
| *DENND2D* | ENSG00000162777.15 | chr1 | Endosome Maturation |
| *DENND3* | ENSG00000105339.9 | chr8 | Endosome Maturation |
| *DENND4A* | ENSG00000174485.13 | chr15 | Endosome Maturation |
| *DENND4B* | ENSG00000198837.8 | chr1 | Endosome Maturation |
| *DENND4C* | ENSG00000137145.19 | chr9 | Endosome Maturation |
| *DNAJC13* | ENSG00000138246.14 | chr3 | Retromer Complex Activity |
| *EEA1* | ENSG00000102189.15 | chr12 | Endosome Maturation |
| *EVI5*^†^ | ENSG00000067208.13 | chr1 | Endosome Maturation |
| *EVI5L*^†^ | ENSG00000142459.7 | chr19 | Endosome Maturation |
| *FAM21A* | ENSG00000099290.14 | chr10 | Retromer Complex Activity |
| *FAM21C* | ENSG00000172661.16 | chr10 | Retromer Complex Activity |
| *FAM45A* | ENSG00000119979.14 | chr10 | Endosome Maturation |
| *FIG4* | ENSG00000112367.9 | chr6 | Endosome Maturation |
| *FLCN* | ENSG00000154803.11 | chr17 | Endosome Maturation |
| *GAPVD1* | ENSG00000165219.20 | chr9 | Endosome Maturation |
| *HSP90AA1* | ENSG00000080824.17 | chr14 | SNARE Complex Activity |
| *IST1* | ENSG00000182149.19 | chr16 | ESCRT Complex Activity |
| *KIAA0196/WASHC5* | ENSG00000164961.14 | chr8 | Retromer Complex Activity |
| *KIAA1033/WASHC4* | ENSG00000136051.12 | chr12 | Retromer Complex Activity |
| *LEPROT* | ENSG00000213625.7 | chr1 | Endosome Maturation |
| *LYST* | ENSG00000143669.12 | chr1 | ESCRT Complex Activity |
| *MON1A* | ENSG00000164077.12 | chr3 | Endosome Maturation |
| *MON1B* | ENSG00000103111.13 | chr16 | Endosome Maturation |
| *MYO5A* | ENSG00000197535.13 | chr15 | Endosome Maturation |
| *MYO5B* | ENSG00000167306.17 | chr18 | Endosome Maturation |
| *OCRL* | ENSG00000122126.14 | chrX | Endosome Maturation |

^†^These genes are reported to inhibit exosome biogenesis and/or secretion and were included in the signature for exosome-related genes with a negative weight.

**Supplementary table 1:** List of exosome-related genes (continued).

| Gene | Ensembl ID | Chromosome | Biological Process |
| --- | --- | --- | --- |
| *PARK2* | ENSG00000185345.17 | chr6 | ESCRT Complex Activity |
| *PDCD6IP* | ENSG00000170248.12 | chr3 | ESCRT Complex Activity |
| *PIK3C3* | ENSG00000078142.10 | chr18 | Endosome Maturation |
| *PIK3CB* | ENSG00000051382.7 | chr3 | Endosome Maturation |
| *PIK3R4* | ENSG00000196455.6 | chr3 | Endosome Maturation |
| *PIKFYVE* | ENSG00000115020.15 | chr2 | Endosome Maturation |
| *PLEKHM1* | ENSG00000214176.8 | chr17 | Endosome Maturation |
| *RAB10* | ENSG00000084733.9 | chr2 | Endosome Maturation |
| *RAB11A* | ENSG00000103769.8 | chr15 | Endosome Maturation |
| *RAB11B* | ENSG00000185236.10 | chr19 | Endosome Maturation |
| *RAB12* | ENSG00000206418.3 | chr18 | Endosome Maturation |
| *RAB14* | ENSG00000119396.9 | chr9 | Endosome Maturation |
| *RAB21* | ENSG00000080371.5 | chr12 | Endosome Maturation |
| *RAB27A* | ENSG00000069974.14 | chr15 | Endosome Maturation |
| *RAB27B* | ENSG00000041353.8 | chr18 | Endosome Maturation |
| *RAB28* | ENSG00000157869.13 | chr4 | Endosome Maturation |
| *RAB2A* | ENSG00000104388.13 | chr8 | Endosome Maturation |
| *RAB2B* | ENSG00000129472.11 | chr14 | Endosome Maturation |
| *RAB33A* | ENSG00000134594.4 | chrX | Endosome Maturation |
| *RAB33B* | ENSG00000172007.5 | chr4 | Endosome Maturation |
| *RAB35* | ENSG00000111737.10 | chr12 | Endosome Maturation |
| *RAB5A* | ENSG00000144566.9 | chr3 | Endosome Maturation |
| *RAB5B* | ENSG00000111540.14 | chr12 | Endosome Maturation |
| *RAB5C* | ENSG00000108774.13 | chr17 | Endosome Maturation |
| *RAB6A* | ENSG00000175582.18 | chr11 | Endosome Maturation |
| *RAB6B* | ENSG00000154917.9 | chr3 | Endosome Maturation |
| *RAB6C* | ENSG00000222014.5 | chr2 | Endosome Maturation |
| *RAB7A* | ENSG00000075785.11 | chr3 | Endosome Maturation |
| *RAB9A* | ENSG00000123595.6 | chrX | Endosome Maturation |
| *RAB9B* | ENSG00000123570.3 | chrX | Endosome Maturation |
| *RABEP1* | ENSG00000029725.15 | chr17 | Endosome Maturation |
| *RABGEF1* | ENSG00000154710.14 | chr7 | Endosome Maturation |
| *RALA* | ENSG00000006451.6 | chr7 | Endosome Maturation |
| *RALB* | ENSG00000144118.12 | chr2 | Endosome Maturation |
| *RILP* | ENSG00000167705.10 | chr17 | Endosome Maturation |
| *RIN1* | ENSG00000174791.9 | chr11 | Endosome Maturation |
| *RIN2* | ENSG00000132669.11 | chr20 | Endosome Maturation |
| *RUBCN/KIAA0226*^†^ | ENSG00000145016.12 | chr3 | Endosome Maturation |
| *RUSC1* | ENSG00000160753.14 | chr1 | Endosome Maturation |
| *RUSC2* | ENSG00000198853.10 | chr9 | Endosome Maturation |
| *SBF1* | ENSG00000100241.19 | chr22 | Endosome Maturation |

^†^These genes are reported to inhibit exosome biogenesis and/or secretion and were included in the signature for exosome-related genes with a negative weight.

**Supplementary table 1:** List of exosome-related genes (continued).

| Gene | Ensembl ID | Chromosome | Biological Process |
| --- | --- | --- | --- |
| *SBF2* | ENSG00000133812.13 | chr11 | Endosome Maturation |
| *SDCBP* | ENSG00000137575.10 | chr8 | ESCRT Complex Activity |
| *SGSM3*^†^ | ENSG00000100359.19 | chr22 | Endosome Maturation |
| *SLAC2A/MLPH* | ENSG00000115648.12 | chr2 | Endosome Maturation |
| *SLAC2B/EXPH5* | ENSG00000110723.10 | chr11 | Endosome Maturation |
| *SNX1* | ENSG00000028528.13 | chr15 | Retromer Complex Activity |
| *SNX2* | ENSG00000205302.5 | chr5 | Retromer Complex Activity |
| *SNX3* | ENSG00000112335.13 | chr6 | Retromer Complex Activity |
| *SNX5* | ENSG00000089006.15 | chr20 | Retromer Complex Activity |
| *SNX6* | ENSG00000129515.17 | chr14 | Retromer Complex Activity |
| *STAM* | ENSG00000136738.13 | chr10 | ESCRT Complex Activity |
| *STIP1* | ENSG00000168439.15 | chr11 | SNARE Complex Activity |
| *STX12* | ENSG00000117758.12 | chr1 | SNARE Complex Activity |
| *STX1A* | ENSG00000106089.10 | chr7 | SNARE Complex Activity |
| *STX2* | ENSG00000111450.12 | chr12 | SNARE Complex Activity |
| *STX7* | ENSG00000079950.12 | chr6 | SNARE Complex Activity |
| *STXBP1* | ENSG00000136854.16 | chr9 | SNARE Complex Activity |
| *STXBP5* | ENSG00000164506.13 | chr6 | SNARE Complex Activity |
| *SYT7* | ENSG00000011347.8 | chr11 | SNARE Complex Activity |
| *SYTL4* | ENSG00000102362.14 | chrX | Endosome Maturation |
| *TBC1D1*^†^ | ENSG00000065882.14 | chr4 | Endosome Maturation |
| *TBC1D10A*^†^ | ENSG00000099992.14 | chr22 | Endosome Maturation |
| *TBC1D10B*^†^ | ENSG00000169221.12 | chr16 | Endosome Maturation |
| *TBC1D10C*^†^ | ENSG00000175463.10 | chr11 | Endosome Maturation |
| *TBC1D13*^†^ | ENSG00000107021.14 | chr9 | Endosome Maturation |
| *TBC1D15*^†^ | ENSG00000121749.14 | chr12 | Endosome Maturation |
| *TBC1D16*^†^ | ENSG00000167291.14 | chr17 | Endosome Maturation |
| *TBC1D17*^†^ | ENSG00000104946.9 | chr19 | Endosome Maturation |
| *TBC1D18/RABGAP1L*^†^ | ENSG00000152061.20 | chr1 | Endosome Maturation |
| *TBC1D2*^†^ | ENSG00000095383.18 | chr9 | Endosome Maturation |
| *TBC1D20*^†^ | ENSG00000125875.12 | chr20 | Endosome Maturation |
| *TBC1D23*^†^ | ENSG00000036054.11 | chr3 | Endosome Maturation |
| *TBC1D24*^†^ | ENSG00000162065.10 | chr16 | Endosome Maturation |
| *TBC1D25*^†^ | ENSG00000068354.14 | chrX | Endosome Maturation |
| *TBC1D3*^†^ | ENSG00000274419.3 | chr17 | Endosome Maturation |
| *TBC1D3B*^†^ | ENSG00000274808.3 | chr17 | Endosome Maturation |
| *TBC1D3C*^†^ | ENSG00000278299.3 | chr17 | Endosome Maturation |
| *TBC1D4*^†^ | ENSG00000136111.11 | chr13 | Endosome Maturation |
| *TBC1D5*^†^ | ENSG00000131374.13 | chr3 | Endosome Maturation |
| *TBC1D6/GRTP1*^†^ | ENSG00000139835.12 | chr13 | Endosome Maturation |
| *TBC1D7*^†^ | ENSG00000145979.16 | chr6 | Endosome Maturation |

^†^These genes are reported to inhibit exosome biogenesis and/or secretion and were included in the signature for exosome-related genes with a negative weight.

**Supplementary table 1:** List of exosome-related genes (continued).

| Gene | Ensembl ID | Chromosome | Biological Process |
| --- | --- | --- | --- |
| *TGFBRAP1* | ENSG00000135966.11 | chr2 | Endosome Maturation |
| *TSG101* | ENSG00000074319.11 | chr11 | ESCRT Complex Activity |
| *USP6*^†^ | ENSG00000129204.15 | chr17 | Endosome Maturation |
| *USP6NL*^†^ | ENSG00000148429.13 | chr10 | Endosome Maturation |
| *UVRAG* | ENSG00000198382.7 | chr11 | Endosome Maturation |
| *VAMP2* | ENSG00000220205.7 | chr17 | SNARE Complex Activity |
| *VAMP3* | ENSG00000049245.11 | chr1 | SNARE Complex Activity |
| *VPS26A* | ENSG00000122958.13 | chr10 | Retromer Complex Activity |
| *VPS26B* | ENSG00000151502.9 | chr11 | Retromer Complex Activity |
| *VPS29* | ENSG00000111237.17 | chr12 | Retromer Complex Activity |
| *VPS35* | ENSG00000069329.14 | chr16 | Retromer Complex Activity |
| *VPS8* | ENSG00000156931.14 | chr3 | Endosome Maturation |
| *VTA1* | ENSG00000009844.14 | chr6 | ESCRT Complex Activity |
| *ZFYVE20/RBSN* | ENSG00000131381.11 | chr3 | Endosome Maturation |

^†^These genes are reported to inhibit exosome biogenesis and/or secretion and were included in the signature for exosome-related genes with a negative weight.

**Supplementary table 2:** Exosome-related genes individually associated with the overall survival of breast cancer patients (TCGA BRCA cohort, n = 1,214).

| Gene | Reduced OS | Analyzed | p-value |
| --- | --- | --- | --- |
| *ACAP2* | High Expression | 2 groups | p < 0.001 |
| *ALS2* | High Expression | 2 groups | p < 0.05 |
| *ANKFY1* | High Expression | 2 groups | p < 0.01 |
| *APPL1* | Not Correlated | 2 groups/Quartiles | p > 0.05 |
| *BECN1* | Not Correlated | 2 groups/Quartiles | p > 0.05 |
| *CCDC53* | Not Correlated | 2 groups/Quartiles | p > 0.05 |
| *CHMP1B* | Not Correlated | 2 groups/Quartiles | p > 0.05 |
| *CHMP2B* | High Expression | 2 groups | p < 0.05 |
| *CHMP3* | High Expression | 2 groups | p < 0.01 |
| *DENND1A* | Not Correlated | 2 groups/Quartiles | p > 0.05 |
| *DENND1B* | Not Correlated | 2 groups/Quartiles | p > 0.05 |
| *DENND1C* | Low Expression | 2 groups | p < 0.05 |
| *DENND2/ST5* | Not Correlated | 2 groups/Quartiles | p > 0.05 |
| *DENND2A* | High Expression | 2 groups | p < 0.05 |
| *DENND2C* | High Expression | 2 groups | p < 0.05 |
| *DENND2D* | Low Expression | 2 groups | p < 0.001 |
| *DENND3* | Not Correlated | 2 groups/Quartiles | p > 0.05 |
| *DENND4A* | High Expression | 2 groups | p < 0.05 |
| *DENND4B* | Not Correlated | 2 groups/Quartiles | p > 0.05 |
| *DENND4C* | High Expression | 2 groups | p < 0.05 |
| *DNAJC13* | High Expression | 2 groups | p < 0.001 |
| *EEA1* | High Expression | 2 groups | p < 0.01 |
| *EVI5* | Not Correlated | 2 groups/Quartiles | p > 0.05 |
| *EVI5L* | Not Correlated | 2 groups/Quartiles | p > 0.05 |
| *FAM21A* | Not Correlated | 2 groups/Quartiles | p > 0.05 |
| *FAM21C* | High Expression | 2 groups | p < 0.05 |
| *FAM45A* | High Expression | Quartiles | p < 0.05 |
| *FIG4* | High Expression | 2 groups | p < 0.01 |
| *FLCN* | High Expression | Quartiles | p < 0.01 |
| *GAPVD1* | High Expression | 2 groups | p < 0.05 |
| *HSP90AA1* | Not Correlated | 2 groups/Quartiles | p > 0.05 |
| *IST1* | Not Correlated | 2 groups/Quartiles | p > 0.05 |
| *KIAA0196/WASHC5* | Not Correlated | 2 groups/Quartiles | p > 0.05 |
| *KIAA1033/WASHC4* | High Expression | 2 groups | p < 0.05 |
| *LEPROT* | High Expression | Quartiles | p < 0.01 |
| *LYST* | Not Correlated | 2 groups/Quartiles | p > 0.05 |
| *MON1A* | Low Expression | 2 groups | p < 0.05 |
| *MON1B* | High Expression | 2 groups | p < 0.05 |
| *MYO5A* | High Expression | 2 groups | p < 0.05 |
| *MYO5B* | Low Expression | 2 groups | p < 0.01 |
| *OCRL* | High Expression | Quartiles | p < 0.01 |
| *PARK2* | High Expression | Quartiles | p < 0.001 |

**Supplementary table 2:** Exosome-related genes individually associated with the overall survival of breast cancer patients (TCGA BRCA cohort, n = 1,214).

| Gene | Reduced OS | Analyzed | p-value |
| --- | --- | --- | --- |
| *PDCD6IP* | Not Correlated | 2 groups/Quartiles | p > 0.05 |
| *PIK3C3* | High Expression | 2 groups | p < 0.05 |
| *PIK3CB* | High Expression | Quartiles | p < 0.05 |
| *PIK3R4* | High Expression | 2 groups | p < 0.05 |
| *PIKFYVE* | High Expression | 2 groups | p < 0.01 |
| *PLEKHM1* | High Expression | 2 groups | p < 0.05 |
| *RAB10* | Not Correlated | 2 groups/Quartiles | p > 0.05 |
| *RAB11A* | Not Correlated | 2 groups/Quartiles | p > 0.05 |
| *RAB11B* | Not Correlated | 2 groups/Quartiles | p > 0.05 |
| *RAB12* | High Expression | 2 groups | p < 0.05 |
| *RAB14* | High Expression | Quartiles | p < 0.01 |
| *RAB21* | High Expression | 2 groups | p < 0.001 |
| *RAB27A* | Not Correlated | 2 groups/Quartiles | p > 0.05 |
| *RAB27B* | Not Correlated | 2 groups/Quartiles | p > 0.05 |
| *RAB28* | High Expression | 2 groups | p < 0.01 |
| *RAB2A* | High Expression | 2 groups | p < 0.05 |
| *RAB2B* | High Expression | 2 groups | p < 0.01 |
| *RAB33A* | Low Expression | 2 groups | p < 0.05 |
| *RAB33B* | High Expression | 2 groups | p < 0.01 |
| *RAB35* | Not Correlated | 2 groups/Quartiles | p > 0.05 |
| *RAB5A* | High Expression | 2 groups | p < 0.01 |
| *RAB5B* | Not Correlated | 2 groups/Quartiles | p > 0.05 |
| *RAB5C* | Not Correlated | 2 groups/Quartiles | p > 0.05 |
| *RAB6A* | Not Correlated | 2 groups/Quartiles | p > 0.05 |
| *RAB6B* | High Expression | 2 groups | p < 0.01 |
| *RAB6C* | Not Correlated | 2 groups/Quartiles | p > 0.05 |
| *RAB7A* | Not Correlated | 2 groups/Quartiles | p > 0.05 |
| *RAB9A* | High Expression | 2 groups | p < 0.05 |
| *RAB9B* | High Expression | 2 groups | p < 0.01 |
| *RABEP1* | Not Correlated | 2 groups/Quartiles | p > 0.05 |
| *RABGEF1* | Not Correlated | 2 groups/Quartiles | p > 0.05 |
| *RALA* | Not Correlated | 2 groups/Quartiles | p > 0.05 |
| *RALB* | High Expression | Quartiles | p < 0.05 |
| *RILP* | Not Correlated | 2 groups/Quartiles | p > 0.05 |
| *RIN1* | High Expression | 2 groups | p < 0.05 |
| *RIN2* | High Expression | 2 groups | p < 0.01 |
| *RUBCN/KIAA0226* | Not Correlated | 2 groups/Quartiles | p > 0.05 |
| *RUSC1* | Not Correlated | 2 groups/Quartiles | p > 0.05 |
| *RUSC2* | Not Correlated | 2 groups/Quartiles | p > 0.05 |
| *SBF1* | Not Correlated | 2 groups/Quartiles | p > 0.05 |
| *SBF2* | High Expression | Quartiles | p < 0.05 |
| *SDCBP* | High Expression | 2 groups | p < 0.001 |

**Supplementary table 2:** Exosome-related genes individually associated with the overall survival of breast cancer patients (TCGA BRCA cohort, n = 1,214).

| Gene | Reduced OS | Analyzed | p-value |
| --- | --- | --- | --- |
| *SGSM3* | Not Correlated | 2 groups/Quartiles | p > 0.05 |
| *SLAC2A/MLPH* | Low Expression | 2 groups | p < 0.001 |
| *SLAC2B/EXPH5* | Not Correlated | 2 groups/Quartiles | p > 0.05 |
| *SNX1* | Not Correlated | 2 groups/Quartiles | p > 0.05 |
| *SNX2* | High Expression | 2 groups | p < 0.01 |
| *SNX3* | High Expression | 2 groups | p < 0.001 |
| *SNX5* | Not Correlated | 2 groups/Quartiles | p > 0.05 |
| *SNX6* | Not Correlated | 2 groups/Quartiles | p > 0.05 |
| *STAM* | Not Correlated | 2 groups/Quartiles | p > 0.05 |
| *STIP1* | Not Correlated | 2 groups/Quartiles | p > 0.05 |
| *STX12* | Not Correlated | 2 groups/Quartiles | p > 0.05 |
| *STX1A* | Not Correlated | 2 groups/Quartiles | p > 0.05 |
| *STX2* | High Expression | 2 groups | p < 0.05 |
| *STX7* | High Expression | 2 groups | p < 0.01 |
| *STXBP1* | High Expression | 2 groups | p < 0.01 |
| *STXBP5* | High Expression | 2 groups | p < 0.001 |
| *SYT7* | Not Correlated | 2 groups/Quartiles | p > 0.05 |
| *SYTL4* | Not Correlated | 2 groups/Quartiles | p > 0.05 |
| *TBC1D1* | Not Correlated | 2 groups/Quartiles | p > 0.05 |
| *TBC1D10A* | Not Correlated | 2 groups/Quartiles | p > 0.05 |
| *TBC1D10B* | Not Correlated | 2 groups/Quartiles | p > 0.05 |
| *TBC1D10C* | Low Expression | 2 groups | p < 0.01 |
| *TBC1D13* | High Expression | Quartiles | p < 0.05 |
| *TBC1D15* | High Expression | Quartiles | p < 0.05 |
| *TBC1D16* | Not Correlated | 2 groups/Quartiles | p > 0.05 |
| *TBC1D17* | Not Correlated | 2 groups/Quartiles | p > 0.05 |
| *TBC1D18/RABGAP1L* | Not Correlated | 2 groups/Quartiles | p > 0.05 |
| *TBC1D2* | Not Correlated | 2 groups/Quartiles | p > 0.05 |
| *TBC1D20* | High Expression | 2 groups | p < 0.01 |
| *TBC1D23* | High Expression | 2 groups | p < 0.01 |
| *TBC1D24* | Not Correlated | 2 groups/Quartiles | p > 0.05 |
| *TBC1D25* | High Expression | Quartiles | p < 0.01 |
| *TBC1D3* | Not Correlated | 2 groups/Quartiles | p > 0.05 |
| *TBC1D3B* | Low Expression | 2 groups | p < 0.05 |
| *TBC1D3C* | Not Correlated | 2 groups/Quartiles | p > 0.05 |
| *TBC1D4* | Not Correlated | 2 groups/Quartiles | p > 0.05 |
| *TBC1D5* | High Expression | 2 groups | p < 0.05 |
| *TBC1D6/GRTP1* | Low Expression | 2 groups | p < 0.05 |
| *TBC1D7* | Not Correlated | 2 groups/Quartiles | p > 0.05 |
| *TGFBRAP1* | Not Correlated | 2 groups/Quartiles | p > 0.05 |
| *TSG101* | Low Expression | Quartiles | p < 0.05 |
| *USP6* | Not Correlated | 2 groups/Quartiles | p > 0.05 |

**Supplementary table 2:** Exosome-related genes individually associated with the overall survival of breast cancer patients (TCGA BRCA cohort, n = 1,214).

| Gene | Reduced OS | Analyzed | p-value |
| --- | --- | --- | --- |
| *USP6NL* | Not Correlated | 2 groups/Quartiles | p > 0.05 |
| *UVRAG* | Not Correlated | 2 groups/Quartiles | p > 0.05 |
| *VAMP2* | High Expression | 2 groups | p < 0.001 |
| *VAMP3* | High Expression | 2 groups | p < 0.01 |
| *VPS26A* | High Expression | Quartiles | p < 0.05 |
| *VPS26B* | Not Correlated | 2 groups/Quartiles | p > 0.05 |
| *VPS29* | Not Correlated | 2 groups/Quartiles | p > 0.05 |
| *VPS35* | Not Correlated | 2 groups/Quartiles | p > 0.05 |
| *VPS8* | Not Correlated | 2 groups/Quartiles | p > 0.05 |
| *VTA1* | High Expression | Quartiles | p < 0.05 |
| *ZFYVE20/RBSN* | High Expression | 2 groups | p < 0.05 |

**Supplementary table 3:** Exosome-related genes individually associated with the disease-free interval of breast cancer patients (TCGA BRCA cohort, n = 1,037).

| Gene | Reduced DFI | Analyzed | p-value |
| --- | --- | --- | --- |
| *ACAP2* | Not Correlated | 2 groups/Quartiles | p > 0.05 |
| *ALS2* | Not Correlated | 2 groups/Quartiles | p > 0.05 |
| *ANKFY1* | High Expression | Quartiles | p < 0.05 |
| *APPL1* | Low Expression | 2 groups | p < 0.01 |
| *BECN1* | Low Expression | 2 groups | p < 0.01 |
| *CCDC53* | Not Correlated | 2 groups/Quartiles | p > 0.05 |
| *CHMP1B* | Not Correlated | 2 groups/Quartiles | p > 0.05 |
| *CHMP2B* | Not Correlated | 2 groups/Quartiles | p > 0.05 |
| *CHMP3* | Not Correlated | 2 groups/Quartiles | p > 0.05 |
| *DENND1A* | Not Correlated | 2 groups/Quartiles | p > 0.05 |
| *DENND1B* | Not Correlated | 2 groups/Quartiles | p > 0.05 |
| *DENND1C* | Not Correlated | 2 groups/Quartiles | p > 0.05 |
| *DENND2/ST5* | Not Correlated | 2 groups/Quartiles | p > 0.05 |
| *DENND2A* | Not Correlated | 2 groups/Quartiles | p > 0.05 |
| *DENND2C* | Not Correlated | 2 groups/Quartiles | p > 0.05 |
| *DENND2D* | Low Expression | Quartiles | p < 0.05 |
| *DENND3* | Not Correlated | 2 groups/Quartiles | p > 0.05 |
| *DENND4A* | Not Correlated | 2 groups/Quartiles | p > 0.05 |
| *DENND4B* | High Expression | 2 groups | p < 0.05 |
| *DENND4C* | Not Correlated | 2 groups/Quartiles | p > 0.05 |
| *DNAJC13* | Not Correlated | 2 groups/Quartiles | p > 0.05 |
| *EEA1* | Not Correlated | 2 groups/Quartiles | p > 0.05 |
| *EVI5* | Not Correlated | 2 groups/Quartiles | p > 0.05 |
| *EVI5L* | High Expression | 2 groups | p < 0.05 |
| *FAM21A* | Not Correlated | 2 groups/Quartiles | p > 0.05 |
| *FAM21C* | Not Correlated | 2 groups/Quartiles | p > 0.05 |
| *FAM45A* | Low Expression | 2 groups | p < 0.05 |
| *FIG4* | Not Correlated | 2 groups/Quartiles | p > 0.05 |
| *FLCN* | Not Correlated | 2 groups/Quartiles | p > 0.05 |
| *GAPVD1* | Not Correlated | 2 groups/Quartiles | p > 0.05 |
| *HSP90AA1* | High Expression | 2 groups | p < 0.05 |
| *IST1* | Not Correlated | 2 groups/Quartiles | p > 0.05 |
| *KIAA0196/WASHC5* | Not Correlated | 2 groups/Quartiles | p > 0.05 |
| *KIAA1033/WASHC4* | Not Correlated | 2 groups/Quartiles | p > 0.05 |
| *LEPROT* | Not Correlated | 2 groups/Quartiles | p > 0.05 |
| *LYST* | Low Expression | 2 groups | p < 0.01 |
| *MON1A* | Not Correlated | 2 groups/Quartiles | p > 0.05 |
| *MON1B* | Not Correlated | 2 groups/Quartiles | p > 0.05 |
| *MYO5A* | Not Correlated | 2 groups/Quartiles | p > 0.05 |
| *MYO5B* | Low Expression | 2 groups | p < 0.01 |
| *OCRL* | Not Correlated | 2 groups/Quartiles | p > 0.05 |
| *PARK2* | Not Correlated | 2 groups/Quartiles | p > 0.05 |

**Supplementary table 3:** Exosome-related genes individually associated with the disease-free interval of breast cancer patients (TCGA BRCA cohort, n = 1,037).

| Gene | Reduced DFI | Analyzed | p-value |
| --- | --- | --- | --- |
| *PDCD6IP* | Not Correlated | 2 groups/Quartiles | p > 0.05 |
| *PIK3C3* | Not Correlated | 2 groups/Quartiles | p > 0.05 |
| *PIK3CB* | Not Correlated | 2 groups/Quartiles | p > 0.05 |
| *PIK3R4* | High Expression | Quartiles | p < 0.05 |
| *PIKFYVE* | Not Correlated | 2 groups/Quartiles | p > 0.05 |
| *PLEKHM1* | Not Correlated | 2 groups/Quartiles | p > 0.05 |
| *RAB10* | Not Correlated | 2 groups/Quartiles | p > 0.05 |
| *RAB11A* | Not Correlated | 2 groups/Quartiles | p > 0.05 |
| *RAB11B* | Not Correlated | 2 groups/Quartiles | p > 0.05 |
| *RAB12* | High Expression | 2 groups | p < 0.05 |
| *RAB14* | Not Correlated | 2 groups/Quartiles | p > 0.05 |
| *RAB21* | Not Correlated | 2 groups/Quartiles | p > 0.05 |
| *RAB27A* | Low Expression | 2 groups | p < 0.001 |
| *RAB27B* | Not Correlated | 2 groups/Quartiles | p > 0.05 |
| *RAB28* | Not Correlated | 2 groups/Quartiles | p > 0.05 |
| *RAB2A* | High Expression | 2 groups | p < 0.05 |
| *RAB2B* | Not Correlated | 2 groups/Quartiles | p > 0.05 |
| *RAB33A* | Not Correlated | 2 groups/Quartiles | p > 0.05 |
| *RAB33B* | Not Correlated | 2 groups/Quartiles | p > 0.05 |
| *RAB35* | Not Correlated | 2 groups/Quartiles | p > 0.05 |
| *RAB5A* | Not Correlated | 2 groups/Quartiles | p > 0.05 |
| *RAB5B* | Not Correlated | 2 groups/Quartiles | p > 0.05 |
| *RAB5C* | Not Correlated | 2 groups/Quartiles | p > 0.05 |
| *RAB6A* | Not Correlated | 2 groups/Quartiles | p > 0.05 |
| *RAB6B* | High Expression | 2 groups | p < 0.05 |
| *RAB6C* | Low Expression | 2 groups | p < 0.01 |
| *RAB7A* | High Expression | 2 groups | p < 0.05 |
| *RAB9A* | Not Correlated | 2 groups/Quartiles | p > 0.05 |
| *RAB9B* | Not Correlated | 2 groups/Quartiles | p > 0.05 |
| *RABEP1* | High Expression | 2 groups | p < 0.05 |
| *RABGEF1* | Not Correlated | 2 groups/Quartiles | p > 0.05 |
| *RALA* | Not Correlated | 2 groups/Quartiles | p > 0.05 |
| *RALB* | Not Correlated | 2 groups/Quartiles | p > 0.05 |
| *RILP* | Not Correlated | 2 groups/Quartiles | p > 0.05 |
| *RIN1* | Not Correlated | 2 groups/Quartiles | p > 0.05 |
| *RIN2* | Not Correlated | 2 groups/Quartiles | p > 0.05 |
| *RUBCN/KIAA0226* | Not Correlated | 2 groups/Quartiles | p > 0.05 |
| *RUSC1* | Not Correlated | 2 groups/Quartiles | p > 0.05 |
| *RUSC2* | Not Correlated | 2 groups/Quartiles | p > 0.05 |
| *SBF1* | Not Correlated | 2 groups/Quartiles | p > 0.05 |
| *SBF2* | Not Correlated | 2 groups/Quartiles | p > 0.05 |
| *SDCBP* | Not Correlated | 2 groups/Quartiles | p > 0.05 |

**Supplementary table 3:** Exosome-related genes individually associated with the disease-free interval of breast cancer patients (TCGA BRCA cohort, n = 1,037).

| Gene | Reduced DFI | Analyzed | p-value |
| --- | --- | --- | --- |
| *SGSM3* | Not Correlated | 2 groups/Quartiles | p > 0.05 |
| *SLAC2A/MLPH* | Low Expression | Quartiles | p < 0.05 |
| *SLAC2B/EXPH5* | Not Correlated | 2 groups/Quartiles | p > 0.05 |
| *SNX1* | Not Correlated | 2 groups/Quartiles | p > 0.05 |
| *SNX2* | Low Expression | Quartiles | p < 0.05 |
| *SNX3* | High Expression | 2 groups | p < 0.05 |
| *SNX5* | Not Correlated | 2 groups/Quartiles | p > 0.05 |
| *SNX6* | Not Correlated | 2 groups/Quartiles | p > 0.05 |
| *STAM* | Not Correlated | 2 groups/Quartiles | p > 0.05 |
| *STIP1* | Low Expression | 2 groups | p < 0.05 |
| *STX12* | Not Correlated | 2 groups/Quartiles | p > 0.05 |
| *STX1A* | Not Correlated | 2 groups/Quartiles | p > 0.05 |
| *STX2* | Not Correlated | 2 groups/Quartiles | p > 0.05 |
| *STX7* | Not Correlated | 2 groups/Quartiles | p > 0.05 |
| *STXBP1* | Not Correlated | 2 groups/Quartiles | p > 0.05 |
| *STXBP5* | Not Correlated | 2 groups/Quartiles | p > 0.05 |
| *SYT7* | Not Correlated | 2 groups/Quartiles | p > 0.05 |
| *SYTL4* | Low Expression | 2 groups | p < 0.01 |
| *TBC1D1* | Not Correlated | 2 groups/Quartiles | p > 0.05 |
| *TBC1D10A* | Not Correlated | 2 groups/Quartiles | p > 0.05 |
| *TBC1D10B* | Not Correlated | 2 groups/Quartiles | p > 0.05 |
| *TBC1D10C* | Not Correlated | 2 groups/Quartiles | p > 0.05 |
| *TBC1D13* | Not Correlated | 2 groups/Quartiles | p > 0.05 |
| *TBC1D15* | Not Correlated | 2 groups/Quartiles | p > 0.05 |
| *TBC1D16* | Not Correlated | 2 groups/Quartiles | p > 0.05 |
| *TBC1D17* | Not Correlated | 2 groups/Quartiles | p > 0.05 |
| *TBC1D18/RABGAP1L* | Not Correlated | 2 groups/Quartiles | p > 0.05 |
| *TBC1D2* | Not Correlated | 2 groups/Quartiles | p > 0.05 |
| *TBC1D20* | Not Correlated | 2 groups/Quartiles | p > 0.05 |
| *TBC1D23* | Not Correlated | 2 groups/Quartiles | p > 0.05 |
| *TBC1D24* | High Expression | 2 groups | p < 0.05 |
| *TBC1D25* | Not Correlated | 2 groups/Quartiles | p > 0.05 |
| *TBC1D3* | Low Expression | Quartiles | p < 0.01 |
| *TBC1D3B* | Not Correlated | 2 groups/Quartiles | p > 0.05 |
| *TBC1D3C* | Not Correlated | 2 groups/Quartiles | p > 0.05 |
| *TBC1D4* | Not Correlated | 2 groups/Quartiles | p > 0.05 |
| *TBC1D5* | Not Correlated | 2 groups/Quartiles | p > 0.05 |
| *TBC1D6/GRTP1* | Not Correlated | 2 groups/Quartiles | p > 0.05 |
| *TBC1D7* | High Expression | 2 groups | p < 0.01 |
| *TGFBRAP1* | Not Correlated | 2 groups/Quartiles | p > 0.05 |
| *TSG101* | Not Correlated | 2 groups/Quartiles | p > 0.05 |
| *USP6* | Not Correlated | 2 groups/Quartiles | p > 0.05 |

**Supplementary table 3:** Exosome-related genes individually associated with the disease-free interval of breast cancer patients (TCGA BRCA cohort, n = 1,037).

| Gene | Reduced DFI | Analyzed | p-value |
| --- | --- | --- | --- |
| *USP6NL* | Not Correlated | 2 groups/Quartiles | p > 0.05 |
| *UVRAG* | Not Correlated | 2 groups/Quartiles | p > 0.05 |
| *VAMP2* | Low Expression | Quartiles | p < 0.05 |
| *VAMP3* | Not Correlated | 2 groups/Quartiles | p > 0.05 |
| *VPS26A* | Not Correlated | 2 groups/Quartiles | p > 0.05 |
| *VPS26B* | Not Correlated | 2 groups/Quartiles | p > 0.05 |
| *VPS29* | Not Correlated | 2 groups/Quartiles | p > 0.05 |
| *VPS35* | Not Correlated | 2 groups/Quartiles | p > 0.05 |
| *VPS8* | Not Correlated | 2 groups/Quartiles | p > 0.05 |
| *VTA1* | High Expression | Quartiles | p < 0.05 |
| *ZFYVE20/RBSN* | High Expression | Quartiles | p < 0.05 |

**Supplementary table 4:** Exosome-related genes individually associated with the progression-free survival of breast cancer patients (TCGA BRCA cohort, n = 1,014).

| Gene | Reduced PFI | Analyzed | p-value |
| --- | --- | --- | --- |
| *ACAP2* | Not Correlated | 2 groups/Quartiles | p > 0.05 |
| *ALS2* | Not Correlated | 2 groups/Quartiles | p > 0.05 |
| *ANKFY1* | Not Correlated | 2 groups/Quartiles | p > 0.05 |
| *APPL1* | Not Correlated | 2 groups/Quartiles | p > 0.05 |
| *BECN1* | High Expression | Quartiles | p < 0.05 |
| *CCDC53* | Not Correlated | 2 groups/Quartiles | p > 0.05 |
| *CHMP1B* | Not Correlated | 2 groups/Quartiles | p > 0.05 |
| *CHMP2B* | Not Correlated | 2 groups/Quartiles | p > 0.05 |
| *CHMP3* | Not Correlated | 2 groups/Quartiles | p > 0.05 |
| *DENND1A* | Not Correlated | 2 groups/Quartiles | p > 0.05 |
| *DENND1B* | Not Correlated | 2 groups/Quartiles | p > 0.05 |
| *DENND1C* | Low Expression | Quartiles | p < 0.01 |
| *DENND2/ST5* | High Expression | Quartiles | p < 0.05 |
| *DENND2A* | Not Correlated | 2 groups/Quartiles | p > 0.05 |
| *DENND2C* | Not Correlated | 2 groups/Quartiles | p > 0.05 |
| *DENND2D* | Not Correlated | 2 groups/Quartiles | p > 0.05 |
| *DENND3* | Not Correlated | 2 groups/Quartiles | p > 0.05 |
| *DENND4A* | Not Correlated | 2 groups/Quartiles | p > 0.05 |
| *DENND4B* | Not Correlated | 2 groups/Quartiles | p > 0.05 |
| *DENND4C* | Not Correlated | 2 groups/Quartiles | p > 0.05 |
| *DNAJC13* | High Expression | Quartiles | p < 0.05 |
| *EEA1* | Not Correlated | 2 groups/Quartiles | p > 0.05 |
| *EVI5* | Not Correlated | 2 groups/Quartiles | p > 0.05 |
| *EVI5L* | Not Correlated | 2 groups/Quartiles | p > 0.05 |
| *FAM21A* | Not Correlated | 2 groups/Quartiles | p > 0.05 |
| *FAM21C* | Not Correlated | 2 groups/Quartiles | p > 0.05 |
| *FAM45A* | Low Expression | 2 groups | p < 0.05 |
| *FIG4* | High Expression | 2 groups | p < 0.05 |
| *FLCN* | Not Correlated | 2 groups/Quartiles | p > 0.05 |
| *GAPVD1* | Not Correlated | 2 groups/Quartiles | p > 0.05 |
| *HSP90AA1* | Not Correlated | 2 groups/Quartiles | p > 0.05 |
| *IST1* | Not Correlated | 2 groups/Quartiles | p > 0.05 |
| *KIAA0196/WASHC5* | Not Correlated | 2 groups/Quartiles | p > 0.05 |
| *KIAA1033/WASHC4* | Not Correlated | 2 groups/Quartiles | p > 0.05 |
| *LEPROT* | Not Correlated | 2 groups/Quartiles | p > 0.05 |
| *LYST* | Not Correlated | 2 groups/Quartiles | p > 0.05 |
| *MON1A* | Not Correlated | 2 groups/Quartiles | p > 0.05 |
| *MON1B* | High Expression | Quartiles | p < 0.05 |
| *MYO5A* | Not Correlated | 2 groups/Quartiles | p > 0.05 |
| *MYO5B* | Not Correlated | 2 groups/Quartiles | p > 0.05 |
| *OCRL* | Not Correlated | 2 groups/Quartiles | p > 0.05 |
| *PARK2* | Not Correlated | 2 groups/Quartiles | p > 0.05 |

**Supplementary table 4:** Exosome-related genes individually associated with the progression-free survival of breast cancer patients (TCGA BRCA cohort, n = 1,014).

| Gene | Reduced PFI | Analyzed | p-value |
| --- | --- | --- | --- |
| *PDCD6IP* | Not Correlated | 2 groups/Quartiles | p > 0.05 |
| *PIK3C3* | Not Correlated | 2 groups/Quartiles | p > 0.05 |
| *PIK3CB* | Not Correlated | 2 groups/Quartiles | p > 0.05 |
| *PIK3R4* | Not Correlated | 2 groups/Quartiles | p > 0.05 |
| *PIKFYVE* | Not Correlated | 2 groups/Quartiles | p > 0.05 |
| *PLEKHM1* | Not Correlated | 2 groups/Quartiles | p > 0.05 |
| *RAB10* | Not Correlated | 2 groups/Quartiles | p > 0.05 |
| *RAB11A* | Not Correlated | 2 groups/Quartiles | p > 0.05 |
| *RAB11B* | Not Correlated | 2 groups/Quartiles | p > 0.05 |
| *RAB12* | Not Correlated | 2 groups/Quartiles | p > 0.05 |
| *RAB14* | Not Correlated | 2 groups/Quartiles | p > 0.05 |
| *RAB21* | Not Correlated | 2 groups/Quartiles | p > 0.05 |
| *RAB27A* | High Expression | 2 groups | p < 0.001 |
| *RAB27B* | Not Correlated | 2 groups/Quartiles | p > 0.05 |
| *RAB28* | High Expression | 2 groups | p < 0.05 |
| *RAB2A* | Not Correlated | 2 groups/Quartiles | p > 0.05 |
| *RAB2B* | Not Correlated | 2 groups/Quartiles | p > 0.05 |
| *RAB33A* | Not Correlated | 2 groups/Quartiles | p > 0.05 |
| *RAB33B* | Not Correlated | 2 groups/Quartiles | p > 0.05 |
| *RAB35* | Not Correlated | 2 groups/Quartiles | p > 0.05 |
| *RAB5A* | Not Correlated | 2 groups/Quartiles | p > 0.05 |
| *RAB5B* | Not Correlated | 2 groups/Quartiles | p > 0.05 |
| *RAB5C* | Not Correlated | 2 groups/Quartiles | p > 0.05 |
| *RAB6A* | Not Correlated | 2 groups/Quartiles | p > 0.05 |
| *RAB6B* | Not Correlated | 2 groups/Quartiles | p > 0.05 |
| *RAB6C* | High Expression | 2 groups | p < 0.05 |
| *RAB7A* | Not Correlated | 2 groups/Quartiles | p > 0.05 |
| *RAB9A* | Not Correlated | 2 groups/Quartiles | p > 0.05 |
| *RAB9B* | Not Correlated | 2 groups/Quartiles | p > 0.05 |
| *RABEP1* | Not Correlated | 2 groups/Quartiles | p > 0.05 |
| *RABGEF1* | Not Correlated | 2 groups/Quartiles | p > 0.05 |
| *RALA* | Not Correlated | 2 groups/Quartiles | p > 0.05 |
| *RALB* | Not Correlated | 2 groups/Quartiles | p > 0.05 |
| *RILP* | Not Correlated | 2 groups/Quartiles | p > 0.05 |
| *RIN1* | Not Correlated | 2 groups/Quartiles | p > 0.05 |
| *RIN2* | Not Correlated | 2 groups/Quartiles | p > 0.05 |
| *RUBCN/KIAA0226* | Not Correlated | 2 groups/Quartiles | p > 0.05 |
| *RUSC1* | Not Correlated | 2 groups/Quartiles | p > 0.05 |
| *RUSC2* | Not Correlated | 2 groups/Quartiles | p > 0.05 |
| *SBF1* | Not Correlated | 2 groups/Quartiles | p > 0.05 |
| *SBF2* | Not Correlated | 2 groups/Quartiles | p > 0.05 |
| *SDCBP* | Not Correlated | 2 groups/Quartiles | p > 0.05 |

**Supplementary table 4:** Exosome-related genes individually associated with the progression-free survival of breast cancer patients (TCGA BRCA cohort, n = 1,014).

| Gene | Reduced PFI | Analyzed | p-value |
| --- | --- | --- | --- |
| *SGSM3* | Not Correlated | 2 groups/Quartiles | p > 0.05 |
| *SLAC2A/MLPH* | High Expression | 2 groups | p < 0.05 |
| *SLAC2B/EXPH5* | High Expression | Quartiles | p < 0.05 |
| *SNX1* | Not Correlated | 2 groups/Quartiles | p > 0.05 |
| *SNX2* | Not Correlated | 2 groups/Quartiles | p > 0.05 |
| *SNX3* | High Expression | 2 groups | p < 0.01 |
| *SNX5* | Not Correlated | 2 groups/Quartiles | p > 0.05 |
| *SNX6* | Not Correlated | 2 groups/Quartiles | p > 0.05 |
| *STAM* | Not Correlated | 2 groups/Quartiles | p > 0.05 |
| *STIP1* | Not Correlated | 2 groups/Quartiles | p > 0.05 |
| *STX12* | Not Correlated | 2 groups/Quartiles | p > 0.05 |
| *STX1A* | Not Correlated | 2 groups/Quartiles | p > 0.05 |
| *STX2* | High Expression | Quartiles | p < 0.05 |
| *STX7* | Not Correlated | 2 groups/Quartiles | p > 0.05 |
| *STXBP1* | High Expression | Quartiles | p < 0.01 |
| *STXBP5* | High Expression | Quartiles | p < 0.05 |
| *SYT7* | Not Correlated | 2 groups/Quartiles | p > 0.05 |
| *SYTL4* | Low Expression | 2 groups | p < 0.001 |
| *TBC1D1* | Not Correlated | 2 groups/Quartiles | p > 0.05 |
| *TBC1D10A* | Not Correlated | 2 groups/Quartiles | p > 0.05 |
| *TBC1D10B* | Not Correlated | 2 groups/Quartiles | p > 0.05 |
| *TBC1D10C* | Low Expression | 2 groups | p < 0.01 |
| *TBC1D13* | Not Correlated | 2 groups/Quartiles | p > 0.05 |
| *TBC1D15* | Not Correlated | 2 groups/Quartiles | p > 0.05 |
| *TBC1D16* | Not Correlated | 2 groups/Quartiles | p > 0.05 |
| *TBC1D17* | Not Correlated | 2 groups/Quartiles | p > 0.05 |
| *TBC1D18/RABGAP1L* | Not Correlated | 2 groups/Quartiles | p > 0.05 |
| *TBC1D2* | Not Correlated | 2 groups/Quartiles | p > 0.05 |
| *TBC1D20* | Not Correlated | 2 groups/Quartiles | p > 0.05 |
| *TBC1D23* | Not Correlated | 2 groups/Quartiles | p > 0.05 |
| *TBC1D24* | High Expression | 2 groups | p < 0.01 |
| *TBC1D25* | Not Correlated | 2 groups/Quartiles | p > 0.05 |
| *TBC1D3* | High Expression | Quartiles | p < 0.05 |
| *TBC1D3B* | Not Correlated | 2 groups/Quartiles | p > 0.05 |
| *TBC1D3C* | Not Correlated | 2 groups/Quartiles | p > 0.05 |
| *TBC1D4* | Not Correlated | 2 groups/Quartiles | p > 0.05 |
| *TBC1D5* | Not Correlated | 2 groups/Quartiles | p > 0.05 |
| *TBC1D6/GRTP1* | Not Correlated | 2 groups/Quartiles | p > 0.05 |
| *TBC1D7* | Not Correlated | 2 groups/Quartiles | p > 0.05 |
| *TGFBRAP1* | Not Correlated | 2 groups/Quartiles | p > 0.05 |
| *TSG101* | Not Correlated | 2 groups/Quartiles | p > 0.05 |
| *USP6* | Not Correlated | 2 groups/Quartiles | p > 0.05 |

**Supplementary table 4:** Exosome-related genes individually associated with the progression-free survival of breast cancer patients (TCGA BRCA cohort, n = 1,014).

| Gene | Reduced PFI | Analyzed | p-value |
| --- | --- | --- | --- |
| *USP6NL* | Not Correlated | 2 groups/Quartiles | p > 0.05 |
| *UVRAG* | Not Correlated | 2 groups/Quartiles | p > 0.05 |
| *VAMP2* | Not Correlated | 2 groups/Quartiles | p > 0.05 |
| *VAMP3* | Not Correlated | 2 groups/Quartiles | p > 0.05 |
| *VPS26A* | Not Correlated | 2 groups/Quartiles | p > 0.05 |
| *VPS26B* | Not Correlated | 2 groups/Quartiles | p > 0.05 |
| *VPS29* | Not Correlated | 2 groups/Quartiles | p > 0.05 |
| *VPS35* | Not Correlated | 2 groups/Quartiles | p > 0.05 |
| *VPS8* | Not Correlated | 2 groups/Quartiles | p > 0.05 |
| *VTA1* | High Expression | Quartiles | p < 0.05 |
| *ZFYVE20/RBSN* | High Expression | Quartiles | p < 0.05 |

**Supplementary table 5:** Exosome-related gene mutations in breast cancers. Genes mutated in more than 1% of the TCGA-BRCA samples analyzed are represented (TCGA cohort; n = 965).

| Gene | Cases | Mutation  Rate | Sub | Del | Ins | Syn | Mis | SG | FS | Inf Del | SR | PA | VEP  (Hi) | SIFT  (Deleterious) | PolyPhen  (PO or PR) |
| --- | --- | --- | --- | --- | --- | --- | --- | --- | --- | --- | --- | --- | --- | --- | --- |
| *DNAJC13* | 20 | 0.021 | 9 | 0 | 1 | 3 | 4 | 1 | 0 | 0 | 1 | 1 | 2 | 2 | 2 |
| *LYST* | 20 | 0.021 | 10 | 0 | 0 | 1 | 7 | 2 | 0 | 0 | 0 | 0 | 2 | 5 | 3 |
| *TBC1D4* | 19 | 0.020 | 9 | 1 | 0 | 1 | 7 | 1 | 1 | 0 | 0 | 0 | 2 | 3 | 3 |
| *MYO5B* | 18 | 0.019 | 11 | 0 | 0 | 2 | 8 | 0 | 0 | 0 | 0 | 0 | 0 | 5 | 3 |
| *DENND4B* | 15 | 0.016 | 11 | 0 | 0 | 1 | 9 | 0 | 0 | 0 | 0 | 0 | 0 | 6 | 4 |
| *PIKFYVE* | 15 | 0.016 | 9 | 1 | 0 | 3 | 5 | 0 | 1 | 0 | 1 | 0 | 1 | 3 | 3 |
| *EEA1* | 14 | 0.015 | 9 | 1 | 0 | 2 | 7 | 0 | 0 | 1 | 0 | 0 | 0 | 5 | 6 |
| *SBF2* | 13 | 0.013 | 10 | 0 | 0 | 2 | 7 | 1 | 0 | 0 | 0 | 0 | 1 | 6 | 6 |
| *MYO5A* | 13 | 0.013 | 10 | 0 | 0 | 3 | 5 | 2 | 0 | 0 | 0 | 0 | 2 | 2 | 2 |
| *DENND1A* | 12 | 0.012 | 9 | 1 | 0 | 3 | 5 | 0 | 0 | 1 | 1 | 0 | 1 | 3 | 4 |
| *EXPH5* | 12 | 0.012 | 10 | 0 | 0 | 3 | 7 | 0 | 0 | 0 | 0 | 0 | 0 | 2 | 2 |
| *STXBP5* | 12 | 0.012 | 12 | 0 | 0 | 6 | 4 | 0 | 0 | 0 | 0 | 0 | 0 | 3 | 3 |
| *PIK3CB* | 11 | 0.011 | 10 | 0 | 0 | 1 | 8 | 1 | 0 | 0 | 0 | 0 | 1 | 4 | 3 |
| *MLPH* | 11 | 0.011 | 10 | 0 | 0 | 5 | 5 | 0 | 0 | 0 | 0 | 0 | 0 | 3 | 2 |
| *TBC1D25* | 11 | 0.011 | 10 | 0 | 0 | 0 | 10 | 0 | 0 | 0 | 0 | 0 | 0 | 4 | 3 |

Sub: substitution, Del: deletion, Ins: insertion, Syn: synonymous, Mis: missense, SG: stop gain, FS: frameshift, Inf Del: inframe deletion, SR: splicing region, PA: protein altering, Hi: high, PO: possibly damaging, PR: probably damaging.

**Supplementary table 6:** Exosome-related genes individually associated with the overall survival of breast cancer patients (NKI-295 cohort, n = 295).

| Gene | Reduced OS | Analyzed | p-value |
| --- | --- | --- | --- |
| *ACAP2* | N/A | N/A | N/A |
| *ALS2* | Not Correlated | 2 groups/Quartiles | p > 0.05 |
| *ANKFY1* | N/A | N/A | N/A |
| *APPL1* | Low Expression | 2 groups | p < 0.01 |
| *BECN1* | N/A | N/A | N/A |
| *CCDC53* | Not Correlated | 2 groups/Quartiles | p > 0.05 |
| *CHMP1B* | Not Correlated | 2 groups/Quartiles | p > 0.05 |
| *CHMP2B* | N/A | N/A | N/A |
| *CHMP3* | N/A | N/A | N/A |
| *DENND1A* | High Expression | Quartiles | p < 0.01 |
| *DENND1B* | N/A | N/A | N/A |
| *DENND1C* | Not Correlated | 2 groups/Quartiles | p > 0.05 |
| *DENND2/ST5* | Not Correlated | 2 groups/Quartiles | p > 0.05 |
| *DENND2A* | N/A | N/A | N/A |
| *DENND2C* | N/A | N/A | N/A |
| *DENND2D* | N/A | N/A | N/A |
| *DENND3* | High Expression | Quartiles | p < 0.01 |
| *DENND4A* | N/A | N/A | N/A |
| *DENND4B* | Not Correlated | 2 groups/Quartiles | p > 0.05 |
| *DENND4C* | Low Expression | 2 groups | p < 0.001 |
| *DNAJC13* | Not Correlated | 2 groups/Quartiles | p > 0.05 |
| *EEA1* | Low Expression | 2 groups | p < 0.05 |
| *EVI5* | N/A | N/A | N/A |
| *EVI5L* | Not Correlated | 2 groups/Quartiles | p > 0.05 |
| *FAM21A* | Not Correlated | 2 groups/Quartiles | p > 0.05 |
| *FAM21C* | N/A | N/A | N/A |
| *FAM45A* | Low Expression | Quartiles | p < 0.05 |
| *FIG4* | Low Expression | 2 groups | p < 0.05 |
| *FLCN* | N/A | N/A | N/A |
| *GAPVD1* | Not Correlated | 2 groups/Quartiles | p > 0.05 |
| *HSP90AA1* | N/A | N/A | N/A |
| *IST1* | N/A | N/A | N/A |
| *KIAA0196/WASHC5* | Not Correlated | 2 groups/Quartiles | p > 0.05 |
| *KIAA1033/WASHC4* | Not Correlated | 2 groups/Quartiles | p > 0.05 |
| *LEPROT* | Low Expression | Quartiles | p < 0.05 |
| *LYST* | Not Correlated | 2 groups/Quartiles | p < 0.05 |
| *MON1A* | N/A | N/A | N/A |
| *MON1B* | N/A | N/A | N/A |
| *MYO5A* | Not Correlated | 2 groups/Quartiles | p > 0.05 |
| *MYO5B* | Low Expression | 2 groups | p < 0.001 |
| *OCRL* | High Expression | 2 groups | p < 0.05 |
| *PARK2* | N/A | N/A | N/A |

**Supplementary table 6:** Exosome-related genes individually associated with the overall survival of breast cancer patients (NKI-295 cohort, n = 295).

| Gene | Reduced OS | Analyzed | p-value |
| --- | --- | --- | --- |
| *PDCD6IP* | N/A | N/A | N/A |
| *PIK3C3* | N/A | N/A | N/A |
| *PIK3CB* | N/A | N/A | N/A |
| *PIK3R4* | Not Correlated | 2 groups/Quartiles | p > 0.05 |
| *PIKFYVE* | N/A | N/A | N/A |
| *PLEKHM1* | Low Expression | 2 groups | p < 0.05 |
| *RAB10* | N/A | N/A | N/A |
| *RAB11A* | Not Correlated | 2 groups/Quartiles | p > 0.05 |
| *RAB11B* | N/A | N/A | N/A |
| *RAB12* | N/A | N/A | N/A |
| *RAB14* | Not Correlated | 2 groups/Quartiles | p > 0.05 |
| *RAB21* | Not Correlated | 2 groups/Quartiles | p > 0.05 |
| *RAB27A* | N/A | N/A | N/A |
| *RAB27B* | Low Expression | 2 groups | p < 0.001 |
| *RAB28* | N/A | N/A | N/A |
| *RAB2A* | N/A | N/A | N/A |
| *RAB2B* | Not Correlated | 2 groups/Quartiles | p > 0.05 |
| *RAB33A* | Not Correlated | 2 groups/Quartiles | p > 0.05 |
| *RAB33B* | N/A | N/A | N/A |
| *RAB35* | High Expression | 2 groups | p < 0.05 |
| *RAB5A* | N/A | N/A | N/A |
| *RAB5B* | N/A | N/A | N/A |
| *RAB5C* | N/A | N/A | N/A |
| *RAB6A* | Not Correlated | 2 groups/Quartiles | p > 0.05 |
| *RAB6B* | Not Correlated | 2 groups/Quartiles | p > 0.05 |
| *RAB6C* | N/A | N/A | N/A |
| *RAB7A* | N/A | N/A | N/A |
| *RAB9A* | High Expression | Quartiles | p < 0.05 |
| *RAB9B* | Not Correlated | 2 groups/Quartiles | p > 0.05 |
| *RABEP1* | N/A | N/A | N/A |
| *RABGEF1* | N/A | N/A | N/A |
| *RALA* | N/A | N/A | N/A |
| *RALB* | N/A | N/A | N/A |
| *RILP* | N/A | N/A | N/A |
| *RIN1* | Not Correlated | 2 groups/Quartiles | p > 0.05 |
| *RIN2* | Not Correlated | 2 groups/Quartiles | p > 0.05 |
| *RUBCN/KIAA0226* | High Expression | Quartiles | p < 0.05 |
| *RUSC1* | High Expression | 2 groups | p < 0.05 |
| *RUSC2* | Not Correlated | 2 groups/Quartiles | p > 0.05 |
| *SBF1* | N/A | N/A | N/A |
| *SBF2* | N/A | N/A | N/A |
| *SDCBP* | Not Correlated | 2 groups/Quartiles | p > 0.05 |

**Supplementary table 6:** Exosome-related genes individually associated with the overall survival of breast cancer patients (NKI-295 cohort, n = 295).

| Gene | Reduced OS | Analyzed | p-value |
| --- | --- | --- | --- |
| *SGSM3* | High Expression | Quartiles | p < 0.05 |
| *SLAC2A/MLPH* | N/A | N/A | N/A |
| *SLAC2B/EXPH5* | Not Correlated | 2 groups/Quartiles | p > 0.05 |
| *SNX1* | Low Expression | 2 groups | p < 0.001 |
| *SNX2* | N/A | N/A | N/A |
| *SNX3* | Not Correlated | 2 groups/Quartile | p > 0.05 |
| *SNX5* | Low Expression | 2 groups | p < 0.01 |
| *SNX6* | N/A | N/A | N/A |
| *STAM* | N/A | N/A | N/A |
| *STIP1* | High Expression | 2 groups | p < 0.001 |
| *STX12* | Low Expression | 2 groups | p < 0.05 |
| *STX1A* | High Expression | 2 groups | p < 0.05 |
| *STX2* | High Expression | Quartiles | p < 0.05 |
| *STX7* | N/A | N/A | N/A |
| *STXBP1* | Not Correlated | 2 groups/Quartile | p > 0.05 |
| *STXBP5* | Not Correlated | 2 groups/Quartiles | p > 0.05 |
| *SYT7* | Not Correlated | 2 groups/Quartiles | p > 0.05 |
| *SYTL4* | Low Expression | 2 groups | p < 0.05 |
| *TBC1D1* | Not Correlated | 2 groups/Quartiles | p < 0.05 |
| *TBC1D10A* | N/A | N/A | N/A |
| *TBC1D10B* | Not Correlated | 2 groups/Quartiles | p > 0.05 |
| *TBC1D10C* | N/A | N/A | N/A |
| *TBC1D13* | Low Expression | 2 groups | p < 0.05 |
| *TBC1D15* | Low Expression | 2 groups | p < 0.05 |
| *TBC1D16* | N/A | N/A | N/A |
| *TBC1D17* | N/A | N/A | N/A |
| *TBC1D18/RABGAP1L* | N/A | N/A | N/A |
| *TBC1D2* | Not Correlated | 2 groups/Quartiles | p > 0.05 |
| *TBC1D20* | Not Correlated | 2 groups/Quartiles | p > 0.05 |
| *TBC1D23* | N/A | N/A | N/A |
| *TBC1D24* | Not Correlated | 2 groups/Quartiles | p > 0.05 |
| *TBC1D25* | Not Correlated | 2 groups/Quartiles | p > 0.05 |
| *TBC1D3* | Not Correlated | 2 groups/Quartiles | p > 0.05 |
| *TBC1D3B* | N/A | N/A | N/A |
| *TBC1D3C* | N/A | N/A | N/A |
| *TBC1D4* | Low Expression | 2 groups | p < 0.05 |
| *TBC1D5* | Not Correlated | 2 groups/Quartiles | p > 0.05 |
| *TBC1D6/GRTP1* | Not Correlated | 2 groups/Quartiles | p > 0.05 |
| *TBC1D7* | N/A | N/A | N/A |
| *TGFBRAP1* | Not Correlated | 2 groups/Quartiles | p > 0.05 |
| *TSG101* | Not Correlated | 2 groups/Quartiles | p > 0.05 |
| *USP6* | Not Correlated | 2 groups/Quartiles | p > 0.05 |

**Supplementary table 6:** Exosome-related genes individually associated with the overall survival of breast cancer patients (NKI-295 cohort, n = 295).

| Gene | Reduced OS | Analyzed | p-value |
| --- | --- | --- | --- |
| *USP6NL* | High Expression | 2 groups | p < 0.05 |
| *UVRAG* | N/A | N/A | N/A |
| *VAMP2* | Low Expression | 2 groups | p < 0.05 |
| *VAMP3* | N/A | N/A | N/A |
| *VPS26A* | Not Correlated | 2 groups/Quartiles | p > 0.05 |
| *VPS26B* | Not Correlated | 2 groups/Quartiles | p > 0.05 |
| *VPS29* | Not Correlated | 2 groups/Quartiles | p > 0.05 |
| *VPS35* | High Expression | 2 groups | p < 0.01 |
| *VPS8* | Not Correlated | 2 groups/Quartiles | p > 0.05 |
| *VTA1* | N/A | N/A | N/A |
| *ZFYVE20/RBSN* | Low Expression | Quartiles | p < 0.05 |

**Supplementary table 7:** Exosome-related genes individually associated with the distant metastasis-free survival of breast cancer patients (NKI-295 cohort, n = 101).

| Gene | Reduced DMFS | Analyzed | p-value | |  |
| --- | --- | --- | --- | --- | --- |
| *ACAP2* | N/A | N/A | N/A | |  |
| *ALS2* | Not Correlated | 2 groups/Quartiles | p > 0.05 | |  |
| *ANKFY1* | N/A | N/A | N/A | |  |
| *APPL1* | Not Correlated | 2 groups/Quartiles | p > 0.05 | |  |
| *BECN1* | N/A | N/A | N/A | |  |
| *CCDC53* | Not Correlated | 2 groups/Quartiles | p > 0.05 | |  |
| *CHMP1B* | Not Correlated | 2 groups/Quartiles | p > 0.05 | |  |
| *CHMP2B* | N/A | N/A | N/A | |  |
| *CHMP3* | N/A | N/A | N/A | |  |
| *DENND1A* | Not Correlated | 2 groups/Quartiles | p > 0.05 | |  |
| *DENND1B* | N/A | N/A | N/A | |  |
| *DENND1C* | Not Correlated | 2 groups/Quartiles | p > 0.05 | |  |
| *DENND2/ST5* | Not Correlated | 2 groups/Quartiles | p > 0.05 | |  |
| *DENND2A* | N/A | N/A | N/A | |  |
| *DENND2C* | N/A | N/A | N/A | |  |
| *DENND2D* | N/A | N/A | N/A | |  |
| *DENND3* | Not Correlated | 2 groups/Quartiles | p > 0.05 | |  |
| *DENND4A* | N/A | N/A | N/A | |  |
| *DENND4B* | Not Correlated | 2 groups/Quartiles | p > 0.05 | |  |
| *DENND4C* | Not Correlated | 2 groups/Quartiles | p > 0.05 | |  |
| *DNAJC13* | Low Expression | 2 groups | p < 0.05 | |  |
| *EEA1* | Low Expression | 2 groups | p < 0.05 | |  |
| *EVI5* | N/A | N/A | N/A | |  |
| *EVI5L* | Not Correlated | 2 groups/Quartiles | p > 0.05 | |  |
| *FAM21A* | Not Correlated | 2 groups/Quartiles | p > 0.05 | |  |
| *FAM21C* | N/A | N/A | N/A | |  |
| *FAM45A* | Not Correlated | 2 groups/Quartiles | p > 0.05 | |  |
| *FIG4* | Not Correlated | 2 groups/Quartiles | p > 0.05 | |  |
| *FLCN* | N/A | N/A | N/A | |  |
| *GAPVD1* | Not Correlated | 2 groups/Quartiles | Not Correlated | |  |
| *HSP90AA1* | N/A | N/A | N/A | |  |
| *IST1* | N/A | N/A | N/A | |  |
| *KIAA0196/WASHC5* | Not Correlated | 2 groups/Quartiles | p > 0.05 | |  |
| *KIAA1033/WASHC4* | Not Correlated | 2 groups/Quartiles | p > 0.05 | |  |
| *LEPROT* | Not Correlated | 2 groups/Quartiles | p > 0.05 | |  |
| *LYST* | Not Correlated | 2 groups/Quartiles | p > 0.05 | |  |
| *MON1A* | N/A | N/A | N/A | |  |
| *MON1B* | N/A | N/A | N/A | |  |
| *MYO5A* | Not Correlated | 2 groups/Quartiles | | p > 0.05 | |
| *MYO5B* | Not Correlated | 2 groups/Quartiles | | p > 0.05 | |
| *OCRL* | Not Correlated | 2 groups/Quartiles | | p > 0.05 | |
| *PARK2* | N/A | N/A | N/A | |  |

**Supplementary table 7:** Exosome-related genes individually associated with the distant metastasis-free survival of breast cancer patients (NKI-295 cohort, n = 101).

| Gene | Reduced DMFS | Analyzed | p-value |
| --- | --- | --- | --- |
| *PDCD6IP* | N/A | N/A | N/A |
| *PIK3C3* | N/A | N/A | N/A |
| *PIK3CB* | N/A | N/A | N/A |
| *PIK3R4* | Not Correlated | 2 groups/Quartiles | p > 0.05 |
| *PIKFYVE* | N/A | N/A | N/A |
| *PLEKHM1* | Not Correlated | 2 groups/Quartiles | p > 0.05 |
| *RAB10* | N/A | N/A | N/A |
| *RAB11A* | Not Correlated | 2 groups/Quartiles | p > 0.05 |
| *RAB11B* | N/A | N/A | N/A |
| *RAB12* | N/A | N/A | N/A |
| *RAB14* | Not Correlated | 2 groups/Quartiles | p > 0.05 |
| *RAB21* | Not Correlated | 2 groups/Quartiles | p > 0.05 |
| *RAB27A* | N/A | N/A | N/A |
| *RAB27B* | Not Correlated | 2 groups/Quartiles | p > 0.05 |
| *RAB28* | N/A | N/A | N/A |
| *RAB2A* | N/A | N/A | N/A |
| *RAB2B* | Not Correlated | 2 groups/Quartiles | p > 0.05 |
| *RAB33A* | High Expression | 2 groups | p < 0.05 |
| *RAB33B* | N/A | N/A | N/A |
| *RAB35* | Not Correlated | 2 groups/Quartiles | p > 0.05 |
| *RAB5A* | N/A | N/A | N/A |
| *RAB5B* | N/A | N/A | N/A |
| *RAB5C* | N/A | N/A | N/A |
| *RAB6A* | Not Correlated | 2 groups/Quartiles | p > 0.05 |
| *RAB6B* | Not Correlated | 2 groups/Quartiles | p > 0.05 |
| *RAB6C* | N/A | N/A | N/A |
| *RAB7A* | N/A | N/A | N/A |
| *RAB9A* | Not Correlated | 2 groups/Quartiles | p > 0.05 |
| *RAB9B* | Not Correlated | 2 groups/Quartiles | p > 0.05 |
| *RABEP1* | N/A | N/A | N/A |
| *RABGEF1* | N/A | N/A | N/A |
| *RALA* | N/A | N/A | N/A |
| *RALB* | N/A | N/A | N/A |
| *RILP* | N/A | N/A | N/A |
| *RIN1* | Not Correlated | 2 groups/Quartiles | p > 0.05 |
| *RIN2* | Not Correlated | 2 groups/Quartiles | p > 0.05 |
| *RUBCN/KIAA0226* | Not Correlated | 2 groups/Quartiles | p > 0.05 |
| *RUSC1* | Not Correlated | 2 groups/Quartiles | p > 0.05 |
| *RUSC2* | Not Correlated | 2 groups/Quartiles | p > 0.05 |
| *SBF1* | N/A | N/A | N/A |
| *SBF2* | N/A | N/A | N/A |
| *SDCBP* | Not Correlated | 2 groups/Quartiles | p > 0.05 |

**Supplementary table 7:** Exosome-related genes individually associated with the distant metastasis-free survival of breast cancer patients (NKI-295 cohort, n = 101).

| Gene | Reduced DMFS | Analyzed | p-value |
| --- | --- | --- | --- |
| *SGSM3* | Not Correlated | 2 groups/Quartiles | p > 0.05 |
| *SLAC2A/MLPH* | N/A | N/A | N/A |
| *SLAC2B/EXPH5* | Not Correlated | 2 groups/Quartiles | p > 0.05 |
| *SNX1* | Low Expression | 2 groups | p < 0.05 |
| *SNX2* | N/A | N/A | N/A |
| *SNX3* | Not Correlated | 2 groups/Quartiles | p > 0.05 |
| *SNX5* | Low Expression | 2 groups | p < 0.05 |
| *SNX6* | N/A | N/A | N/A |
| *STAM* | N/A | N/A | N/A |
| *STIP1* | High Expression | 2 groups | p < 0.01 |
| *STX12* | Not Correlated | 2 groups/Quartiles | p > 0.05 |
| *STX1A* | Not Correlated | 2 groups/Quartiles | p > 0.05 |
| *STX2* | Not Correlated | 2 groups/Quartiles | p > 0.05 |
| *STX7* | N/A | N/A | N/A |
| *STXBP1* | Not Correlated | 2 groups/Quartiles | p > 0.05 |
| *STXBP5* | Not Correlated | 2 groups/Quartiles | p > 0.05 |
| *SYT7* | Not Correlated | 2 groups/Quartiles | p > 0.05 |
| *SYTL4* | Low Expression | 2 groups | p < 0.05 |
| *TBC1D1* | Not Correlated | 2 groups/Quartiles | p > 0.05 |
| *TBC1D10A* | N/A | N/A | N/A |
| *TBC1D10B* | Not Correlated | 2 groups/Quartiles | p > 0.05 |
| *TBC1D10C* | N/A | N/A | N/A |
| *TBC1D13* | Not Correlated | 2 groups/Quartiles | p > 0.05 |
| *TBC1D15* | Not Correlated | 2 groups/Quartiles | p > 0.05 |
| *TBC1D16* | N/A | N/A | N/A |
| *TBC1D17* | N/A | N/A | N/A |
| *TBC1D18/RABGAP1L* | N/A | N/A | N/A |
| *TBC1D2* | Not Correlated | 2 groups/Quartiles | p > 0.05 |
| *TBC1D20* | Not Correlated | 2 groups/Quartiles | p > 0.05 |
| *TBC1D23* | N/A | N/A | N/A |
| *TBC1D24* | Not Correlated | 2 groups/Quartiles | p > 0.05 |
| *TBC1D25* | High Expression | Quartiles | p < 0.05 |
| *TBC1D3* | Not Correlated | 2 groups/Quartiles | p > 0.05 |
| *TBC1D3B* | N/A | N/A | N/A |
| *TBC1D3C* |  |  |  |
| *TBC1D4* | Not Correlated | 2 groups/Quartiles | p > 0.05 |
| *TBC1D5* | Not Correlated | 2 groups/Quartiles | p > 0.05 |
| *TBC1D6/GRTP1* | Not Correlated | 2 groups/Quartiles | p > 0.05 |
| *TBC1D7* | N/A | N/A | N/A |
| *TGFBRAP1* | Not Correlated | 2 groups/Quartiles | p > 0.05 |
| *TSG101* | Not Correlated | 2 groups/Quartiles | p > 0.05 |
| *USP6* | Not Correlated | 2 groups/Quartiles | p > 0.05 |

**Supplementary table 7:** Exosome-related genes individually associated with the distant metastasis-free survival of breast cancer patients (NKI-295 cohort, n = 101).

| Gene | Reduced DMFS | Analyzed | p-value |
| --- | --- | --- | --- |
| *USP6NL* | Not Correlated | 2 groups/Quartiles | p > 0.05 |
| *UVRAG* | N/A | N/A | N/A |
| *VAMP2* | Not Correlated | 2 groups/Quartiles | p > 0.05 |
| *VAMP3* | N/A | N/A | N/A |
| *VPS26A* | Not Correlated | 2 groups/Quartiles | p > 0.05 |
| *VPS26B* | Not Correlated | 2 groups/Quartiles | p > 0.05 |
| *VPS29* | Not Correlated | 2 groups/Quartiles | p > 0.05 |
| *VPS35* | Not Correlated | 2 groups/Quartiles | p > 0.05 |
| *VPS8* | Not Correlated | 2 groups/Quartiles | p > 0.05 |
| *VTA1* | N/A | N/A | N/A |
| *ZFYVE20/RBSN* | Not Correlated | 2 groups/Quartiles | p > 0.05 |

**Supplementary table 8:** Cox proportional hazards model for the selected variables considering the overall survival of breast cancer patients (TCGA BRCA).

|  |  |  |  | 95% CI | |  |  |
| --- | --- | --- | --- | --- | --- | --- | --- |
| Covariates | Coefficient | Standard Error | HR | Lower | Upper | p-value | |
| Signature IV score | 1.320 | 0.611 | 3.745 | 1.131 | 12.397 | **0.031** | |
| Menopause status | 1.108 | 0.374 | 3.030 | 1.456 | 6.303 | **0.003** | |
| T stage | 0.023 | 0.223 | 1.545 | 0.661 | 1.583 | 0.918 | |
| M stage | 0.695 | 0.650 | 2.004 | 0.560 | 7.168 | 0.285 | |
| N stage | 0.432 | 0.238 | 1.540 | 0.965 | 2.455 | 0.067 | |
| Tumor stage | 0.435 | 0.394 | 1.545 | 0.714 | 3.343 | 0.270 | |
| ER status | -0.301 | 0.492 | 0.740 | 0.282 | 1.942 | 0.541 | |
| PR status | -0.164 | 0.460 | 0.848 | 0.344 | 2.092 | 0.721 | |

CI = Confidence interval. HR = Hazard ratio. Menopause status: Pre-menopause = 0, Post-menopause = 1. ER status: Negative = 0, Positive = 1. PR status: Negative = 0, Positive = 1. Statistically significant differences are highlighted in bold.

**Supplementary table 9:** Cox proportional hazards model for the selected variables considering the overall survival of breast cancer patients (NKI-295).

|  |  |  |  | 95% CI | |  |  |
| --- | --- | --- | --- | --- | --- | --- | --- |
| Covariates | Coefficient | Standard Error | HR | Lower | Upper | p-value | |
| Signature IV score | 0.650 | 0.213 | 1.916 | 1.261 | 2.910 | **0.002** | |
| LN status | 0.032 | 0.127 | 1.012 | 0.862 | 1.293 | 0.850 | |
| ER status | -1.257 | 0.230 | 0.285 | 0.181 | 0.447 | **4.56e-08** | |

CI = Confidence interval. HR = Hazard ratio. LN (lymph node) status: Negative = 0, Positive = 1. ER status: Negative = 0, Positive = 1. Statistically significant differences are highlighted in bold.

**Supplementary table 10:** Cox proportional hazards model for the selected variables considering the distant metastasis-free survival of breast cancer patients (NKI-295).

|  |  |  |  | 95% CI | |  |  |
| --- | --- | --- | --- | --- | --- | --- | --- |
| Covariates | Coefficient | Standard Error | HR | Lower | Upper | p-value | |
| Signature IV score | 0.587 | 0.191 | 1.799 | 1.237 | 2.617 | **0.002** | |
| LN status | 0.074 | 0.222 | 1.124 | 0.654 | 1.432 | 0.981 | |
| ER status | -0.717 | 0.217 | 0.488 | 0.379 | 0.747 | **9.6e-4** | |

CI = Confidence interval. HR = Hazard ratio. LN (lymph node) status: Negative = 0, Positive = 1. ER status: Negative = 0, Positive = 1. Statistically significant differences are highlighted in bold.

**Supplementary table 11:** List of TGF-β responsive genes (TBRS).

| Gene | Ensembl ID | Chromosome |
| --- | --- | --- |
| *ABTB2* | ENSG00000166016.5 | chr11 |
| \| *ACSBG1* \| \| --- \| | ENSG00000103740.8 | chr15 |
| \| *ALOX5AP* \| \| --- \| | ENSG00000103740.8 | chr13 |
| \| *AMIGO2* \| \| --- \| | ENSG00000139211.6 | chr12 |
| \| *ANGPTL4* \| \| --- \| | ENSG00000139211.6 | chr19 |
| \| *TRANK1* \| \| --- \| | ENSG00000168016.1 | chr3 |
| \| *ARFGAP1* \| \| --- \| | ENSG00000101199.11 | chr20 |
| \| *BET1L* \| \| --- \| | ENSG00000177951.16 | chr11 |
| \| *BHMT* \| \| --- \| | ENSG00000145692.13 | chr5 |
| \| *BMPR2* \| \| --- \| | ENSG00000204217.11 | chr2 |
| \| *C18orf25* \| \| --- \| | ENSG00000152242.9 | chr18 |
| \| *C3orf52* \| \| --- \| | ENSG00000114529.11 | chr3 |
| \| *CCDC93* \| \| --- \| | ENSG00000125633.9 | chr2 |
| \| *CD1E* \| \| --- \| | ENSG00000158488.14 | chr1 |
| \| *CDKN1A* \| \| --- \| | ENSG00000124762.12 | chr6 |
| \| *CENPF* \| \| --- \| | ENSG00000117724.11 | chr1 |
| \| *COL1A1* \| \| --- \| | ENSG00000108821.12 | chr17 |
| \| *COL4A1* \| \| --- \| | ENSG00000187498.13 | chr13 |
| \| *COL4A2* \| \| --- \| | ENSG00000134871.16 | chr13 |
| \| *COL8A2* \| \| --- \| | ENSG00000171812.9 | chr1 |
| \| *CTGF* \| \| --- \| | ENSG00000118523.5 | chr6 |
| \| *CUBN* \| \| --- \| | ENSG00000107611.13 | chr10 |
| \| *DNAJC7* \| \| --- \| | ENSG00000168259.13 | chr17 |
| \| *EDN1* \| \| --- \| | ENSG00000078401.6 | chr6 |
| \| *ELK3* \| \| --- \| | ENSG00000111145.6 | chr12 |
| \| *ETS2* \| \| --- \| | ENSG00000157557.10 | chr21 |
| \| *FHL3* \| \| --- \| | ENSG00000183386.8 | chr1 |
| \| *FILIP1L* \| \| --- \| | ENSG00000168386.17 | chr3 |
| \| *FNDC3B* \| \| --- \| | ENSG00000075420.11 | chr3 |
| \| *FSTL3* \| \| --- \| | ENSG00000070404.8 | chr19 |
| \| *FZR1* \| \| --- \| | ENSG00000105325.12 | chr19 |
| \| *GADD45B* \| \| --- \| | ENSG00000099860.7 | chr19 |
| \| *GRB10* \| \| --- \| | ENSG00000106070.16 | chr7 |
| \| *HLX* \| \| --- \| | ENSG00000136630.12 | chr1 |
| \| *HMOX1* \| \| --- \| | ENSG00000100292.15 | chr22 |
| \| *HRH1* \| \| --- \| | ENSG00000196639.6 | chr3 |
| \| *IL11* \| \| --- \| | ENSG00000095752.5 | chr19 |
| \| *JAG1* \| \| --- \| | ENSG00000101384.10 | chr20 |
| \| *JUN* \| \| --- \| | ENSG00000177606.6 | chr1 |
| \| *JUNB* \| \| --- \| | ENSG00000171223.5 | chr19 |

**Supplementary table 11 (continued):** List of TGF-β responsive genes (TBRS).

| Gene | Ensembl ID | Chromosome |
| --- | --- | --- |
| \| *LARP6* \| \| --- \| | ENSG00000166173.10 | chr15 |
| \| *LBH* \| \| --- \| | ENSG00000213626.10 | chr2 |
| \| *LEMD3* \| \| --- \| | ENSG00000174106.2 | chr12 |
| \| *LMCD1* \| \| --- \| | ENSG00000071282.10 | chr3 |
| \| *MAP3K4* \| \| --- \| | ENSG00000085511.18 | chr6 |
| \| *MLXIP* \| \| --- \| | ENSG00000175727.12 | chr12 |
| \| *NCOR2* \| \| --- \| | ENSG00000196498.12 | chr12 |
| \| *NDST1* \| \| --- \| | ENSG00000070614.13 | chr5 |
| \| *NEDD9* \| \| --- \| | ENSG00000111859.15 | chr6 |
| \| *NPAS1* \| \| --- \| | ENSG00000130751.8 | chr19 |
| \| *PASK* \| \| --- \| | ENSG00000115687.12 | chr2 |
| \| *PDGFA* \| \| --- \| | ENSG00000197461.12 | chr7 |
| \| *PDLIM4* \| \| --- \| | ENSG00000131435.11 | chr5 |
| \| *PFKFB3* \| \| --- \| | ENSG00000170525.17 | chr10 |
| \| *PHLDB1* \| \| --- \| | ENSG00000019144.15 | chr11 |
| \| *PKIA* \| \| --- \| | ENSG00000171033.11 | chr8 |
| \| *PLK3* \| \| --- \| | ENSG00000173846.11 | chr1 |
| \| *PPP1R13L* \| \| --- \| | ENSG00000104881.13 | chr19 |
| \| *PTH* \| \| --- \| | ENSG00000152266.5 | chr11 |
| \| *PVRIG* \| \| --- \| | ENSG00000213413.2 | chr7 |
| \| *RARA* \| \| --- \| | ENSG00000131759.16 | chr17 |
| \| *RASL10A* \| \| --- \| | ENSG00000100276.9 | chr22 |
| \| *RBMS1* \| \| --- \| | ENSG00000153250.16 | chr2 |
| \| *RHOB* \| \| --- \| | ENSG00000143878.9 | chr2 |
| \| *SERPINE1* \| \| --- \| | ENSG00000106366.8 | chr7 |
| \| *SKIL* \| \| --- \| | ENSG00000136603.12 | chr3 |
| \| *SLC17A3* \| \| --- \| | ENSG00000124564.16 | chr6 |
| \| *SMAD7* \| \| --- \| | ENSG00000101665.7 | chr18 |
| \| *SMOX* \| \| --- \| | ENSG00000088826.16 | chr20 |
| \| *SMTN* \| \| --- \| | ENSG00000183963.17 | chr22 |
| \| *SMURF1* \| \| --- \| | ENSG00000198742.8 | chr7 |
| \| *SNAI1* \| \| --- \| | ENSG00000124216.3 | chr20 |
| \| *SPHK1* \| \| --- \| | ENSG00000176170.12 | chr17 |
| \| *SPSB1* \| \| --- \| | ENSG00000171621.12 | chr1 |
| \| *SSBP3* \| \| --- \| | ENSG00000157216.14 | chr1 |
| \| *TBC1D2B* \| \| --- \| | ENSG00000167202.10 | chr15 |
| \| *TBL1Y* \| \| --- \| | ENSG00000092377.12 | chrY |
| \| *TBPL1* \| \| --- \| | ENSG00000028839.8 | chr6 |
| \| *TFEB* \| \| --- \| | ENSG00000112561.16 | chr6 |
| \| *TNFAIP8* \| \| --- \| | ENSG00000145779.7 | chr5 |
| \| *TNFRSF12A* \| \| --- \| | ENSG00000006327.12 | chr16 |

**Supplementary table 11 (continued):** List of TGF-β responsive genes (TBRS).

| Gene | Ensembl ID | Chromosome |
| --- | --- | --- |
| *TPM1* | ENSG00000140416.18 | chr15 |
| *TUFT1* | ENSG00000143367.14 | chr1 |
| *VEGFA* | ENSG00000112715.19 | chr6 |
| *YIPF5* | ENSG00000145817.15 | chr5 |
| *ZEB1* | ENSG00000148516.20 | chr10 |
| *ZFP36L1* | ENSG00000185650.9 | chr14 |
| *ADCK2*^†^ | ENSG00000133597.8 | chr7 |
| *ADRB2*^†^ | ENSG00000169252.5 | chr5 |
| *AGTR2*^†^ | ENSG00000180772.6 | chrX |
| *AHI1*^†^ | ENSG00000135541.19 | chr6 |
| *ANGEL2*^†^ | ENSG00000174606.11 | chr1 |
| *ARHGAP12*^†^ | ENSG00000165322.16 | chr10 |
| *ARID5B*^†^ | ENSG00000150347.13 | chr10 |
| *ATF7IP*^†^ | ENSG00000171681.11 | chr12 |
| *AVPI1*^†^ | ENSG00000119986.6 | chr10 |
| *BRDT*^†^ | ENSG00000137948.17 | chr1 |
| *CD163*^†^ | ENSG00000177575.11 | chr12 |
| *CD28*^†^ | ENSG00000178562.16 | chr2 |
| *CDKN2AIP*^†^ | ENSG00000168564.5 | chr4 |
| *CEBPD*^†^ | ENSG00000221869.4 | chr8 |
| *CITED2*^†^ | ENSG00000164442.9 | chr6 |
| *CKMT2*^†^ | ENSG00000131730.14 | chr5 |
| *CYBB*^†^ | ENSG00000165168.7 | chrX |
| *DDIT4*^†^ | ENSG00000168209.4 | chr10 |
| *DOPEY1*^†^ | ENSG00000083097.13 | chr6 |
| *FAT4*^†^ | ENSG00000196159.10 | chr4 |
| *FGB*^†^ | ENSG00000171564.10 | chr4 |
| *FLT4*^†^ | ENSG00000037280.14 | chr5 |
| *FLVCR2*^†^ | ENSG00000119686.8 | chr14 |
| *HNMT*^†^ | ENSG00000150540.12 | chr2 |
| *ID1*^†^ | ENSG00000125968.8 | chr20 |
| *IL5*^†^ | ENSG00000113525.8 | chr5 |
| *IRS1*^†^ | ENSG00000169047.5 | chr2 |
| *MAS1*^†^ | ENSG00000130368.5 | chr6 |
| *MTMR1*^†^ | ENSG00000063601.15 | chrX |
| *MYBL1*^†^ | ENSG00000185697.15 | chr8 |
| *MYC*^†^ | ENSG00000136997.13 | chr8 |
| *MYH11*^†^ | ENSG00000133392.15 | chr16 |
| *NR2F2*^†^ | ENSG00000185551.11 | chr15 |
| *OLIG2*^†^ | ENSG00000205927.4 | chr21 |
| *PAIP2B*^†^ | ENSG00000124374.8 | chr2 |
| *PNPLA4*^†^ | ENSG00000006757.10 | chrX |
| *RAB11FIP4*^†^ | ENSG00000131242.14 | chr17 |

^†^These genes are downregulated in response to TGF-β and included in the TBRS with a negative weight.

**Supplementary table 11 (continued):** List of TGF-β responsive genes (TBRS).

| Gene | Ensembl ID | Chromosome |
| --- | --- | --- |
| *RAI2*^†^ | ENSG00000131831.16 | chrX |
| *RNASE4*^†^ | ENSG00000258818.3 | chr14 |
| *SERTAD2*^†^ | ENSG00000179833.4 | chr2 |
| *SLC16A3*^†^ | ENSG00000141526.13 | chr17 |
| *SPP1*^†^ | ENSG00000118785.12 | chr4 |
| *SYCP1*^†^ | ENSG00000198765.10 | chr1 |
| *THPO*^†^ | ENSG00000090534.16 | chr3 |
| *UTP14A*^†^ | ENSG00000156697.11 | chrX |
| *ZNF318*^†^ | ENSG00000171467.14 | chr6 |
| *ZNF395*^†^ | ENSG00000186918.12 | chr8 |
| *ZNF44*^†^ | ENSG00000197857.12 | chr19 |

^†^These genes are downregulated in response to TGF-β and included in the TBRS with a negative weight.

**Supplementary table 12:** List of EMT pan-cancer signature genes.

| Gene | Ensembl ID | Chromosome |
| --- | --- | --- |
| *POSTN* | ENSG00000133110.13 | chr13 |
| *SCG5* | ENSG00000166922.7 | chr15 |
| *KANK4* | ENSG00000132854.17 | chr1 |
| *ADAM19* | ENSG00000135074.14 | chr5 |
| *LCE3D* | ENSG00000163202.4 | chr1 |
| *GPR68* | ENSG00000119714.9 | chr14 |
| *MMP9* | ENSG00000100985.7 | chr20 |
| *FN1* | ENSG00000115414.17 | chr2 |
| *ORM1* | ENSG00000229314.5 | chr9 |
| *SERPINB3* | ENSG00000057149.13 | chr18 |
| *ORM2* | ENSG00000228278.3 | chr9 |
| *KCNJ15* | ENSG00000157551.16 | chr21 |
| *DAB2* | ENSG00000153071.13 | chr5 |
| *HSD17B2* | ENSG00000086696.9 | chr16 |
| *SPINK6* | ENSG00000178172.5 | chr5 |
| *SAMSN1* | ENSG00000155307.16 | chr21 |
| *SERPINB4* | ENSG00000206073.9 | chr18 |
| *FAP* | ENSG00000078098.12 | chr2 |
| *ITGB3* | ENSG00000259207.6 | chr17 |
| *LBH* | ENSG00000213626.10 | chr2 |
| *IL7R* | ENSG00000168685.13 | chr5 |
| *PDE4DIP* | ENSG00000178104.18 | chr1 |
| *KCNMB4* | ENSG00000135643.4 | chr12 |
| *SERPINE1* | ENSG00000106366.8 | chr7 |
| *SULF1* | ENSG00000137573.12 | chr8 |
| *KLRC3*^†^ | ENSG00000205810.7 | chr12 |
| *SCNN1G*^†^ | ENSG00000166828.2 | chr16 |
| *DOK7*^†^ | ENSG00000175920.14 | chr4 |
| *CLDN1*^†^ | ENSG00000163347.5 | chr3 |
| *FGFBP1*^†^ | ENSG00000137440.4 | chr4 |
| *RAB17*^†^ | ENSG00000124839.11 | chr2 |
| *FGFR3*^†^ | ENSG00000068078.16 | chr4 |
| *LCN2*^†^ | ENSG00000148346.10 | chr9 |
| *SFTPD*^†^ | ENSG00000133661.14 | chr10 |
| *ENTPD2*^†^ | ENSG00000054179.10 | chr9 |
| *SYT8*^†^ | ENSG00000149043.15 | chr11 |
| *ADORA2B*^†^ | ENSG00000170425.3 | chr17 |
| *BBOX1*^†^ | ENSG00000129151.7 | chr11 |
| *METTL7A*^†^ | ENSG00000185432.11 | chr12 |
| *FXYD3*^†^ | ENSG00000089356.15 | chr19 |
| *SUSD2*^†^ | ENSG00000099994.10 | chr22 |
| *CYP4B1*^†^ | ENSG00000142973.11 | chr1 |
| *ADH1C*^†^ | ENSG00000248144.4 | chr4 |
| *MAL2*^†^ | ENSG00000147676.12 | chr8 |
| *TNNI2*^†^ | ENSG00000130598.14 | chr11 |

^†^These genes are downregulated during EMT and included in the EMT pan-cancer signature with a negative weight.

**Supplementary table 12 (continued):** List of EMT pan-cancer signature genes.

| Gene | Ensembl ID | Chromosome |
| --- | --- | --- |
| *ADH1A*^†^ | ENSG00000187758.6 | chr4 |
| *KRT13*^†^ | ENSG00000171401.13 | chr17 |
| *PHACTR3*^†^ | ENSG00000087495.15 | chr20 |
| *KRT15*^†^ | ENSG00000171346.12 | chr17 |

^†^These genes are downregulated during EMT and included in the EMT pan-cancer signature with a negative weight.
